# Electron Pool Enrichment of Polyhedral Carboranes: A Key to Isomerization and Nucleophilicity

**DOI:** 10.1021/acs.inorgchem.5c06016

**Published:** 2026-03-12

**Authors:** Vlastimil Němec, Josef Holub, Maksim A. Samsonov, Zdeňka Růžičková, Josef Cvačka, Jan Vrána, Aleš Růžička

**Affiliations:** a Department of General and Inorganic Chemistry, Faculty of Chemical Technology, 48252 University of Pardubice, Studentská 573, Pardubice 532 10, Czech Republic; b Institute of Inorganic Chemistry, Czech Academy of Sciences, Řež 250 68, Czech Republic; c Institute of Organic Chemistry and Biochemistry of the Czech Academy of Sciences, Flemingovo náměstí 542/2, Praha 6 166 10, Czech Republic

## Abstract

Carboranes are inorganic clusters exhibiting 3D aromaticity, which imparts exceptional thermal stability, low electrophilicity, and a large HOMO–LUMO gap. The isomerization of carboranes at ambient temperature remains, in many cases, a synthetic challenge. In this work, we present a new method based on the reactivity of *closo*-1,2-C_2_B_8_H_10_ with various types of carbenes. Such reactions yield *arachno*-shaped carboranes, which undergo facile dihydrogen elimination at low temperatures, accompanied by C–C bond cleavage and migration of the carbon atoms. These compounds react in the reverse manner compared to common neutral boranes, and their basicity is governed by the electronic parameters of the coordinated carbene molecules. Treatment with hydrogen chloride can thus leave the neutral carborane intact or protonate it once or even twice. The doubly protonated carboranes immediately undergo chlorination by the chloride anion. Thus, the carbene-carborane adducts undergo hydrogen-induced redox changes.

## Graphical abstract

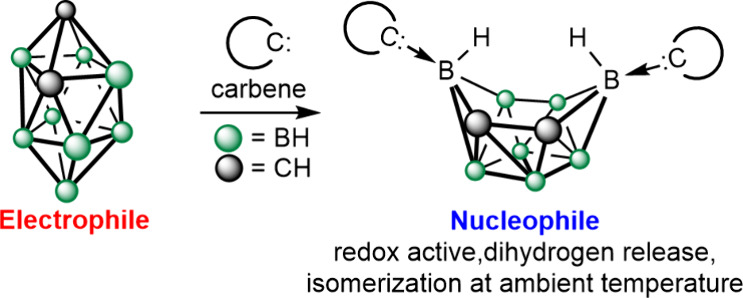

## Introduction

Carboranes are unique polyhedral compounds lying on the borderline between inorganic and organic chemistry. They exhibit three-dimensional aromaticity,^1–5^ which provides stability unmatched by other inorganic clusters. The most extensively studied carborane is icosahedral *closo*-1,2-C_2_B_10_H_12_ (*o*-carborane), which has found applications ranging from materials to medicinal chemistry.^6–8^ Its isomers (*m*- and *p*-carborane) have been explored to a much lesser extent despite the fact that they offer even higher stability, lower polarity, and distinct geometry and have been proven very useful, for example, as phenyl mimetics or ligand scaffolds.^9–13^ The major problem lies in their inaccessibility. The thermal isomerization of *o-*carborane to *m*- and *p*-carboranes has been the only production method requiring heating to 450 °C (*m*-) or 700 °C (*p*-) and carried out in autoclaves or tube-flow reactors.^6,14^ The isomerization of ten-vertex carboranes resembles that of the icosahedral ones, starting at 400 °C (Scheme 1).^15,16^ Moreover, it is impossible to proceed stepwise because *closo*-1,6-C_2_B_8_H_10_ is always accompanied by a small amount of its *p-*isomer, most likely because it is strongly thermodynamically favored and there is not as high barrier as in the 12-vertex analogues. Increasing the temperature even further yields exclusively the *p*-isomer. The mechanisms of these transformations have been thoroughly investigated by both experimental and theoretical methods, revealing diamond–square–diamond and triangular face rotation as plausible pathways.^17–21^ The only chemical isomerization within the cluster, described by Welch et al., has been the reduction of 12-vertex carboranes by sodium metal to various *nido-*type anions, which could be reoxidized to *o*- and *m*-carboranes.^22–24^ A similar situation has been observed for *o*- and *m*-C_2_B_8_H_10_, which can be mutually transformed by redox transformations involving *nido*-5,6-C_2_B_8_H_12_.^25^ This carborane can be further reduced to *arachno*-C_2_B_8_H_14_;^26^ however, the *closo*-1,10-C_2_B_8_H_10_ remained inaccessible by this approach. Therefore, cluster opening facilitates the isomerization process.

**Scheme 1:**
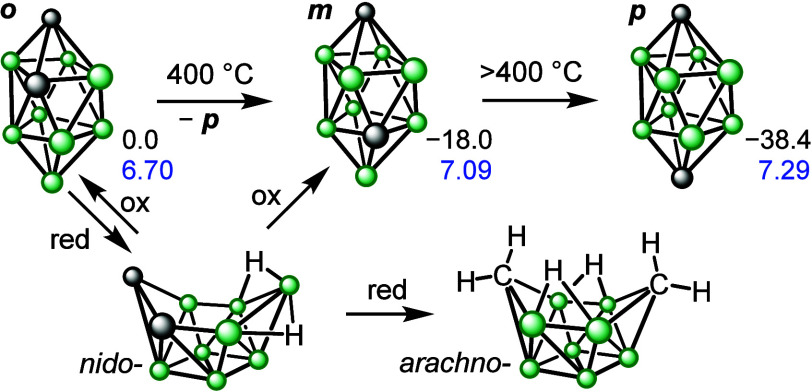
Isomerization of Ten-Vertex *o*-, *m*-, and *p*-*closo*-Carboranes by Heating or by Reduction (red) and Oxidation (ox)^a^

a The free Gibbs energies (in black, kcal/mol) and HOMO–LUMO gaps (in blue, eV) were calculated at the B3LYP-D3­(BJ)/def2-TZVP level of theory.

Reactions of carboranes with strong nucleophiles can follow different pathways, ranging from simple coordination to boron atom extrusion (Scheme 2).^6^ Carbenes represent a special class of bases capable of cleaving almost any bond within the cluster with the exception of the C–C bond. Reaction with *in situ* generated carbethoxycarbene proceeded via insertion into the B–H bond, yielding all possible isomers.^27,28^ Small aliphatic *N*-heterocyclic carbenes (NHCs) can deprotonate the C–H group or remove a boron atom to an exoskeletal position, depending on the substrate and reaction conditions.^29,30^ Bulky aromatic NHCs do not allow coordination of a second carbene molecule to a single boron atom and therefore yield 1:1 adducts regardless of the stoichiometry and conditions used.^31^ We have recently shown that heteroboranes can be opened by NHCs, forming *nido-* or *arachno-*shaped cluster adducts (Scheme 2).^32,33^ These compounds exhibit reversed polarity in comparison with conventional neutral boranes and react with acids to produce thermally robust, positively charged heteroboranes, which were previously elusive or thermally unstable.^34^ Moreover, the NHC–carborane adducts decompose within several weeks at 120 °C in a sealed tube in THF solution to yield *p*-carborane regardless of the starting isomer used. We have thus extended this concept with utilization of different carbene bases to access new routes to ten-vertex carborane derivatives and new reactivity toward acids.

**Scheme 2:**
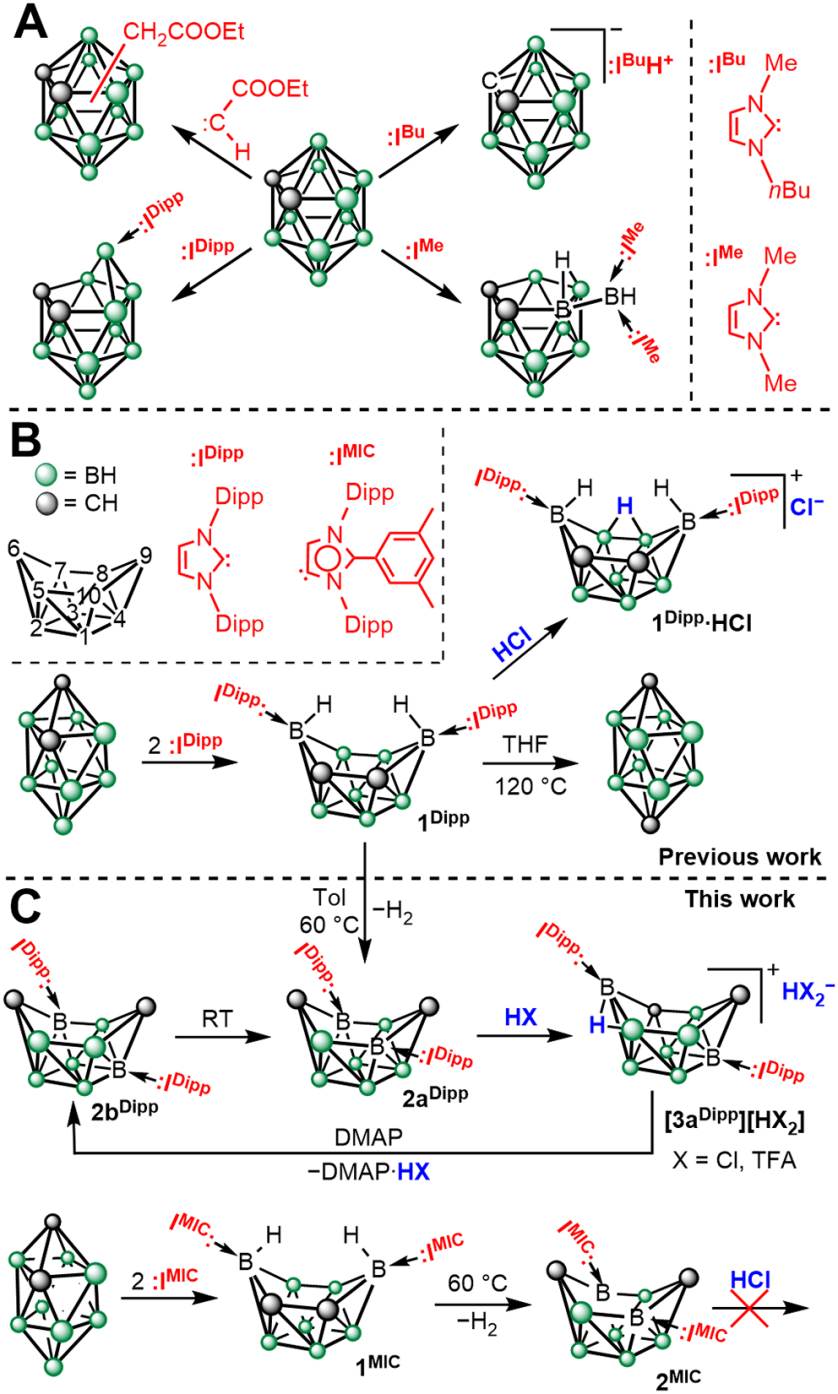
Published Reactivity of Carboranes with Carbenes (**A**). The Synthesis of **1** ^**Dipp**^ and **1** ^**MIC**^, Their Thermal Decomposition and Reactivity (**B**, **C**)­^a^

a TFA = trifluoroacetate; Dipp = 2,6-diisopropylphenyl.

## Results and Discussion

We started with the previously published^32^ **1** ^**Dipp**^ and tested its thermal stability in noncoordinating solvents and acetonitrile, which surprisingly revealed a quantitative transformation to **2a** ^**Dipp**^ even at 90 °C within 16 h. Interestingly, the reaction proceeds also in bis­(2-methoxyethyl)­ether unlike the THF, which allowed further heating at 210 °C, shortening the time required for decomposition to *p*-C_2_B_8_H_10_ to 1 week. The transformation to **2a** ^**Dipp**^ involves dihydrogen elimination and multiple shifts in the six-membered C_2_B_4_ cycle, including C–C bond cleavage and migration of the carbon atoms to the remote vertices of the boat-shaped cluster. Despite the fact that it must proceed through several steps, we have observed only **1** ^**Dipp**^ and **2a** ^**Dipp**^ in the ^11^B NMR spectrum of the reaction mixture, and thus, we have decided to investigate the mechanism theoretically. The reaction begins with the elimination of dihydrogen, leaving the NHC-bearing boron atoms unsaturated, and then moves to a cage-closure. Several transition states result in the formation of another cavity, leading to the cleavage of the C–C bond and the restoration of the boat-shape (for more information, see the SI, Figure S89).

According to Wade’s rules,^35–37^ the reaction of *closo*-1,2-C_2_B_8_H_10_ with NHCs changes the arrangement to the *arachno-*type, as it increases the number of SEPs by two. The B–C interaction can be described as a strong coordination bond because the carbene remains neutral, as demonstrated previously by both spectroscopic and theoretical studies.^32^ However, the elimination of dihydrogen represents an oxidation to the *nido-*type, decreasing the SEP count of **2a** ^**Dipp**^ by one. This affects the basicity compared to **1** ^**Dipp**^, which acts as a superbase, as we have recently demonstrated.^38^ Thus, the reactions with weak acids such as acetylacetone, dimethylmalonate, and nitromethane have left **2a** ^**Dipp**^ intact. In contrast, the reaction with strong acids (HCl, CF_3_COOH) forces another rearrangement to **[3a** ^**Dipp**^ **]** ^**+**^, moving formally a carbon atom around the upper rim. Unlike **1** ^**Dipp**^ **·HCl**, **[3a** ^**Dipp**^ **]** ^**+**^ can be easily deprotonated using DMAP to **2a** ^**Dipp**^. In the reaction mixture, we have detected another isomer, **2b** ^**Dipp**^, which is thermally unstable and transforms to **2a** ^**Dipp**^ even at room temperature. The relative free Gibbs energies (Figure S91) nicely support both the reversibility of **2a** ^**Dipp**^ (0.0 kcal/mol) and **[3a** ^**Dipp**^ **]** ^**+**^ (0.6 kcal/mol) protonation/deprotonation of the conjugated base (**2a** ^**Dipp**^)/acid (**[3a** ^**Dipp**^ **]** ^**+**^) as well as the instability of **2b** ^**Dipp**^ (5.0 kcal/mol). The driving force of the deprotonation is obviously the formation of DMAP·HCl.

The molecular structures of **2a** ^**Dipp**^, **2b** ^**Dipp**^, **[3a** ^**Dipp**^ **]­[HCl**_**2**_ **]**, and **[3a** ^**Dipp**^ **]­[H­(TFA)**_**2**_ **]** have been confirmed by both multinuclear NMR (see the SI, Table S1 and Figure S86) and X-ray diffraction analysis (Figures 1 and 2). The molecular structures of **2a** ^**Dipp**^ and **2b** ^**Dipp**^ have revealed the boat shape of the central cluster, with B–B (1.856(4)–1.8900(16) Å) and B–C (1.483(4)–1.549(4) Å) separations of the upper rim falling within a narrow range. Both carboranes resemble earlier published *nido-*clusters, e.g., 5,6-Me_2_-*nido*-5,6-C_2_B_8_H_9_^–^,^39^ although the position of the carbon atoms in positions 6 and 9 has so far been observed only for *arachno-*derivatives (*arachno-*6,9-C_2_B_8_H_14_).^26^ The molecular structures of **[3a** ^**Dipp**^ **]­[HCl**_**2**_ **]** and **[3a** ^**Dipp**^ **]­[H­(TFA)**_**2**_ **]** have exhibited slight deformation of the cluster backbone (for more information, see the SI).

**Figure 1:**
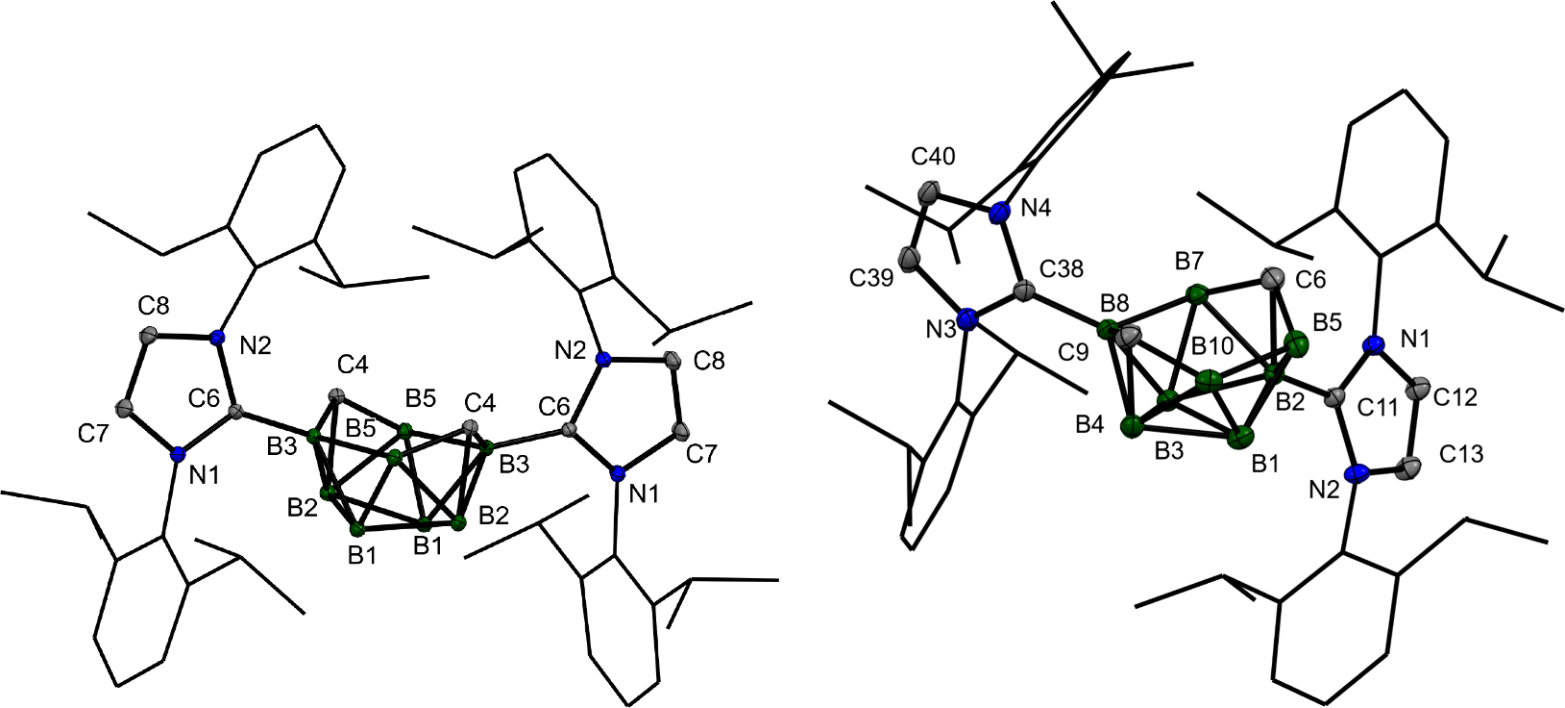
Molecular structures of **2a** ^**Dipp**^ (left, crystallographic numbering applied for symmetry reasons) and **2b** ^**Dipp**^ (right). ORTEP diagrams, 40% probability level; Dipp groups are shown as wireframes for clarity. Selected interatomic distances [Å]: **2a** ^**Dipp**^ B3–C4 1.5255(14), C4–B5 1.5229(14), B5–B3 1.8900(16), B3–C6 1.5846(14); **2b** ^**Dipp**^ B5–C6 1.483(4), C6–B7 1.500(4), B7–B8 1.856(4), B8–C9 1.549(4), C9–B10 1.533(4), B10–B5 1.866(4), B2–C11 1.577(3), B8–C38 1.586(3).

**Figure 2:**
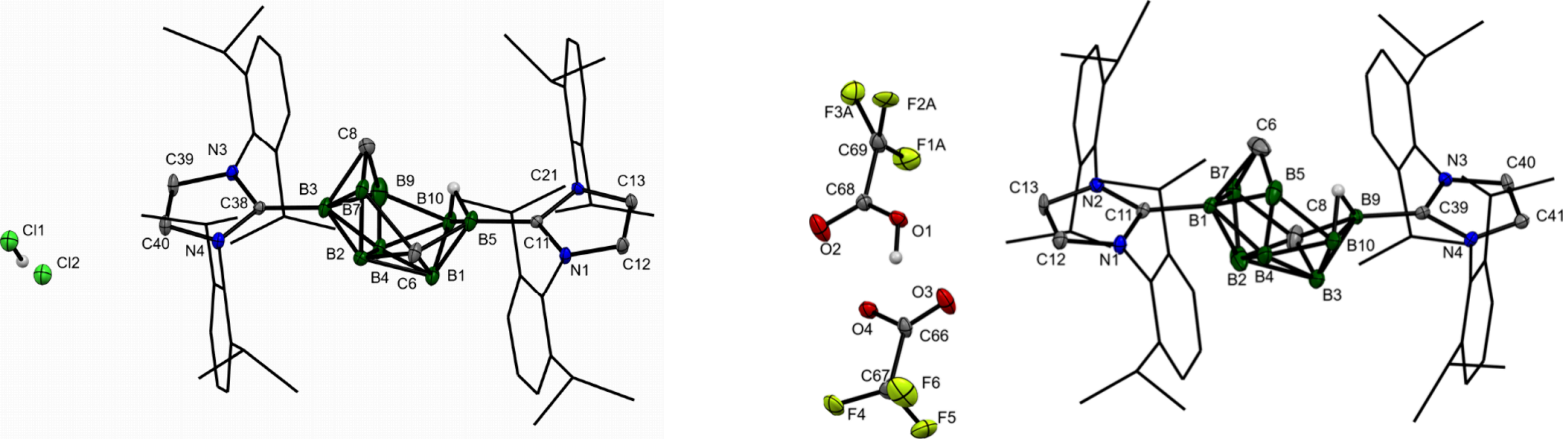
Molecular structures of **[3a** ^**Dipp**^ **]­[HCl**_2_ **]** (left, crystallographic numbering applied for symmetry reasons) and **[3a** ^**Dipp**^ **]­[H­(TFA)**_2_ **]** (right). ORTEP diagrams, 40% probability level; Dipp groups are shown as wireframes for clarity. Selected interatomic distances [Å]: **[3a** ^**Dipp**^ **]­[HCl**_2_ **]** B5–C6 1.760(10), C6–B7 1.937(7), B7–C8 1.532(10), C8–B9 1.710(10), B9–B10, 1.776(9), B10–B5 1.743(8), B5–C11 1.575(9), B3–C38 1.578(8); **[3a** ^**Dipp**^ **]­[H­(TFA)**_2_ **]** B5–C6 1.643(15), C6–B7 1.725(14), B7–C8 1.845(13), C8–B9 1.694(13), B9–B10 1.71(2), B9–H10′ 1.23(4), B10–H10′ 1.26(4), B9–C39 1.559(11), B1–C11 1.582(10).

To investigate the influence of the electronic and steric properties of the carbene on the overall reactivity of the carborane *closo*-1,2-C_2_B_8_H_10_, we employed a mesoionic carbene (**:I^MIC^**) as a better π-electron acceptor (Scheme 2) compared to **:I^Dipp^**, and **1** ^**MIC**^ was prepared by direct synthesis using the same procedure as that for **1** ^**Dipp**^. Heating **1** ^**MIC**^ in both THF and acetonitrile has led to the elimination of dihydrogen and the formation of **2** ^**MIC**^ rather than decomposition to *closo*-1,10-C_2_B_8_H_10_, which did not occur even after heating to 150 °C. Both **1** ^**MIC**^ and **2** ^**MIC**^ exhibit the expected patterns in NMR spectra, and their molecular structures have been confirmed by X-ray diffraction analysis (for more information, see the SI). Unlike **2a** ^**Dipp**^, **2** ^**MIC**^ does not react with either hydrogen chloride or trifluoroacetatic acid, most likely reflecting the higher electron-acceptor properties of mesoionic carbenes, which decrease the basicity of the carborane cluster. Therefore, we tested an aliphatic NHC (Scheme 3, **:I^*i*Pr^**) as a stronger σ-electron donor to enhance the reactivity instead.

**Scheme 3:**
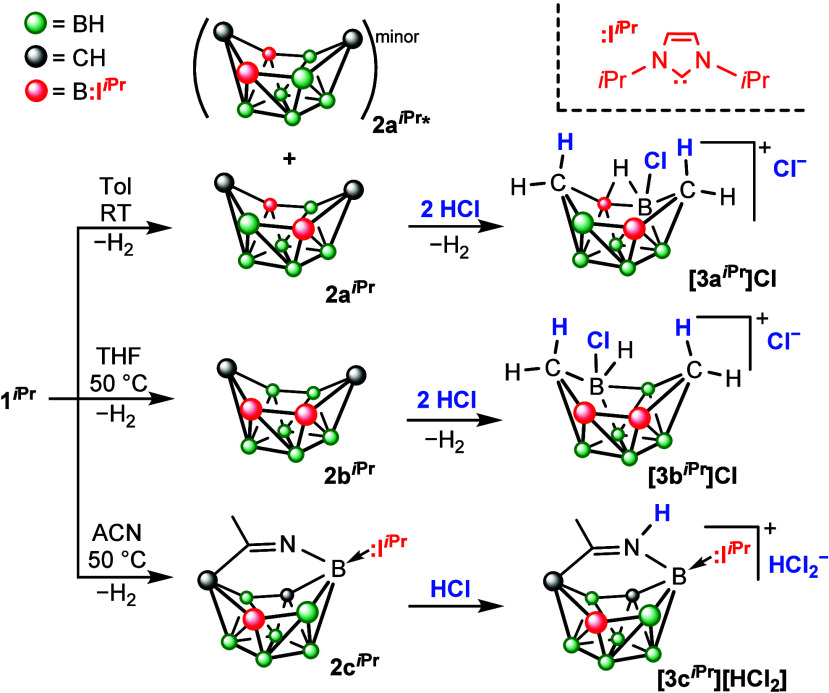
Synthesis and Thermal Decomposition of Carborane–NHC Adducts as Well as Their Reactivity with HCl^a^

a ACN = acetonitrile.

The reaction of *o*-C_2_B_8_H_10_ with :**I^*i*Pr^** proceeded without deprotonation and/or boron atom extrusion as in the case of the 12-vertex analogues. The carborane **1** ^***i*** **Pr**^ slowly undergoes dihydrogen elimination even in the solid state at room temperature, yielding isomers **2a** ^***i*** **Pr**^ and **2a** ^***i*** **Pr**^ * (Scheme 3). This process can be accelerated by heating the sample in toluene at 90 °C overnight. The optimization of the reaction conditions has not significantly affected the ratio between the isomers, as a result of which the minor **2a** ^***i*** **Pr**^ * (up to 7%) could not be isolated. Using THF, we observed the formation of another isomer, **2b** ^***i*** **Pr**^, bearing the NHCs at neighboring positions. This reaction proceeds slowly at room temperature, while heating to 50 °C leads to full conversion overnight. Interestingly, heating to 90 °C results in the decomposition of **2b** ^***i*** **Pr**^ to several compounds, with **2a** ^***i*** **Pr**^ as the major product. The experiment is consistent with the calculated free Gibbs energies, which have confirmed that the isomers **2a** ^***i*** **Pr**^ and **2a** ^***i*** **Pr**^ * are thermodynamically more favorable (−36.4 and −34.5 kcal/mol) than **2b** ^***i*** **Pr**^ (−21.3 kcal/mol) and even more favorable than the parent **1** ^***i*** **Pr**^ (0.0 kcal/mol) or the intermediate after dihydrogen elimination (+8.3 kcal/mol). The formation of three different isomers is enabled by considerably lower steric parameters of the **:I^*i*Pr^** groups compared to those of **:I^Dipp^** and **:I^MIC^**. It has been shown earlier that two molecules of **:I^Dipp^** can be coordinated neither to a single skeletal atom^31^ nor the adjacent boron atoms.^32^ Heating **1** ^***i*** **Pr**^ in acetonitrile surprisingly resulted in the addition of the solvent molecule across the cluster, bridging the distal vertices of the six-membered rim. To the best of our knowledge, this is only the second example of such an addition at a borane cluster.^40^ It is worth noting that only one of the carbon atoms has changed its position relative to the boron atoms, which indicates that the addition takes place with an intermediate between **1** ^***i*** **Pr**^ and **2a** ^***i*** **Pr**^. This has been confirmed by heating both **2a** ^***i*** **Pr**^ and **2b** ^***i*** **Pr**^ in acetonitrile, which has not resulted in solvent addition. From the perspective of Wade’s rules, the formation of compounds **2a** ^***i*** **Pr**^ and **2b** ^***i*** **Pr**^ is again an *arachno-* to *nido-* conversion. In contrast, the reaction sequence yielding **2c** ^***i*** **Pr**^ is a rare example of a direct formation of an *arachno-*arrangement.^41^

The ^11^B NMR spectra of **2a** ^***i*** **Pr**^, **2b** ^***i*** **Pr**^, and **2c** ^***i*** **Pr**^ have revealed the expected patterns with respect to their structures and correlate well with previously published compounds (for more information, see Figure S86). The molecular structures of **2a** ^***i*** **Pr**^–**2c** ^***i*** **Pr**^ have been confirmed by X-ray diffraction analysis (Figure 3). Only negligible differences have been observed in the geometrical parameters of the carborane clusters in **2a** ^***i*** **Pr**^ *, **2a** ^***i*** **Pr**^, and **2b** ^***i*** **Pr**^. In contrast, the C=N moiety slightly (∼0.2 Å) shortens the separation between the bridged atoms (positions 6 and 9) in **2c** ^***i*** **Pr**^, and its length (1.269(2) Å) corresponds to a double bond (Σ_cov_(C=N) = 1.27 Å).^42^

**Figure 3:**
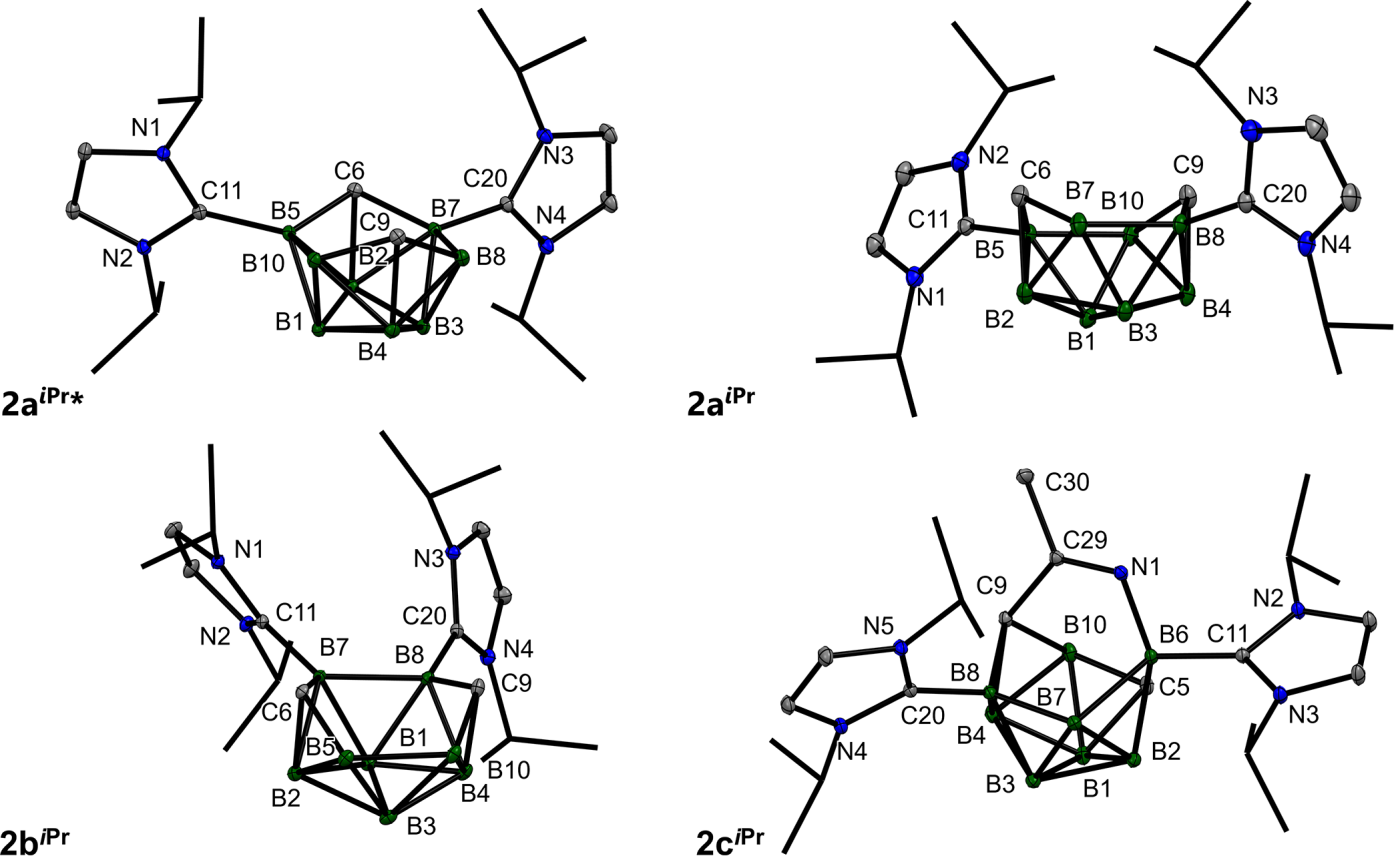
Molecular structures of **2a** ^***i*** **Pr**^ *, **2a** ^***i*** **Pr**^, **2b** ^***i*** **Pr**^, and **2c** ^***i*** **Pr**^. ORTEP diagrams, 40% probability level; *i*Pr groups are shown as wireframes. Solvent molecules have been omitted for the sake of clarity. Selected interatomic distances [Å]: **2a** ^***i*** **Pr**^ * B5–C6 1.5285(19), C6–B7 1.5293(17), B7–B8 1.8859(19), B8–C9 1.5348(18), C9–B10 1.5311(18), B10–B5 1.8720(19), B5–C11 1.5849(17), B7–C20 1.5875(17); **2a** ^***i*** **Pr**^ B5–C6 1.524(3), C6–B7 1.549(3), B7–B8 1.863(3), B8–C9 1.541(3), C9–B10 1.531(3), B10–B5 1.868(3), B5–C11 1.578(3), B8–C20 1.588(3); **2b** ^***i*** **Pr**^ B5–C6 1.5201(18), C6–B7 1.5495(18), B7–B8 1.8931(19), B8–C9 1.5461(18), C9–B10 1.527(2), B10–B5 1.887(2), B7–C11 1.6061(17), B8–C20 1.5931(18); **2c** ^***i*** **Pr**^ C5–B6 1.7070(19), B6–B7 1.8868(19), B7–B8 1.6957(19), B8–C9 1.6973(18), C9–B10 1.7183(19), B6–C11 1.6355(18), B10–C5 1.5892(19), B6–C11 1.6355(18), B8–C20 1.5883(2), B6–N1 1.5274(17), N1–C29 1.2695(16), C29–C9 1.5177(17).

The reaction of **2a** ^***i*** **Pr**^ and **2b** ^***i*** **Pr**^ with hydrogen chloride has, surprisingly, not proceeded as a simple protonation. Instead, double protonation of the carbon atoms and chlorination of the B5 atom have taken place, yielding positively charged **[3a** ^***i*** **Pr**^ **]** ^**+**^ and **[3b** ^***i*** **Pr**^ **]** ^**+**^, which are formally derived from *arachno*-6,9-C_2_B_8_H_14_.^26^ To the best of our knowledge, this is the first chlorination of polyhedral boranes by hydrogen chloride at ambient temperature. Standard procedures involve highly reactive chlorination agents (chlorine, chlorosuccinimide) or require harsh reaction conditions.^6^ A key difference is the reversed polarity of carboranes **2a** ^***i*** **Pr**^ and **2b** ^***i*** **Pr**^, which are able to accept a proton, forming positively charged compounds, which were elusive until very recently. The most plausible mechanism involves the initial double protonation of CH groups (Scheme 3), yielding a dicationic intermediate, which immediately reacts with chloride to form the final compounds. In order to test this, we have treated **2b** ^***i*** **Pr**^ with excess DCl and observed the expected absence of two signals corresponding to CH groups oriented inside the six-membered rim (*endo*-CH) in the ^1^H NMR spectrum (δ­(^1^H) = 1.00, 1.58, and 2.05 ppm, Figures S71–S74). The double protonation is consistent with the concept that the basicity of carborane increases with stronger carbene donors. In terms of the mechanism, the reaction is a proton-coupled electron transfer because the polarity induced by the protonation inflicts the reduction by the chloride atom. In contrast, the reaction of **2c** ^***i*** **Pr**^ with hydrogen chloride has resulted in the expected protonation of the imine moiety, even in the presence of excess acid. The carborane backbone in **2c** ^***i*** **Pr**^ is already an *arachno*-arrangement and thus cannot accept another proton. The reactivity of **2a** ^***i*** **Pr**^ and **2b** ^***i*** **Pr**^ can be explained by the narrowing of the HOMO–LUMO gap to 4.3 and 4.0 eV (Figure S91), which are values comparable to other reactive main-group molecules capable of undergoing oxidative additions, e.g., carbenes. Parent ten-vertex carboranes exhibit much broader gaps (6.7 (*o*-), 7.1 (*m*-), and 7.3 (*p*-) eV), with HOMOs inaccessible to any acid. Our approach exploits the electron density inside the cluster in order to modify both the structural features of carboranes and their reactivity.

The ^1^H NMR spectra of **[3a** ^***i*** **Pr**^ **]** ^**+**^ and **[3b** ^***i*** **Pr**^ **]** ^**+**^ have revealed a typical AX pattern for CH_2_ groups (δ­(^1^H) = 0.12–2.64 ppm), upfield-shifted with respect to *arachno*-6,9-C_2_B_8_H_14_ and its neutral (δ­(^1^H) = −0.67–0.86 ppm) or anionic (δ­(^1^H) = −1.57–(−0.46) ppm) derivatives,^26^ which can be attributed to the presence of positive charge within the cluster. Unlike for **[3b** ^***i*** **Pr**^ **]** ^**+**^, we have observed a signal for a B*H*B (δ­(^1^H) = −1.64 ppm) bridge in the spectrum of **[3a** ^***i*** **Pr**^ **]** ^**+**^, which is highly downfield-shifted when compared to other compounds such as **1** ^**Dipp**^ **·**HCl^32^ or **[3a** ^**Dipp**^ **]** ^**+**^ (−3.38–(−4.14) ppm) and lies closer to terminal BH groups.

The molecular structures of **[3b** ^**Pr**^ **]­Cl** and **[3c** ^***i*** **Pr**^ **]­[HCl**_**2**_ **]**, as revealed by X-ray diffraction analysis (Figure 4), confirmed the *arachno-*arrangement of both carboranes. The overall cluster geometries show only slight differences and resemble the previously published *arachno*-6,9-C_2_B_8_H_14_.^26^ Both anions exhibit weak interactions with the most protic hydrogen atoms of the clusters (*endo*-C*H* in **[3a** ^***i*** **Pr**^ **]­Cl** = 2.781 and 2.789 Å; N–H in **[3c** ^***i*** **Pr**^ **]­[HCl**_**2**_ **]** 2.404 Å).

**Figure 4:**
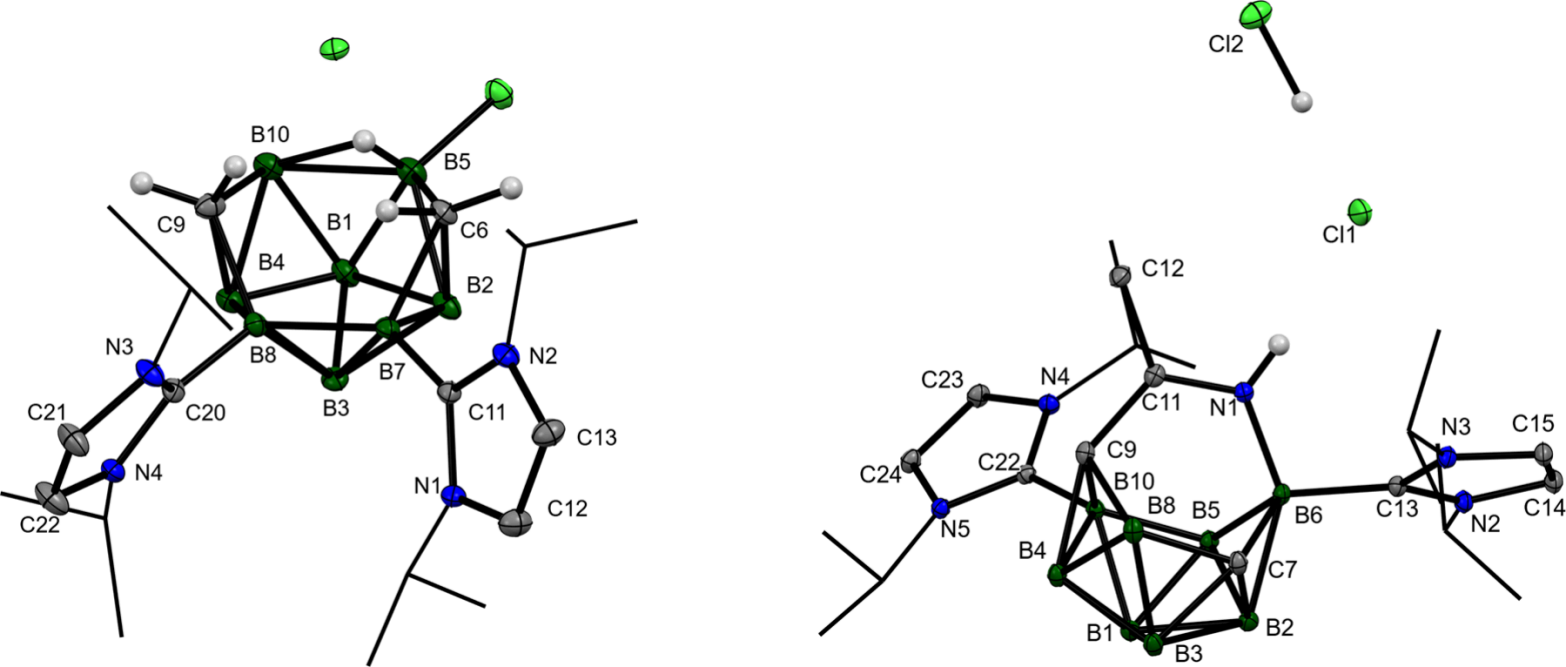
Molecular structures of **[3b** ^***i*** **Pr**^ **]­Cl** (left) and **[3c** ^***i*** **Pr**^ **]­[HCl**_2_ **]** (right). An ORTEP diagram, 40% probability level; *i*Pr groups are shown as wireframes. The solvent molecules (acetonitrile and tetrahydrofuran) were omitted for clarity. Selected interatomic distances [Å]: **[3b** ^***i*** **Pr**^ **]­Cl** B5–C6 1.680(17), C6–B7 1.728(15), B7–B8 1.728(15), B8–C9 1.729(16), C9–B10 1.699(18), B5–B10 1.866(19), B5–H5′ 0.89, B10–H5′ 1.48, B7–C11 1.610(15), B8–C20 1.586(16), B5–BCl1 1.818(14); **[3c** ^***i*** **Pr**^ **]­[HCl**_2_ **]** B5–B6 1.889(2), B6–C7 1.6851(19), C7–B8 1.5873(19), B8–C9 1.747(2), C9–B10 1.7356(19), B10–B5 1.690(2), B6–C13 1.6246(18), B10–C22 1.5923(18), B6–N1 1.5373(18), N1–C11 1.2923(17), C11–C9 1.4611(18).

## Conclusions

In summary, we have demonstrated that carbenes can modify ten-vertex carboranes by opening them to *arachno*-forms, which are much more reactive and undergo dihydrogen elimination at ambient temperature, breaking the C–C bond and forming various isomers. This concept of opening carboranes by increasing the SEP count without changing the charge of the cluster facilitates isomerization and unlocks the nucleophilicity. The basicity and reactivity of the resulting *nido*-carboranes depend on the electronic properties of the carbenes and have been tested by treatment with hydrogen chloride. The mesoionic carbenes in **2** ^**MIC**^, as the best π-electron acceptors among the tested carbenes, do not allow for any interaction with the acidic proton. On the contrary, **2a** ^**Dipp**^, bearing NHCs with aromatic substituents, enables protonation, which induces another reisomerization but can be reversed by base addition. Carboranes with aliphatic NHCs undergo acetonitrile or hydrogen-chloride addition, forming *arachno-*clusters. Such reactivity is not typical of polyhedral boranes and is mediated by the accessibility of both the HOMO and LUMO of the NHC–carborane adducts. The possibility of redox transformations induced by the addition of a proton opens space for new synthetic applications of carboranes. This work demonstrates that carboranes are not electron-deficient, as stated in many inorganic chemistry textbooks.^53^ On the contrary, they possess internal electron density that can be readily exploited. This concept could be further used in the chemistry of 12-vertex carboranes, the most extensively studied group of boron clusters. In the future, this approach could be promising for the practical synthesis of different isomers of *closo*-carboranes, while the key lies in finding a base that can be more easily removed.

## Experimental Section

No uncommon hazards are noted.

### Materials and Methods

All air- and moisture-sensitive manipulations were carried out under an argon atmosphere using standard Schlenk-tube techniques. Solvents were dried using PureSolv-Innovative Technology equipment under an argon atmosphere. Deuterated solvents were purchased from Euriso-Top GmbH. The starting compounds 1,2-C_2_B_8_H_10_,^25^ **1** ^**Dipp**^,^32^ **:I^*i*Pr^**,^43^ **:I^Dipp^**,^44^ and **:I^MIC^** ^45^ were prepared according to published procedures. ^1^H, ^11^B, and ^13^C­{^1^H} NMR spectra were recorded on a Bruker Avance I 500 MHz spectrometer or a Bruker Ultrashield 400 MHz spectrometer using a 5 mm tunable broad-band probe. Appropriate chemical shifts in ^1^H and ^13^C­{^1^H} NMR spectra were referenced to the residual signals of the solvent (C_6_D_6_: δ­(^1^H) = 7.18 ppm, δ­(^13^C) = 128.39 ppm; CD_3_CN: δ­(^1^H) = 1.94 ppm, δ­(^13^C) = 1.32 ppm; CD_2_Cl_2_: δ­(^1^H) = 5.32 ppm, δ­(^13^C) = 54.00 ppm; THF-D_8_: δ­(^1^H) = 3.58 ppm, δ­(^13^C) = 67.21 ppm; CD_3_OD: δ­(^1^H) = 3.31 ppm, δ­(^13^C) = 49.00 ppm). The general numbering of the clusters is depicted in Figure 5.

**Figure 5:**
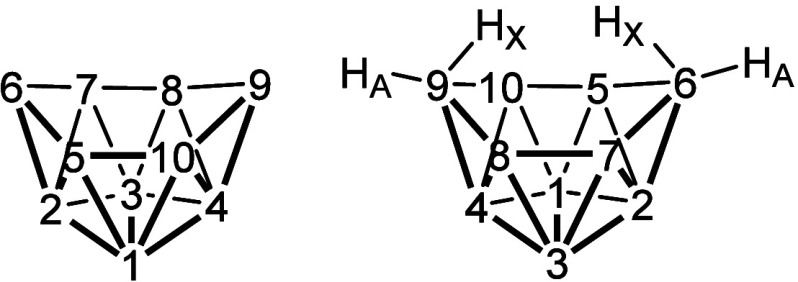
General numbering of the prepared *nido-* and *arachno-*carboranes.

### Crystallography

Full sets of diffraction data for **1** ^**MIC**^ (2514071), **2a** ^**Dipp**^ (2514074), **2b** ^**Dipp**^ (2514070), **2a** ^***i*** **Pr**^ (2514068), **2a** ^***i*** **Pr**^ * (2514067), **2b** ^***i*** **Pr**^ (2514066), **2c** ^***i*** **Pr**^ (2514072), **2** ^**MIC**^ (2514069), **[3a** ^**Dipp**^ **]­[HCl**_**2**_ **]** (2514065), **[3a** ^**Dipp**^ **]­[H­(TFA)**_**2**_ **]** (2514064), **[3b** ^***i*** **Pr**^ **]­Cl** (2514063), and **[3c** ^***i*** **Pr**^ **]­[HCl**_**2**_ **]** (2514073) were collected at 150(2) K with a Bruker D8-Venture diffractometer equipped with Mo (Mo/K_α_ radiation; λ = 0.71073 Å) microfocus X-ray (IμS) sources and Photon CMOS detector, and an Oxford Cryosystems cooling device was used for data collection. The frames were integrated with the Bruker SAINT software package by using a narrow-frame algorithm. Data were corrected for absorption effects using the multiscan method (SADABS). The obtained data were treated by XT-version 2014/5 and SHELXL-2017/1 software implemented in the APEX3 v2016.5–0 (Bruker AXS) system.^46^

Hydrogen atoms were mostly localized on a difference Fourier map, but to ensure uniformity of the treatment of the crystal, all hydrogen atoms were recalculated into idealized positions (riding model) and assigned temperature factors *H*_iso_(H) = 1.2*U*_eq_ (pivot atom) or 1.5*U*_eq_ (methyl). H atoms in methyl, methylene, and moieties and hydrogen atoms in aromatic rings were placed with C–H distances of 0.96, 0.97, and 0.93 Å, and terminal B–H groups in polyhedral borane were placed with a distance of 1.1 Å. Hydrogen atoms in N–H and some B–H bonds were refined freely, and the B–H distance of bridging H was refined freely or maintained to 1.25 Å.

*R*_int_ = ∑|*F*_0_^2^ – *F*_o,mean_^2^|/∑*F*_0_^2^, GOF = [∑(*w*(*F*_0_^2^ – *F*_c_^2^)^2^)/(*N*_diffrs_ – *N*_params_)]^1/2^ for all data, *R*(*F*) = ∑||*F*_0_| – |*F*_c_||/∑|*F*_0_| for observed data, *wR*(*F* ^2^) = [∑(*w*(*F*_0_^2^ – *F*_c_^2^)^2^)/(∑*w*(*F*_0_^2^)^2^)]^1/2^ for all data.

Crystallographic data for structural analysis have been deposited with the Cambridge Crystallographic Data Centre, CCDC. Copies of this information may be obtained free of charge from The Director, CCDC, 12 Union Road, Cambridge CB2 1EY, UK (fax: + 44–1223–336033; e-mail: deposit@ccdc.cam.ac.uk or http://www.ccdc.cam.ac.uk).

Mass spectra were acquired on an Orbitrap IQ-X Tribrid mass spectrometer (Thermo Fisher Scientific, Waltham, MA, USA) operated in positive ion mode at a resolving power of 500,000. The instrument was equipped with a heated electrospray ionization (HESI) source, set to 275 °C. The spray voltage was maintained at 3.5 kV, and the ion transfer tube temperature was set to 300 °C. Samples were prepared in acetonitrile and introduced into the system via a 10 μL injection loop. The mobile phase consisted of acetonitrile delivered at a flow rate of 150 μL/min. Mass calibration was performed automatically using the instrument’s Auto-Ready ion source feature.

### DFT Calculations

All the calculations were performed with the Gaussian 16 program.^47^ The energetic profiles of the reactions were computed at the B3LYP/def2-TZVP level of theory,^48–50^ including solvation effects, where the polarizable continuum model (PCM)^51^ was used for toluene. Dispersion corrections were also considered, employing the D3 version of Grimme’s dispersion method.^52^ All computed structures were confirmed to be minima on the potential energy surface through frequency analysis at the same level of theory with transition states exhibiting only one imaginary frequency.

### Synthesis of **2a^Dipp^**

A solution of **1** ^**Dipp**^ (3.10 g, 3.5 mmol) in toluene (30 mL) was heated to 90 °C, and the mixture was stirred for 16 h. The volatiles were removed in vacuo, and the solid residue was washed with hexane (2 × 20 mL), yielding **2a** ^**Dipp**^ as a white powder. Yield: 2.7 g, 86%. Mp. 150 °C. ESI-MS (*m*/*z* value): calcd. for C_56_H_81_N_4_B_8_^+^: 897.72002; found 897.72048 [Fragment: (M–H)^+^]. ^1^H NMR (25 °C, C_6_D_6_, 500 MHz): δ = 0.94 (d, ^3^ *J*(^1^H, ^1^H) = 6.9 Hz, 12H, CH­(C*H*_3_)_2_), 1.02 (d, ^3^ *J*(^1^H, ^1^H) = 6.9 Hz, 12H, CH­(C*H*_3_)_2_), 1.05 (d, ^3^ *J*(^1^H, ^1^H) = 6.6 Hz, 12H, CH­(C*H*_3_)_2_), 1.40 (d, ^3^ *J*(^1^H, ^1^H) = 6.7 Hz, 12H, CH­(C*H*_3_)_2_), 2.86 (sept, ^3^ *J*(^1^H, ^1^H) = 6.7 Hz, 4H, C*H*(CH_3_)_2_), 3.02 (sept, ^3^ *J*(^1^H, ^1^H) = 6.8 Hz, 4H, C*H*(CH_3_)_2_), 4.46 (s, 2H, BC*H*), 6.27 (s, 4H, C*H*=C*H*), 7.02 (d, ^3^ *J*(^1^H, ^1^H) = 7.64 Hz, 4H, *m*-C_6_ *H*_3_), 7.08 (d, ^3^ *J*(^1^H, ^1^H) = 7.7 Hz, 4H, *m*-C_6_ *H*_3_), 7.20 (t, ^3^ *J*(^1^H, ^1^H) = 7.7 Hz, 4H, *p*-C_6_ *H*_3_) ppm. ^11^B NMR (25 °C, C_6_D_6_, 160.46 MHz) δ = −29.5 (d, ^1^ *J*(^1^H, ^11^B) = 133.8 Hz, 2B, B1,3), −9.3 (s broad, 2B, B8,5), −6.0 (s broad, 2B, B7,10), −3.9 (broad, 2B, B2,4) ppm. ^13^C­{^1^H} NMR (25 °C, C_6_D_6_, 125.76 MHz): δ = 23.1, 23.3, 25.8, 26.1 (CH­(*C*H_3_)_2_), 28.8, 28.9 (*C*H­(CH_3_)_2_), 119.0 (s broad, B*C*H), 123.17 (*C*H=*C*H), 124.0 (*m*-C_6_H_3_), 124.1 (*m*-C_6_H_3_), 130.2 (*p*-C_6_H_3_), 135.4 (*ipso*-C_6_H_3_), 146.0 (*o*-C_6_H_3_), 146.1 (*o*-C_6_H_3_), 166.7 (broad, N*C*N) ppm.

### Synthesis of **[3a^Dipp^]­[HCl_2_]**

A solution of hydrogen chloride (0.66 mL, 0.66 mmol, 1 M solution in diethyl ether) was added dropwise to a stirred solution of **2a** ^**Dipp**^ (0.401 g, 0.17 mmol) in dichloromethane (5 mL), and the mixture was stirred for 16 h. The volatiles were removed in vacuo, and the crude product was washed with hexane (10 mL) and recrystallized from a mixture of dichloromethane and hexane (1:1), yielding **[3a** ^**Dipp**^ **]­[HCl**_**2**_ **]** as colorless single crystals. Yield: 0.32 g, 77%. Mp. 162 °C. ESI-MS (*m*/*z* value): calcd. for C_56_H_81_N_4_B_8_^+^: 897.72002; found 897.71989. ^1^H NMR (25 °C, DCM-*d*_2_, 500 MHz): δ = −3.40 (s broad, 1H, B*H*B), 1.02 (d, ^3^ *J*(^1^H, ^1^H) = 6.9 Hz, 12H, CH­(C*H*_3_)_2_), 1.05 (d, ^3^ *J*(^1^H, ^1^H) = 7.0 Hz, 12H, CH­(C*H*_3_)_2_), 1.09 (d, ^3^ *J*(^1^H, ^1^H) = 6.9 Hz, 6H, CH­(C*H*_3_)_2_), 1.11 (d, ^3^ *J*(^1^H, ^1^H) = 6.9 Hz, 6H, CH­(C*H*_3_)_2_), 1.17 (s, 1H, BC*H*), 1.23 (d, ^3^ *J*(^1^H, ^1^H) = 6.7 Hz, 6H, CH­(C*H*_3_)_2_), 1.24 (d, ^3^ *J*(^1^H, ^1^H) = 6.7 Hz, 6H, CH­(C*H*_3_)_2_), 2.16–2.29 (m, 6H, C*H*(CH_3_)_2_), 2.32 (sept, ^3^ *J*(^1^H, ^1^H) = 6.8 Hz, 2H, C*H*(CH_3_)_2_), 3.88 (s, 1H, BC*H*), 7.29–7.35 (m, 8H, *m*-C_6_ *H*_3_), 7.41 (s, 2H, C*H*=C*H*), 7.53 (t, ^3^ *J*(^1^H, ^1^H) = 7.8 Hz, ^3^ *J*(^1^H, ^1^H) = 7.9 Hz, 4H, *p*-C_6_ *H*_3_), 7.60 (s, 2H, C*H*=C*H*) ppm. ^11^B NMR (25 °C, DCM-*d*_2_, 160.46 MHz) δ = −33.4 (s, 1B, B6), −29.2 (d broad, ^1^ *J*(^1^H, ^11^B) = 157.0 Hz, 2B, B1,3), −13.7 (d broad, ^1^ *J*(^1^H, ^11^B) = 135.6 Hz,1B, B10), −7.8 (d broad, ^1^ *J*(^1^H, ^11^B) = 137.4 Hz, 2B, B5,8), −6.1 (d broad, ^1^ *J*(^1^H, ^11^B) = 146.1 Hz, 1B, B2), 10.3 (s broad, 1B, B4) ppm. ^13^C­{^1^H} NMR (25 °C, DCM-*d*_2_, 125.76 MHz): δ = 21.9, 22.0, 22.4, 22.5, 25.7, 25.8, 26.1, 26.2 (CH­(*C*H_3_)_2_), 29.4, 29.4, 29.5, 29.6 (*C*H­(CH_3_)_2_), 44.4 (broad, B*C*H), 107.5 (broad, B*C*H), 124.4, 124.8, 125.1, 125.3 (*m*-*C*_6_H_3_), 125.6, 127.0 (H*C*=*C*H), 131.7 (*p*-*C*_6_H_3_), 132.2 (*ipso*-C_6_H_3_), 132.3 (*p*-*C*_6_H_3_), 133.6 (*ipso*-C_6_H_3_), 145.4, 145.5, 145.8, 146.2 (*o*-C_6_H_3_), 150.9, 157.3 (m, N*C*N) ppm.

### Synthesis of **[3a^Dipp^]­[H­(TFA)_2_]**

Trifluoroacetic acid (0.135 mL, 1.8 mmol) was added dropwise to a stirred solution of **2a** ^**Dipp**^ (0.405 g, 0.4 mmol) in dichloromethane (10 mL) at room temperature, and the mixture was stirred for 16 h. The volatiles were removed in vacuo, and the solid residue was washed with hexane (10 mL) and recrystallized from a mixture of dichloromethane and hexane (1:1), yielding **[3a** ^**Dipp**^ **]­[H­(TFA)**_**2**_ **]** as colorless single crystals. Yield: 0.33 g, 66%. Mp. 210–212 °C. ESI-MS (*m*/*z* value): calcd. for C_56_H_81_N_4_B_8_^+^: 897.72002; found 897.71983. ^1^H NMR (25 °C, DCM-*d*_2_, 500 MHz): δ = −3.38 (s broad, 1H, B*H*B), 1.04–1.13 (m, 36H, CH­(C*H*_3_)_2_), 1.19 (s, BC*H*), 1.23 (d, ^3^ *J*(^1^H, ^1^H) = 6.7 Hz, 6H, CH­(C*H*_3_)_2_), 1.26 (d, ^3^ *J*(^1^H, ^1^H) = 6.7 Hz, 6H, CH­(C*H*_3_)_2_), 2.15–5.22 (m, 6H, C*H*(CH_3_)_2_), 2.26 (sept, ^3^ *J*(^1^H, ^1^H) = 6.9 Hz, 2H, C*H*(CH_3_)_2_), 2.33 (sept, ^3^ *J*(^1^H, ^1^H) = 6.7 Hz, 2H, C*H*(CH_3_)_2_), 3.90 (s, 1H, BC*H*), 7.09 (s, 2H, C*H*=C*H*), 7.24–7.29 (m, 8H, *m*-C_6_ *H*_3_), 7.44 (s, 2H, C*H*=C*H*), 7.50–7.55 (m, 4H, *p*-C_6_ *H*_3_) ppm. ^11^B NMR (25 °C, DCM-*d*_2_, 160.46 MHz) δ = −33.3 (s, 1B, B6), −29.2 (d broad, ^1^ *J*(^1^H, ^11^B) = 153.5 Hz, 2B, B1,3), −13.7 (d broad, ^1^ *J*(^1^H, ^11^B) = 124.4 Hz, 1B, B10), −7.7 (d broad, ^1^ *J*(^1^H, ^11^B) = 130.5 Hz, 2B, B5,8), −6.1 (d broad, ^1^ *J*(^1^H, ^11^B) = 146.1 Hz, 1B, B2), 10.2 (s broad, 1B, B4) ppm. ^13^C­{^1^H} NMR (25 °C, DCM-*d*_2_, 125.76 MHz): δ = 22.0, 22.1, 22.4, 22.5, 25.6, 25.7, 21.1, 26.2 (CH­(*C*H_3_)_2_), 29.4, 29.5, 29.5, 29.6 (*C*H­(CH_3_)_2_), 44.4 (B*C*H), 107.5 (broad, B*C*H), 116.2 (q, ^1^ *J*(^19^F, ^13^C) = 288.4 Hz, *C*F_3_COO^–^), 124.4, 124.5, 125.2, 125.4 (*m*-*C*_6_H_3_), 125.6, 126.6 (sH*C*=*C*H), 131.7 (*p*-*C*_6_H_3_), 132.1 (*ipso*-C_6_H_3_), 132.4 (*p*-*C*_6_H_3_), 133.6 (*ipso*-C_6_H_3_), 145.4, 145.5, 145.8, 146.2 (*o*-C_6_H_3_), 151.4 (very broad, N*C*N), 159.9 (q, ^1^ *J*(^11^B, ^13^C) = 38.7 Hz, N*C*N) ppm.

### An Attempted Synthesis of **2b^Dipp^**

A solution of 4-(dimethylamino)­pyridine (0.018 g, 0.15 mmol) in THF (5 mL) was added dropwise to a stirred solution of **[3a** ^**Dipp**^ **]­[HCl**_**2**_ **]** (0.105 g, 0.12 mmol) in tetrahydrofuran (5 mL) at −78 °C, and the mixture was stirred for 16 h. The yellow reaction mixture was filtered, and the crude product was recrystallized from a mixture of tetrahydrofuran and hexane (1:1), yielding a mixture of single crystals: **2b** ^**Dipp**^ and **2a** ^**Dipp**^.

### Synthesis of **1^MIC^**

A solution of **:I^MIC^** (0.64 g, 1.3 mmol) in tetrahydrofuran (5 mL) was added dropwise to a solution of *o*-C_2_B_8_H_10_ (0.06 g, 0.6 mmol) in tetrahydrofuran (2 mL) at room temperature. The reaction mixture was stirred for 16 h. The red suspension was filtered, washed with toluene (20 mL) and hexane (20 mL), and dried in vacuo, yielding **1** ^**MIC**^ as an orange powder, 0.15 g, 25%. Mp. >320 °C. ESI-MS (*m*/*z* value): calcd. for C_72_H_99_N_4_B_8_^+^: 1107.86087; found 1107.86022 [Fragment: (M–H)^+^]. ^1^H NMR (25 °C, THF-D_8_, 500 MHz): δ = 0.83 (d, ^3^ *J*(^1^H, ^1^H) = 7.0 Hz, 6H, CH­(C*H*_3_)_2_), 0.85 (d, ^3^ *J*(^1^H, ^1^H) = 7.0 Hz, 6H, CH­(C*H*_3_)_2_), 0.95 (d, ^3^ *J*(^1^H, ^1^H) = 6.5 Hz, 6H, CH­(C*H*_3_)_2_), 0.99 (d, ^3^ *J*(^1^H, ^1^H) = 6.7 Hz, 6H, CH­(C*H*_3_)_2_), 1.03 (d, ^3^ *J*(^1^H, ^1^H) = 6.7 Hz, 6H, CH­(C*H*_3_)_2_), 1.14 (d, ^3^ *J*(^1^H, ^1^H) = 6.4 Hz, 6H, CH­(C*H*_3_)_2_), 1.21 (d, ^3^ *J*(^1^H, ^1^H) = 6.6 Hz, 12H, CH­(C*H*_3_)_2_), 1.26 (s, 2H, BC*H*), 1.93 (s, 12H, ArC*H*_3_), 2.37 (sept, ^3^ *J*(^1^H, ^1^H) = 6.6 Hz, 2H, C*H*(CH_3_)_2_), 2.59 (sept, ^3^ *J*(^1^H, ^1^H) = 6.4 Hz, 4H, C*H*(CH_3_)_2_), 2.69 (sept, ^3^ *J*(^1^H, ^1^H) = 6.6 Hz, 2H, C*H*(CH_3_)_2_), 6.43 (s, 4H, *o-*C_6_ *H*_3_Me_2_), 6.82 (s, 2H, *p-*C_6_ *H*_3_Me_2_), 7.08 (d, ^3^ *J*(^1^H, ^1^H) = 7.6 Hz, 2H, *m*-C_6_ *H*_3_), 7.14 (d, ^3^ *J*(^1^H, ^1^H) = 7.5 Hz, 2H, *m*-C_6_ *H*_3_), 7.22 (s, 2H, C*H*), 7.26 (d, ^3^ *J*(^1^H, ^1^H) = 7.8 Hz, 2H, *m*-C_6_ *H*_3_), 7.43 (t, ^3^ *J*(^1^H, ^1^H) = 8.4 Hz, 2H, *p*-C_6_ *H*_3_), 7.49 (t, ^3^ *J*(^1^H, ^1^H) = 7.6 Hz, 2H, *p*-C_6_ *H*_3_) ppm. ^11^B NMR (25 °C, THF-D_8_, 160.46 MHz) δ = −48.5 (d, ^1^ *J*(^1^H, ^11^B) = 144.2 Hz, 1B, B3), – 34.3 (d broad, ^1^ *J*(^1^H, ^11^B) = 101.8 Hz, 3B, B1,6,9), −6.7 (s broad, 2B, B7,8), −0.8 (s broad, 2B, B2,4) ppm. ^13^C­{^1^H} NMR (25 °C, THF-D_8_, 125.76 MHz): δ = 20.75 (Ar*C*H_3_), 22.7, 22.7, 23.0, 24.0, 25.6, 25.7, 25.8, 25.9 (CH­(*C*H_3_)_2_), 28.3 (B*C*H), 29.5, 29.7, 29.8 (*C*H­(CH_3_)_2_), 124.4, 124.7 (*m*-C_6_ *H*_3_), 125.1 (*m*-C_6_ *H*_3_), 125.4 (*m*-C_6_ *H*_3_), 125.5, 128.0 (*o-C*_6_H_3_), 130.4 (*p*-C_6_ *H*_3_), 131.4 (*p*-C_6_ *H*_3_), 132.2 (*p-C*_6_H_3_), 133.32 (*ipso*-C_6_H_3_), 133.8 (*ipso*-C_6_H_3_), 138.5 (*m*-C_6_H_3_), 142.2 (N*C*N), 145.6 (*o*-C_6_H_3_), 145.8 (*o*-C_6_H_3_), 146.2 (*o*-C_6_H_3_), 147.3 (*o*-C_6_H_3_), 158.9 (m, N*C*N) ppm.

### Synthesis of **2^MIC^**

A solution of **1** ^**MIC**^ (0.150 g, 0.1 mmol) in tetrahydrofuran (3 mL) was heated to 70 °C, and the mixture was stirred for 16 h. The orange solution was left to crystallize for 1 day at room temperature. The solution was decanted, and the crystals of **2** ^**MIC**^ were washed with tetrahydrofuran (5 mL). Yield: 0.13 g, 87%. Mp. >320 °C. ESI-MS (*m*/*z* value): calcd. for C_72_H_97_N_4_B_8_^+^: 1105.84522; found 1105.84645 [Fragment: (M–H)^+^]. ^1^H NMR (25 °C, THF-D_8_, 500 MHz): δ = 0.59 (d, ^3^ *J*(^1^H, ^1^H) = 6.7 Hz, 6H, CH­(C*H*_3_)_2_), 0.77 (d, ^3^ *J*(^1^H, ^1^H) = 6.7 Hz, 6H, CH­(C*H*_3_)_2_), 0.95 (d, ^3^ *J*(^1^H, ^1^H) = 6.4 Hz, 6H, CH­(C*H*_3_)_2_), 1.12 (d, ^3^ *J*(^1^H, ^1^H) = 6.7 Hz, 6H, CH­(C*H*_3_)_2_), 1.15 (d, ^3^ *J*(^1^H, ^1^H) = 6.8 Hz, 6H, CH­(C*H*_3_)_2_), 1.20 (d, ^3^ *J*(^1^H, ^1^H) = 6.4 Hz, 6H, CH­(C*H*_3_)_2_), 1.35 (d, ^3^ *J*(^1^H, ^1^H) = 6.7 Hz, 6H, CH­(C*H*_3_)_2_), 1.38 (d, ^3^ *J*(^1^H, ^1^H) = 6.5 Hz, 6H, CH­(C*H*_3_)_2_), 1.97 (s, 12H, ArC*H*_3_), 2.41 (sept, ^3^ *J*(^1^H, ^1^H) = 6.7 Hz, 2H, C*H*(CH_3_)_2_), 2.51 (sept, ^3^ *J*(^1^H, ^1^H) = 6.6 Hz, 2H, C*H*(CH_3_)_2_), 2.79 (sept, ^3^ *J*(^1^H, ^1^H) = 6.7 Hz, 2H, C*H*(CH_3_)_2_), 2.97 (sept, ^3^ *J*(^1^H, ^1^H) = 6.7 Hz, 2H, C*H*(CH_3_)_2_), 3.55 (s, 2H, BC*H*), 6.58 (s, 4H, *o-*C_6_ *H*_3_), 6.86 (s, 2H, *p-*C_6_ *H*_3_), 7.12 (d, ^3^ *J*(^1^H, ^1^H) = 7.6 Hz, 2H, *m*-C_6_ *H*_3_), 7.18 (d, ^3^ *J*(^1^H, ^1^H) = 7.6 Hz, 2H, *m*-C_6_ *H*_3_), 7.22 (s, 2H, C*H*), 7.26 (d, ^3^ *J*(^1^H, ^1^H) = 7.8 Hz, 2H, *m*-C_6_ *H*_3_), 7.43 (t, ^3^ *J*(^1^H, ^1^H) = 8.4 Hz, 2H, *p*-C_6_ *H*_3_), 7.49 (t, ^3^ *J*(^1^H, ^1^H) = 7.6 Hz, 2H, *p*-C_6_ *H*_3_) ppm. ^11^B NMR (25 °C, THF-D_8_, 160.46 MHz) δ = −29.5 (d, ^1^ *J*(^1^H, ^11^B) = 136.5 Hz, 2B, B1,3), −8.1 (s broad, 2B, B5,8), −6.5 (s broad, 2B, B7,10), −2.3 (s broad, 2B, B2,4) ppm. ^13^C­{^1^H} NMR (25 °C, THF-D_8_, 125.76 MHz): δ = 20.75 (Ar*C*H_3_), 22.7, 22.7, 23.0, 24.0, 25.6, 25.7, 25.8, 25.9 (CH­(*C*H_3_)_2_), 29.5, 29.7, 29.8 (*C*H­(CH_3_)_2_), 111.2 (B*C*H), 124.4, 124.7 (*m*-C_6_ *H*_3_), 125.1 (*m*-C_6_ *H*_3_), 125.4 (*m*-C_6_ *H*_3_), 125.5, 128.0 (*o-C*_6_H_3_), 130.4 (*p*-C_6_ *H*_3_), 131.4 (*p*-C_6_ *H*_3_), 132.2 (*p-C*_6_H_3_), 133.32 (*ipso*-C_6_H_3_), 133.8 (*ipso*-C_6_H_3_), 138.5 (*m*-C_6_H_3_), 142.2 (N*C*N), 145.6 (*o*-C_6_H_3_), 145.8 (*o*-C_6_H_3_), 146.2 (*o*-C_6_H_3_), 147.3 (*o*-C_6_H_3_) ppm.

### Synthesis of **1^*i*Pr^**

A solution of *o*-C_2_B_8_H_10_ (0.25 g, 2 mmol) in diethyl ether (2 mL) was added dropwise to a stirred solution of **:I^*i*Pr^** (0.73 g, 4.8 mmol) in diethyl ether (10 mL) at room temperature. The yellow reaction mixture was stirred for 16 h. The volatiles were removed in vacuo, and the solid residue was washed with diethyl ether (2 × 15 mL), yielding **1** ^***i*** **Pr**^ as a slightly yellow powder. Yield: 0.85 g, 96%. Mp. 119–121 °C. ESI-MS (*m*/*z* value): calcd. for C_20_H_43_N_4_B_8_^+^: 427.42267; found 427.42260 [Fragment: (M–H)^+^]. ^1^H NMR (25 °C, THF-D_8_, 500 MHz): δ = 1.39 (d, ^3^ *J*(^1^H, ^1^H) = 6.8 Hz, 24H, CH­(C*H*_3_)_2_), 5.79 (sept, ^3^ *J*(^1^H, ^1^H) = 6.8 Hz, 4H, CH­(C*H*_3_)_2_), 7.27 (s, 4H, C*H*=C*H*) ppm, BC*H* not observed. ^11^B NMR (25 °C, THF-D_8_, 160.46 MHz) δ = −49.7 (d, ^1^ *J*(^1^H, ^11^B) = 142.1 Hz, 1B, B3), −34.0 (d broad, ^1^ *J*(^1^H, ^11^B) = 125.9 Hz, 3B, B1,6,9), −6.4 (s broad, 2B, B7,8), −1.3 (s broad, 2B, B2,4) ppm. ^13^C­{^1^H} NMR (25 °C, THF-D_8_, 125.76 MHz): δ = 21.9, 23.4 (CH­(*C*H_3_)_2_), 49.6 (*C*H­(CH_3_)_2_), 116.7 (*C*H=*C*H), 165.3 (N*C*N) ppm, B*C*H not observed.

### Synthesis of **2a^*i*Pr^**

A solution of **1** ^***i*** **Pr**^ (0.34 g, 0.8 mmol) in toluene (5 mL) was heated to 90 °C, and the mixture was stirred for 3 h. The volatiles were removed in vacuo, and the solid residue was washed with diethyl ether (20 mL) and tetrahydrofuran (1 mL), yielding **2a** ^***i*** **Pr**^ as a white powder. Yield: 0.14 g, 41%. The diethyl ether and tetrahydrofuran solutions were combined and dried in vacuo. Crystallization from dichloromethane/hexane yielded a mixture of single crystals of **2a** ^***i*** **Pr**^ and **2a** ^***i*** **Pr**^ *. Mp. 220 °C. ESI-MS (*m*/*z* value): calcd. for C_20_H_41_N_4_B_8_^+^: 425.40702; found 425.40712 [Fragment: (M–H)^+^]. ^1^H NMR (25 °C, THF-D_8_, 500 MHz): δ = 1.18 (d, ^3^ *J*(^1^H, ^1^H) = 6.5 Hz, 12H, CH­(C*H*_3_)_2_), 1.19 (d, ^3^ *J*(^1^H, ^1^H) = 6.5 Hz, 12H, CH­(C*H*_3_)_2_), 4.47 (s, 2H, BC*H*), 5.79 (sept, ^3^ *J*(^1^H, ^1^H) = 6.6 Hz, 4H, C*H*(CH_3_)_2_), 7.26 (s, 4H, C*H*=C*H*) ppm. ^11^B NMR (25 °C, THF-D_8_, 160.46 MHz) δ = −32.1 (d, ^1^ *J*(^1^H, ^11^B) = 138.6 Hz, 2B, B1,3), −7.5 (d broad, ^1^ *J*(^1^H, ^11^B) = 123.2 Hz, 2B, B5,8), −6.5 (s broad, 2B, B7,10), −4.9 (s broad, 2B, B2,4) ppm. ^13^C­{^1^H} NMR (25 °C, THF-D_8_, 125.76 MHz): δ = 22.9, 23.4 (CH­(*C*H_3_)_2_), 49.6 (*C*H­(CH_3_)_2_), 116.7 (*C*H=*C*H), 118.1 (broad, B*C*H), 164.0 (broad, N*C*N) ppm.

### Synthesis of **2b^*i*Pr^**

A solution of **1** ^***i*** **Pr**^ (0.368 g, 1 mmol) in tetrahydrofuran (10 mL) was left to crystallize for 4 days at room temperature. The colorless single crystals of **2b** ^***i*** **Pr**^ were decanted and washed with toluene (10 mL). Yield: 0.15 g, 41%. Mp. 191–193 °C. ESI-MS (*m*/*z* value): calcd. for C_20_H_41_N_4_B_8_^+^: 425.40702; found 425.40705 [Fragment: (M–H)^+^]. ^1^H NMR (25 °C, DCM-*d*_2_, 500 MHz): δ = 1.27 (s broad, 12H, CH­(C*H*_3_)_2_), 1.32 (s broad, 12H, CH­(C*H*_3_)_2_), 5.00 (s, BC*H*), 5.51 (s broad, ^3^ *J*(^1^H, ^1^H) = 6.6 Hz, 4H, C*H*(CH_3_)_2_), 7.08 (s, 4H, C*H*=C*H*) ppm. ^11^B NMR (25 °C, DCM-*d*_2_, 160.46 MHz) δ = −29.9 (d, ^1^ *J*(^1^H, ^11^B) = 146.3 Hz, 2B, B1,2), −11.7 (s, 2B, B7,8), −7.7 (m, 2B, B5,10), 2.8 (d broad, ^1^ *J*(^1^H, ^11^B) = 103.3 Hz, 2B, B2, 4) ppm. ^13^C­{^1^H} NMR (25 °C, DCM-*d*_2_, 125.76 MHz): δ = 24.4 (broad, CH­(*C*H_3_)_2_), 50.3 (*C*H­(CH_3_)_2_), 117.6 (*C*H=*C*H), 118.3 (broad, B*C*H), 160.2 (broad, N*C*N) ppm.

### Synthesis of **2c^*i*Pr^**

A solution of **1** ^***i*** **Pr**^ (0.422 g, 1 mmol) in acetonitrile (20 mL) was heated to 110 °C, and the mixture was stirred for 8 h. The orange reaction mixture was filtered, and the solution was allowed to crystallize overnight. The colorless single crystals of **2c** ^***i*** **Pr**^ were decanted and dried in vacuo. Yield: 0.31 g, 67%. Mp. 256–257 °C. ESI-MS (*m*/*z* value): calcd. for C_22_H_44_N_5_B_8_^+^: 466.43357; found 466.43352 [Fragment: (M–H)^+^]. ^1^H NMR (25 °C, THF-D_8_, 500 MHz): δ = 1.31–1.34 (m, 15H, CH­(C*H*_3_)_2_), 1.36 (s broad, 1H, BC*H*), 1.38 (d, ^3^ *J*(^1^H, ^1^H) = 6.6 Hz, 3H, CH­(C*H*_3_)_2_), 1.43 (d, ^3^ *J*(^1^H, ^1^H) = 7.0 Hz, 3H, CH­(C*H*_3_)_2_), 1.48 (d, ^3^ *J*(^1^H, ^1^H) = 6.6 Hz, 3H, CH­(C*H*_3_)_2_), 2.06 (s, 3H, C*H*_3_), 2.08 (s broad, 1H, BC*H*C=N), 4.94 (sept, ^3^ *J*(^1^H, ^1^H) = 6.9 Hz, 1H, C*H*(CH_3_)_2_), 5.34 (sept, ^3^ *J*(^1^H, ^1^H) = 6.6 Hz, 2H, C*H*(CH_3_)_2_), 6.58 (sept, ^3^ *J*(^1^H, ^1^H) = 6.8 Hz, 1H, C*H*(CH_3_)_2_), 7.28 (s, 3H, C*H*=C*H*), 7.29 (s, 1H, C*H*=C*H*) ppm. ^11^B NMR (25 °C, THF-D_8_, 160.46 MHz) δ = −31.9 (d, ^1^ *J*(^1^H, ^11^B) = 140.2 Hz, 1B, B3), −30.5 (d, ^1^ *J*(^1^H, ^11^B) = 143.8 Hz, 1B, B1), −19.5 (s, 1B, B9), −11.3 (s broad, 1B, B10), −10.4 (s broad, 1B, B5), −7.5 (s broad, 1B, B7), −5.0 (s broad, 1B, B4), −1.5 (d broad, ^1^ *J*(^1^H, ^11^B) = 117.3 Hz 1B, B2) ppm. ^13^C­{^1^H} NMR (25 °C, THF-D_8_, 125.76 MHz): δ = 22.1, 23.3, 23.9, 24.9 (CH­(*C*H_3_)_2_), 30.0 (*C*H_3_), 36.8 (B*C*HC=N), 46.9 (broad, B*C*H), 48.8, 49.9, 50.5 (*C*H­(CH_3_)_2_), 116.2, 116.7, 117.3 (*C*H=*C*H), 160.7 (*C*=N), 163.0, 167.2 (broad, N*C*N) ppm.

### Synthesis of **[3a^*i*Pr^]­Cl**

A solution of hydrogen chloride (1.44 mL, 1.44 mmol, 1 M solution in diethyl ether) was added dropwise to a stirred solution of **2a** ^***i*** **Pr**^ (0.150 g, 0.35 mmol) in dichloromethane (2 mL), and the mixture was stirred for 5 min. The resulting colorless solution was left to crystallize overnight. The colorless single crystals of **[3a** ^***i*** **Pr**^ **]­Cl** were decanted and washed with dichloromethane (1 mL). Yield: 0.08 g, 49%. Mp. 160 °C. ESI-MS (*m*/*z* value): calcd. for C_20_H_42_ClN_4_B_8_^+^: 481.34999; found 481.35012 [Fragment: (M–Na)^+^]. ^1^H NMR (25 °C, DCM-*d*_2_, 500 MHz): δ = −1.64 (s broad, 1H, B*H*B), −0.12 (m broad, 1H, BC*H*_2_), 0.17 (m broad, 1H, BC*H*_2_), 1.43 (d, ^3^ *J*(^1^H, ^1^H) = 6.1 Hz, 6H, CH­(C*H*_3_)_2_), 1.48–1.53 (m, 15H, CH­(C*H*_3_)_2_), 1.60 (d, ^3^ *J*(^1^H, ^1^H) = 6.7 Hz, 3H, CH­(C*H*_3_)_2_), 2.32 (m broad, 1H, BC*H*_*X*_), 2.64 (m broad, 1H, BC*H*_*X*_), 5.56 (sept, ^3^ *J*(^1^H, ^1^H) = 6.4 Hz, 1H, C*H*(CH_3_)_2_), 5.81 (sept, ^3^ *J*(^1^H, ^1^H) = 6.4 Hz, 1H, C*H*(CH_3_)_2_), 5.94 (sept, ^3^ *J*(^1^H, ^1^H) = 6.6 Hz, 2H, C*H*(CH_3_)_2_), 7.14 (s, 1H, C*H*=C*H*), 7.23 (s, 1H, C*H*=C*H*), 7.33 (s, 2H, C*H*=C*H*) ppm. ^11^B NMR (25 °C, DCM-*d*_2_, 160.46 MHz) δ = ^11^B NMR (25 °C, DCM-*d*_2_, 160.46 MHz) δ = −48.5 (d, ^1^ *J*(^1^H, ^11^B) = 147.2 Hz, 1B, B3), −30.1 (d, ^1^ *J*(^1^H, ^11^B) = 141.4 Hz, 1B, B1), −25.1 (s broad, 1B, B5), −21.7 (s broad, 1B, B10), −19.7 (s broad, 1B, B7), −6.8 (d, ^1^ *J*(^1^H, ^11^B) = 128.8 Hz, 1B, B8), −2.4 (s broad, 2B, B2,4) ppm. ^13^C­{^1^H} NMR (25 °C, DCM-*d*_2_, 125.76 MHz): δ = −3.0, 4.0 (B*C*H), 22.8, 23.0, 23.4, 24.5 (CH­(*C*H_3_)_2_), 49.8, 51.5, 51.7 (*C*H­(CH_3_)_2_), 117.0, 118.0, 119.2 (*C*H=*C*H), 148.0, 154.9 (broad, N*C*N) ppm.

### Synthesis of **[3b^*i*Pr^]­Cl**

A solution of hydrogen chloride (1.23 mL, 1.2 mmol 1 M solution in diethyl ether) was added dropwise to a solution of **2b** ^***i*** **Pr**^ (0.13 g, 0.28 mmol) in dichloromethane (10 mL) at −78 °C and stirred for 5 min. The reaction mixture was left to crystallize for 1 day at room temperature. The colorless single crystals of **[3b** ^***i*** **Pr**^ **]­Cl** were decanted and washed with dichloromethane (1 mL). Yield: 0.09 g, 65%. Mp. 162 °C. ESI-MS (*m*/*z* value): calcd. for C_20_H_42_ClN_4_B_8_^+^: 481.34999; found 481.34989 [Fragment: (M–Na)^+^]. ^1^H NMR (25 °C, CD_3_OD, 500 MHz): δ = 0.32 (m, 2H, BC*H*_2_), 0.43 (m, 1H, BC*H*_*X*_), 0.92 (m, 1H, BC*H*_*X*_), 1.35 (d, 12H, ^1^ *J*(^1^H, ^11^B) = 7.8 Hz, CH­(C*H*_3_)_2_), 1.40 (d, 12H, ^1^ *J*(^1^H, ^11^B) = 7.8 Hz, CH­(C*H*_3_)_2_), 5.25 (sept, ^3^ *J*(^1^H, ^1^H) = 6.5 Hz, 4H, C*H*(CH_3_)_2_), 7.70 (s, 4H, C*H*=C*H*) ppm. ^11^B NMR (25 °C, CD_3_OD, 160.46 MHz) δ = −46.0 (d, ^1^ *J*(^1^H, ^11^B) = 137.8 Hz, 1B, B3), −29.4 (d, ^1^ *J*(^1^H, ^11^B) = 124.4 Hz, 1B, B1), −19.9 (s broad, 1B, B10), −16.2 (s broad, 1B, B5), −7.0 (s broad, 2B, B7,8), −2.2 (s broad, 2B, B2,4) ppm. ^13^C­{^1^H} NMR (25 °C, CD_3_OD, 125.76 MHz): δ = 0.3, 5.8 (B*C*H), 23.1, 24.1, 24.3, 25.1 (broad, CH­(*C*H_3_)_2_), 52.2 (broad, *C*H­(CH_3_)_2_), 120.8 (*C*H=*C*H), 155.6 (broad, N*C*N) ppm.

### Synthesis of **[3c^*i*Pr^]­[HCl_2_]**

A solution of hydrogen chloride (1.7 mL, 1.7 mmol 1 M solution in diethyl ether) was added dropwise to a solution of **2c** ^***i*** **Pr**^ (0.2 g, 0.43 mmol) in dichloromethane (1 mL) at −78 °C and stirred for 5 min. The reaction mixture was left to crystallize for 1 day at room temperature. The colorless single crystals of **[3c** ^***i*** **Pr**^ **]­[HCl**_**2**_ **]** were decanted and washed with dichloromethane (1 mL). Yield: 0.13 g, 50%. Mp. 162 °C. ESI-MS (*m*/*z* value): calcd. for C_22_H_44_N_5_B_8_^+^: 466.43357; found 466.43362. ^1^H NMR (25 °C, DCM-D_2_, 500 MHz): δ = 1.40 (d, ^3^ *J*(^1^H, ^1^H) = 6.8 Hz, 6H, CH­(C*H*_3_)_2_), 1.42–1.44 (d+d, ^3^ *J*(^1^H, ^1^H) = 6.6 Hz, ^3^ *J*(^1^H, ^1^H) = 6.6 Hz, 6 + 3H, CH­(C*H*_3_)_2_), 1.47 (d, ^3^ *J*(^1^H, ^1^H) = 6.8 Hz, 3H, CH­(C*H*_3_)_2_), 1.51 (d, ^3^ *J*(^1^H, ^1^H) = 6.6 Hz, 3H, CH­(C*H*_3_)_2_), 1.70 (d, ^3^ *J*(^1^H, ^1^H) = 6.7 Hz, 3H, CH­(C*H*_3_)_2_), 1.97 (s broad, 1H, BC*H*), 2.37 (s broad, 1H, BC*H*), 2.50 (s, 3H, C*H*_3_), 4.69 (sept, ^3^ *J*(^1^H, ^1^H) = 6.9 Hz, 2H, C*H*(CH_3_)_2_), 5.00 (s broad, 1H, C*H*(CH_3_)_2_), 6.21 (sept, ^3^ *J*(^1^H, ^1^H) = 6.7 Hz, 2H, C*H*(CH_3_)_2_), 7.11 (s, 2H, C*H*=C*H*), 7.19 (d, ^3^ *J*(^1^H, ^1^H) = 2.1 Hz, 1H, CH=C*H*), 7.21 (d, ^3^ *J*(^1^H, ^1^H) = 2.1 Hz, 1H, CH=C*H*), 10.91 (s, 1H, C=N*H*), 11.73 (s, 1H, HCl_2_^–^) ppm. ^11^B NMR (25 °C, DCM-*d*_2_, 160.46 MHz) δ = −25.9–(−25.4) (m, 2B, B1,3), −19.9 (s, 2B, B5,9), −6.4 (d broad, ^1^ *J*(^1^H, ^11^B) = 119.5 Hz, 1B, B10), −0.9 (s broad, 2B, B4,7), 3.5 (s broad, 1B, B2) ppm. ^13^C­{^1^H} NMR (25 °C, DCM-*d*_2_, 125.76 MHz): δ = 21.7, 23.0, 23.2, 23.6, 23.7, 24.4 (s, CH­(*C*H_3_)_2_), 25.7 (*C*H_3_), 37.8 (B*C*HC=N), 45.3 (B*C*H), 49.4, 50.2, 50.9 (*C*H­(CH_3_)_2_), 117.4, 117.5, 117.6 (*C*H=*C*H), 165.1 (broad, N*C*N) 179.9 (*C*=N).

## Supplementary Material





## Data Availability

The raw NMR data have been deposited to FigShare 10.6084/m9.figshare.30932633.

## Notes

The optimized structures are available in the XYZ file (XYZ)
Characterization data of the compounds prepared and computational details (PDF)
