# Supplementary material for: Electron Pool Enrichment of Polyhedral Carboranes: A Key to Isomerization and Nucleophilicity
Source: Inorg Chem. 2026 Mar 12;65(11):6101–10. doi: 10.1021/acs.inorgchem.5c06016 (PMC13014455; doi:10.1021/acs.inorgchem.5c06016)
Supplement: Supplementary file 2 [file ic5c06016_si_002.pdf]

Supporting Information for:

Electron Pool Enrichment of Polyhedral Carboranes: A Key to Isomerization and Nucleophilicity

Vlastimil Němec,[a] Josef Holub,[b] Maksim A. Samsonov,[a] Zdeňka Růžičková,[a] Josef Cvačka,[c] Jan Vrána*,[a] Aleš Růžička[a]

[a.] *Department of General and Inorganic Chemistry, Faculty of Chemical Technology, University of Pardubice, Studentská 573, 532 10 Pardubice, Czech Republic*

[b.] *Institute of Inorganic Chemistry, Czech Academy of Sciences, 250 68 Řež, Czech Republic*

[c.] *Institute of Organic Chemistry and Biochemistry of the Czech Academy of Sciences, Flemingovo náměstí 542/2, 166 10 Praha 6, Czech Republic*

email: jan.vrana@upce.cz

Table of Contents:

1. Characterization of prepared compounds S3

2. NMR Data S46

3. Crystallographic Data S49

4. Theoretical Investigations S63

5. References S65

Characterization of prepared compounds

Spectroscopic characterization of 2a^{Dipp}.

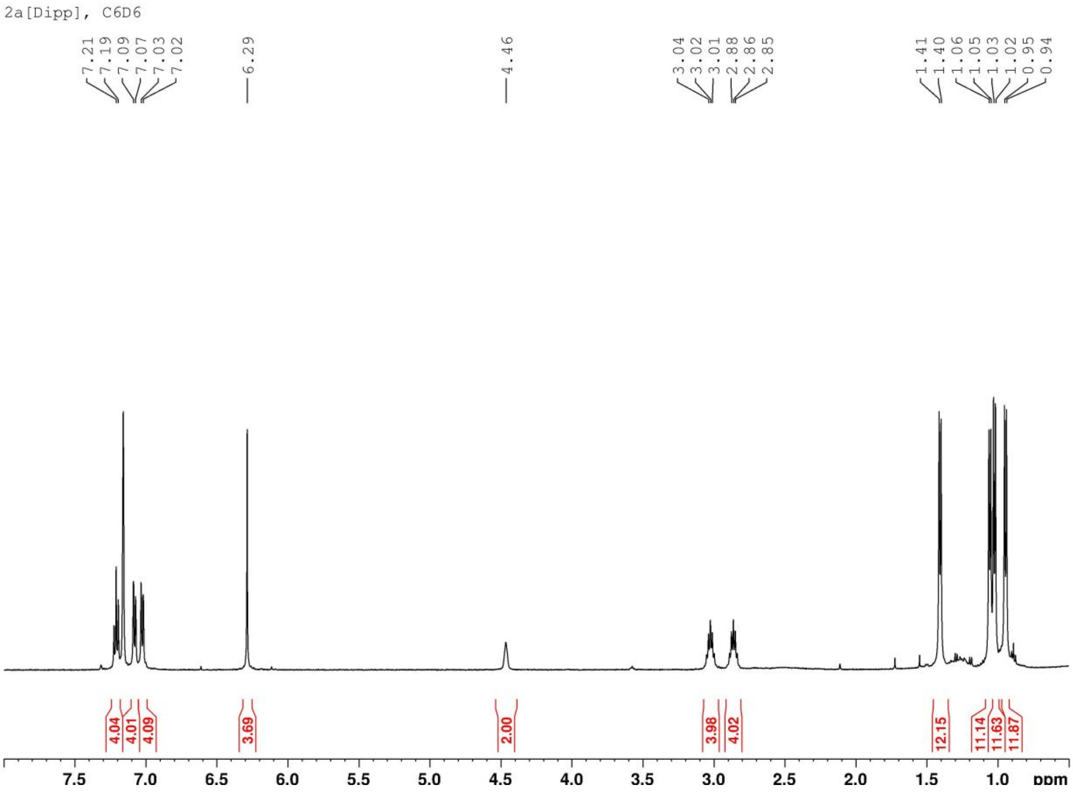

Figure S1. The ^{1}H NMR spectrum of **2a^{Dipp}**.

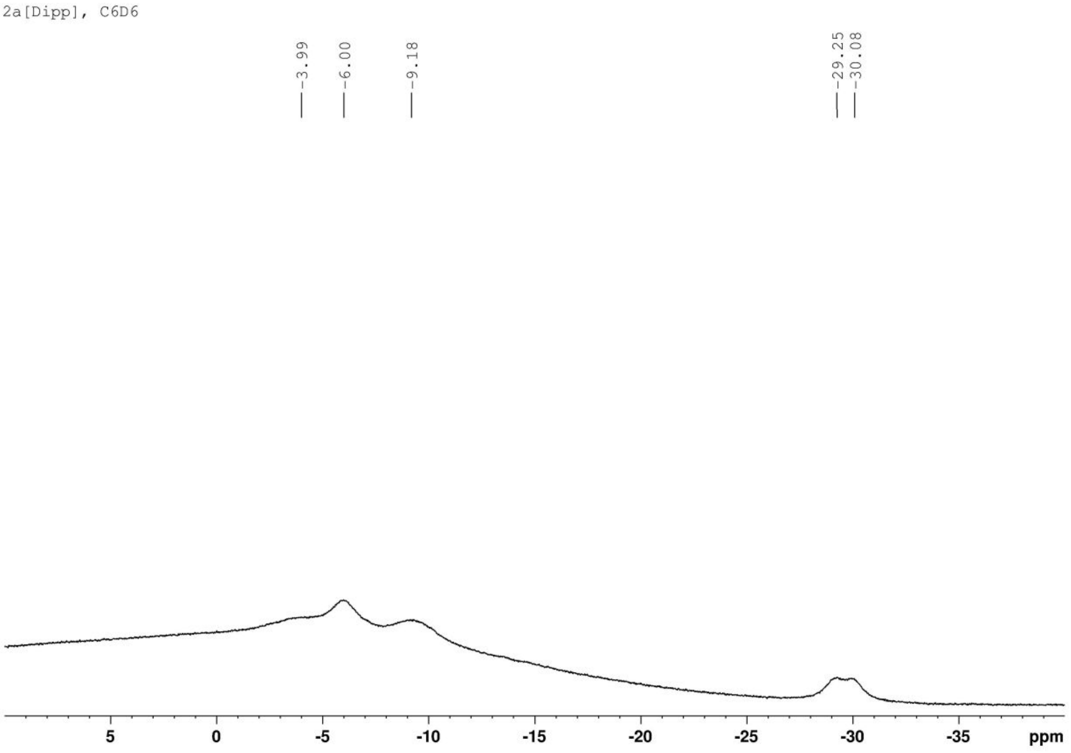

Figure S2. The ^{11}B NMR spectrum of **2a^{Dipp}**.

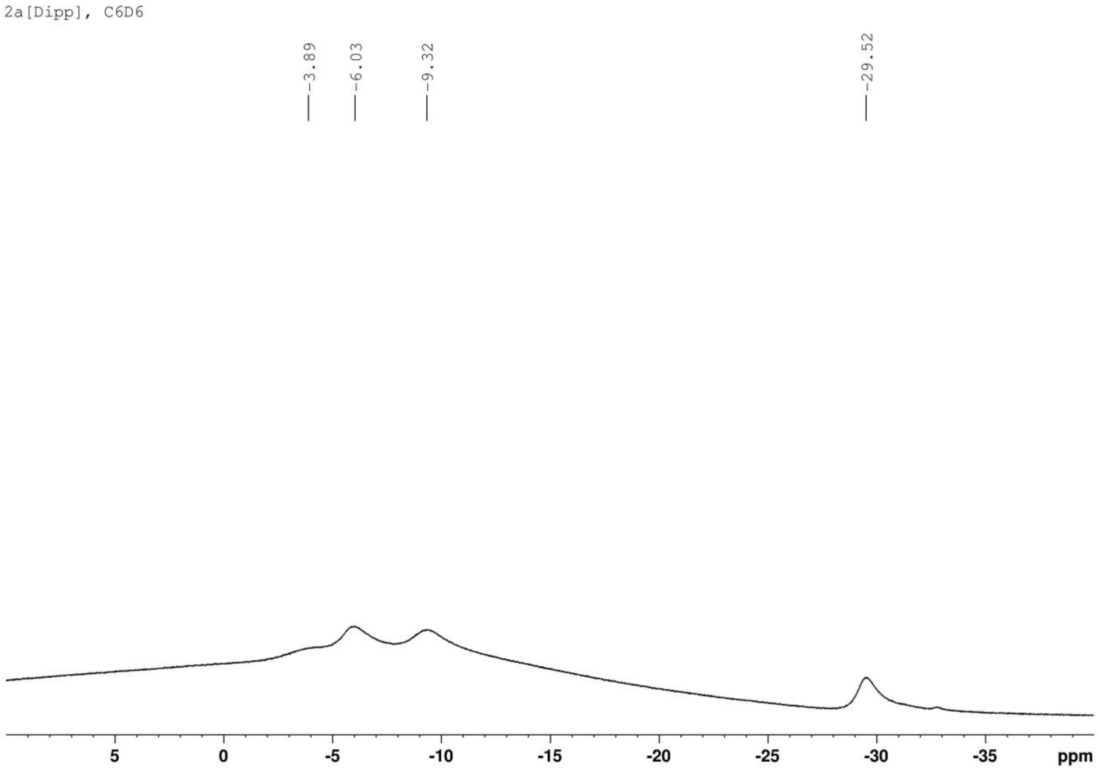

Figure S3. The $^{11}B\{^1H\}$ NMR spectrum of **2a^{Dipp}**.

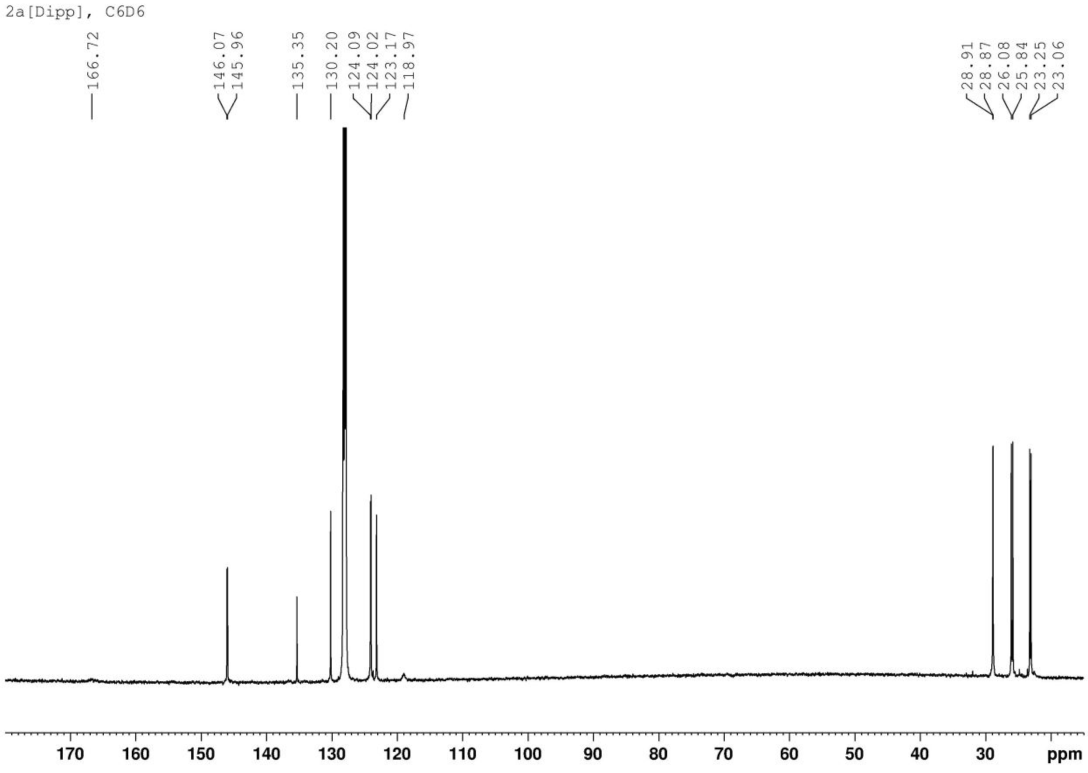

Figure S4. The $^{13}C\{^1H\}$ NMR spectrum of **2a^{Dipp}**.

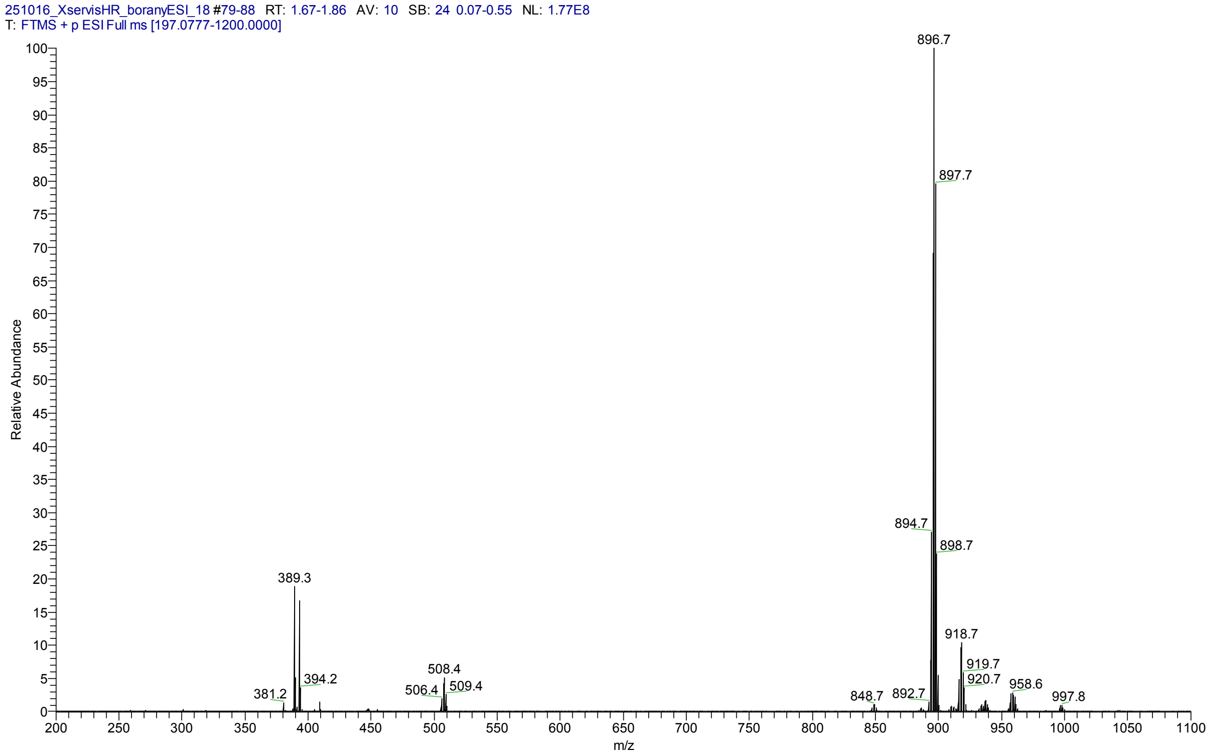

Figure S5. Mass spectrum of positively charged ions (ESI+, Orbitrap) for **2a$^{\mathbf{Dipp}}$**.

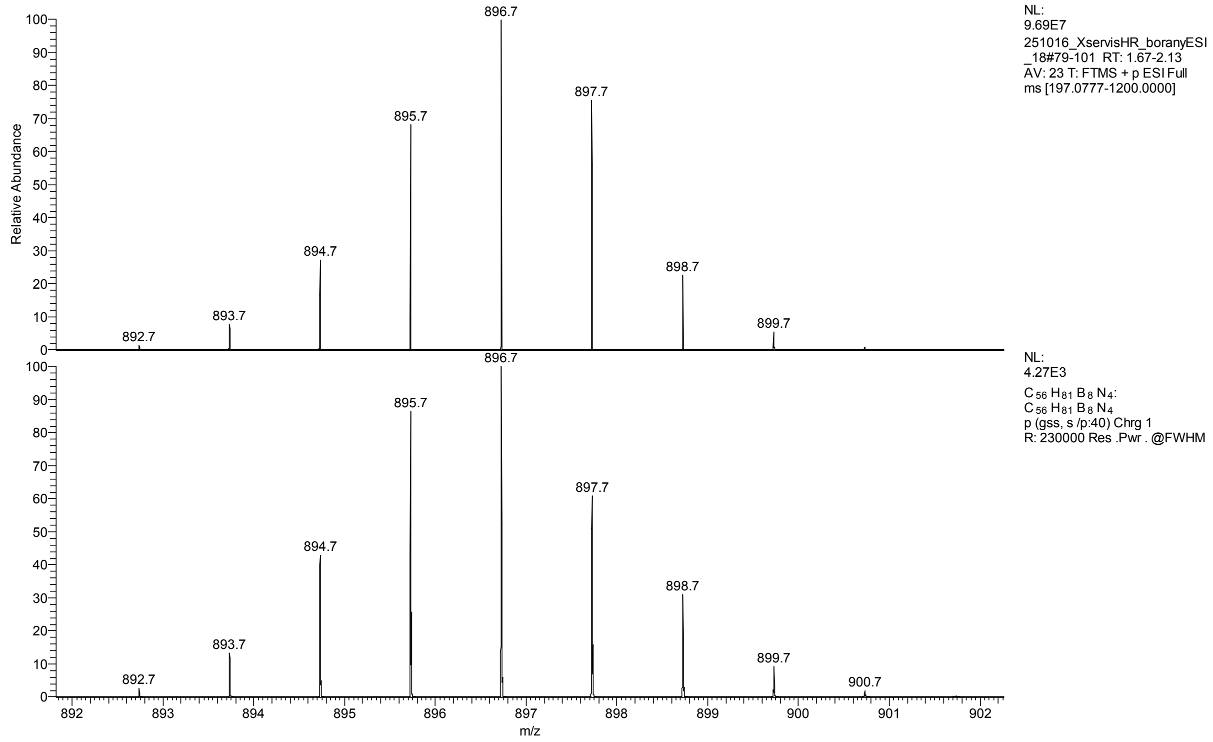

Figure S6. Spectrum of positively charged ions (ESI+, Orbitrap @ R=500,000) for **2a$^{\mathbf{Dipp}}$** enlarged in the protonated molecule region (top) and simulated spectrum (bottom).

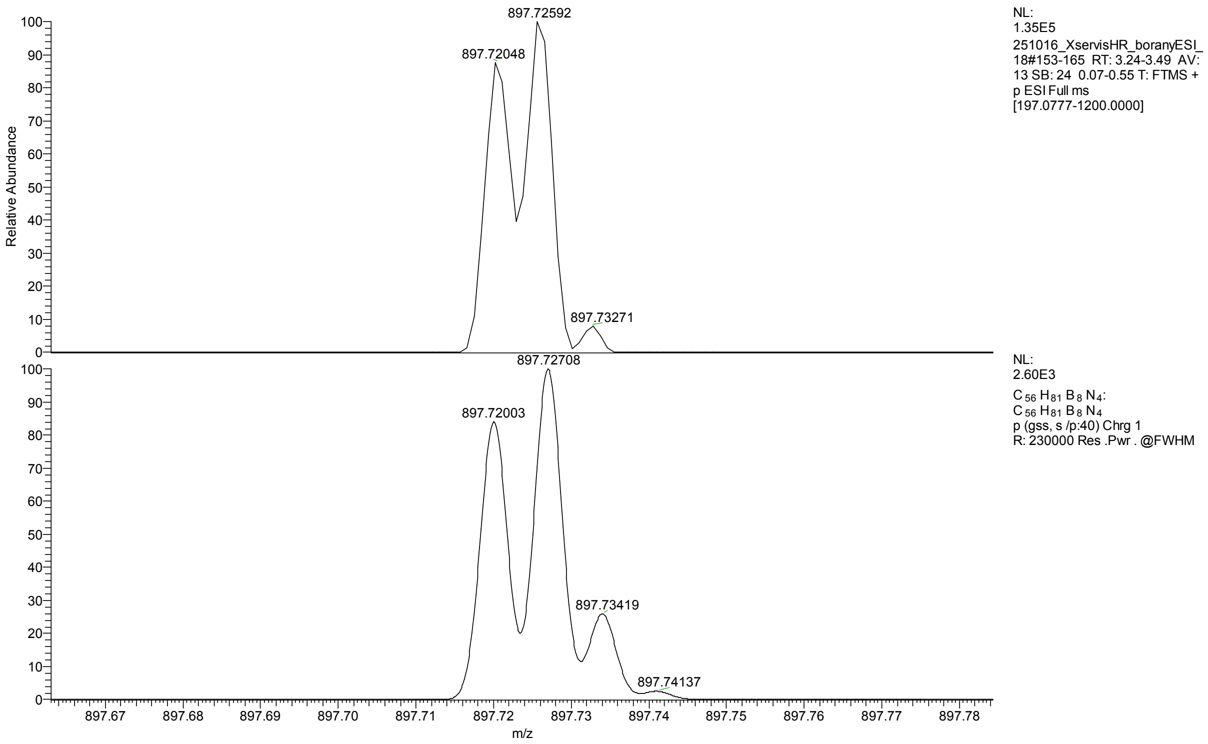

Figure S7. Spectrum of positively charged ions (ESI+, Orbitrap @ R=500,000) for **2a^Dipp^** enlarged in the monoisotopic peak region (top) and simulated spectrum (bottom). Theoretical mass for $C_{56}H_{81}N_4B_8^+$: *m/z* 897.72002; experimental mass: *m/z* 897.72048; mass error 0.51 ppm.

Spectroscopic characterization of [3a^Dipp^][HCl$_2$].

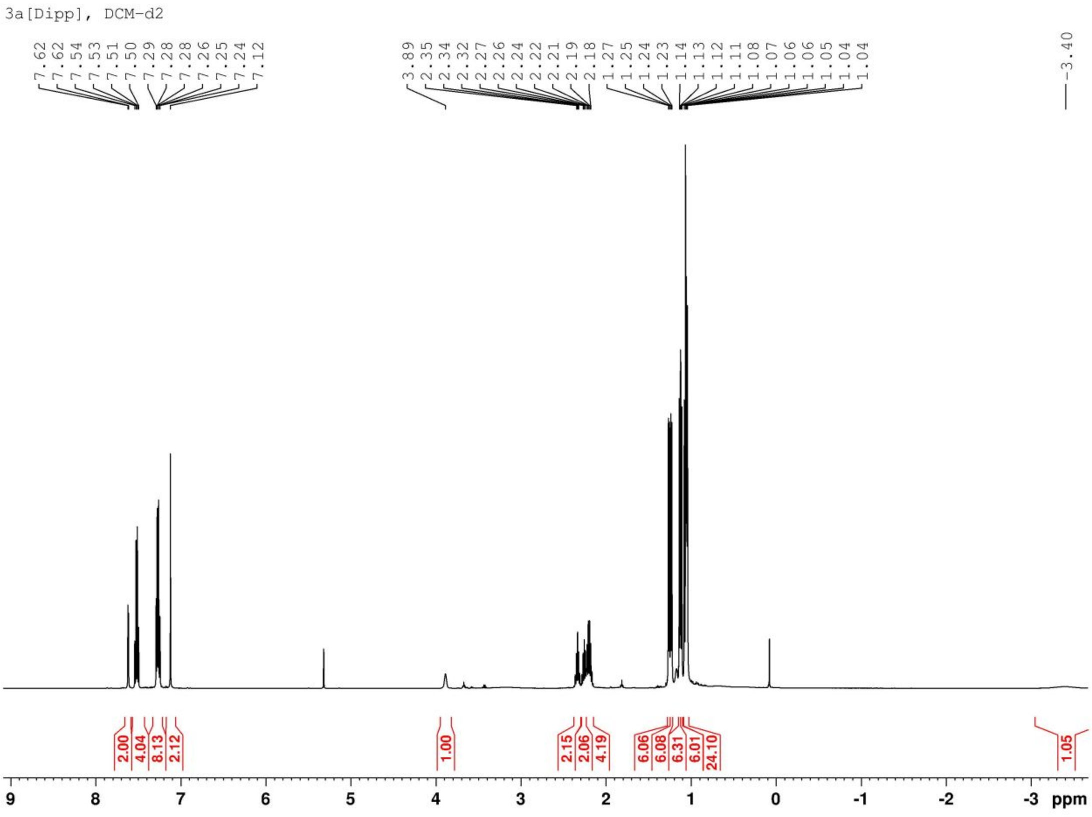

Figure S8. The ^{1}H NMR spectrum of **[3a^Dipp^][HCl$_2$]**.

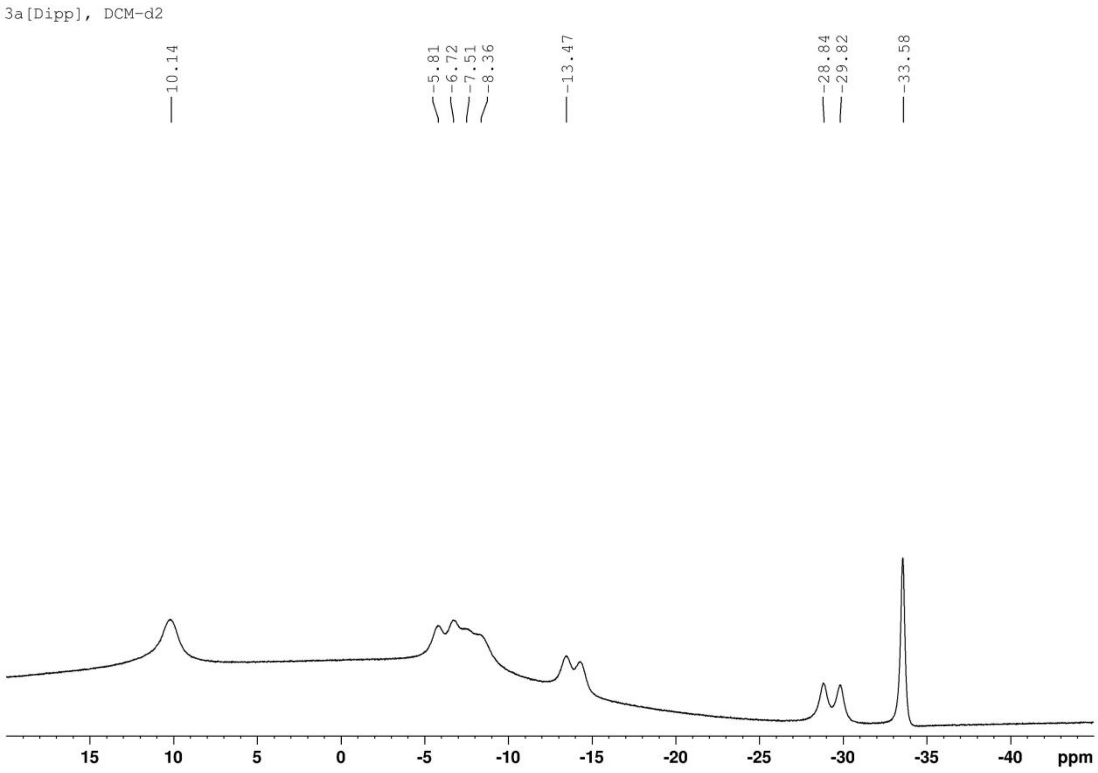

Figure S9. The ^{11}B NMR spectrum of **[3a^{Dipp}][HCl$_2$]**.

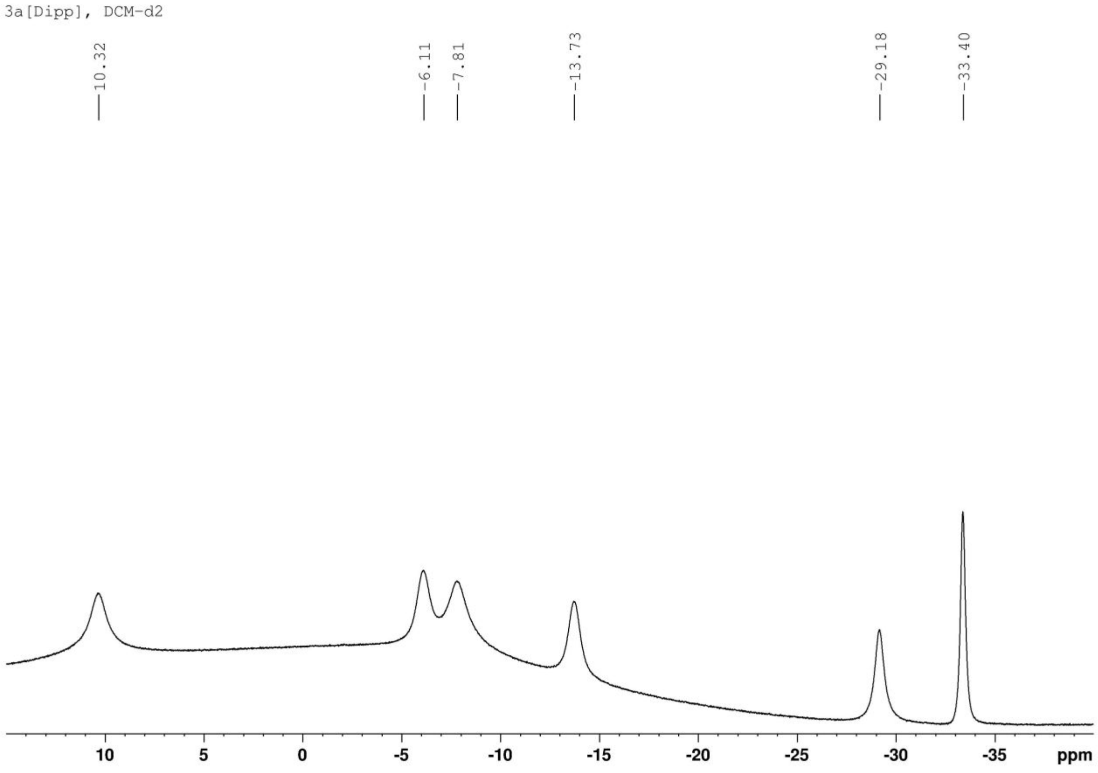

Figure S10. The $^{11}B\{^1H\}$ NMR spectrum of **[3a^{Dipp}][HCl$_2$]**.

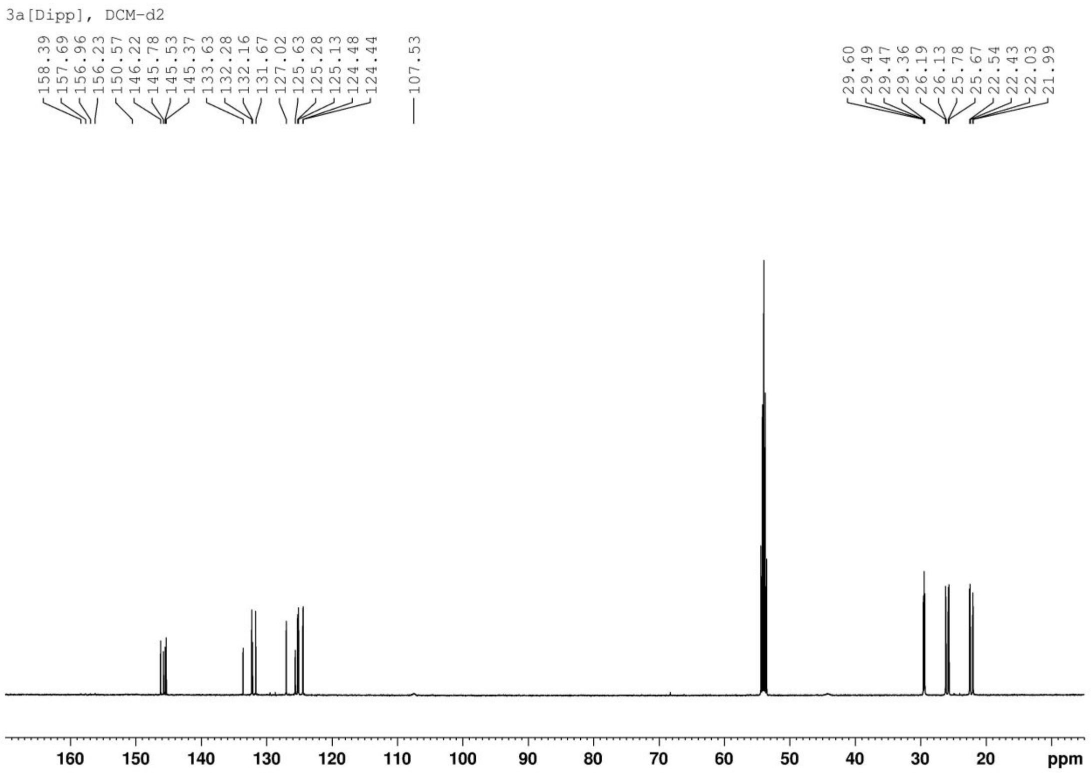

Figure S11. The $^{13}C\{^{1}H\}$ NMR spectrum of **[3a^{Dipp}][HCl$_2$]**.

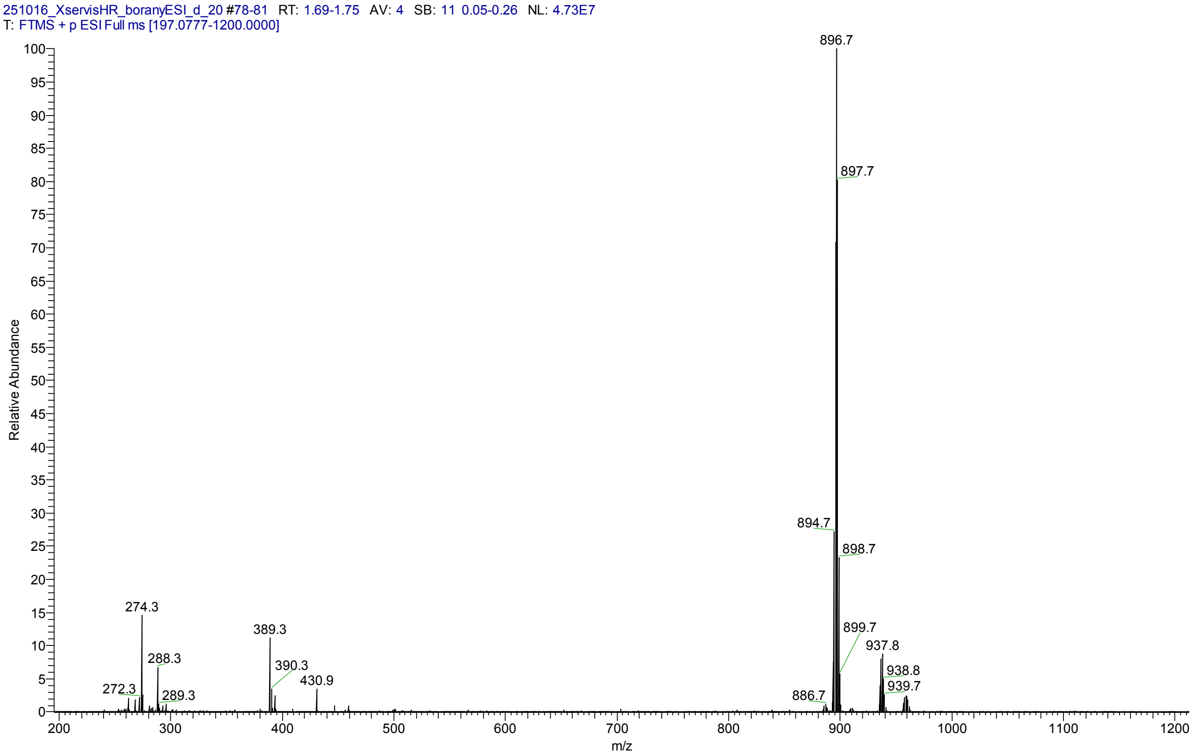

Figure S12. Mass spectrum of positively charged ions (ESI+, Orbitrap) for **[3a^{Dipp}][HCl$_2$]**.

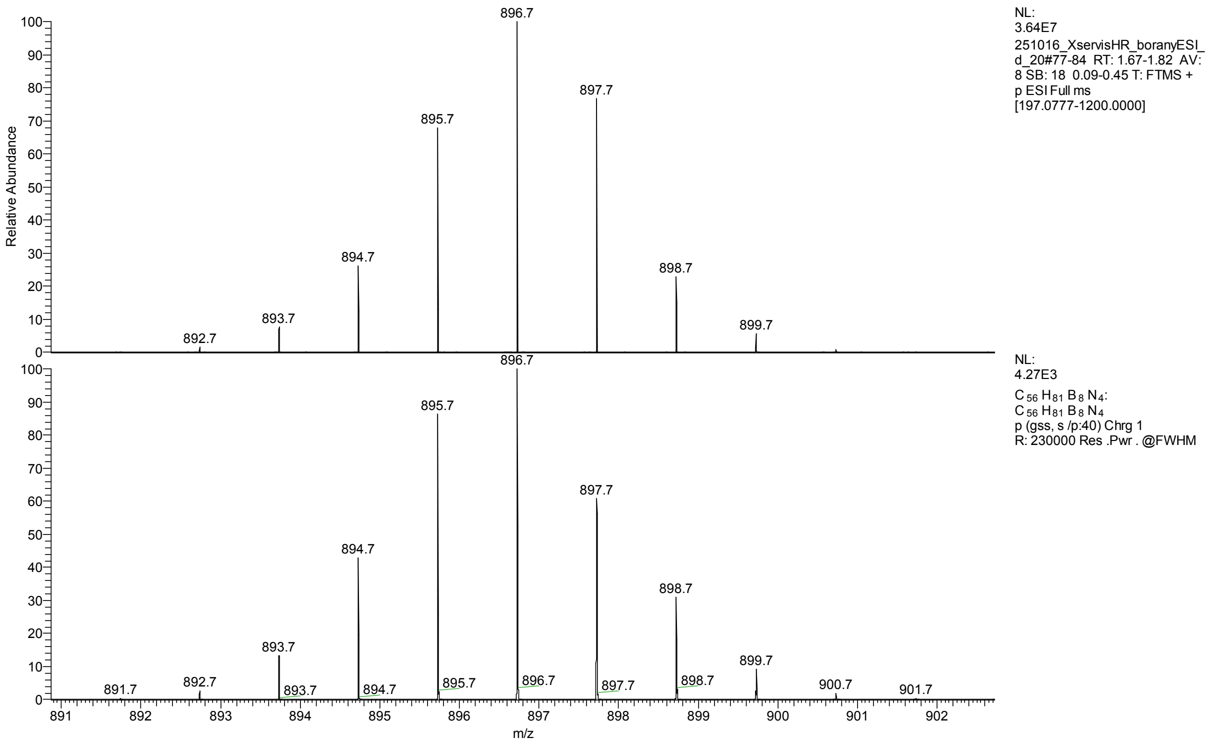

Figure S13. Spectrum of positively charged ions (ESI+, Orbitrap @ R=500,000) for **[3a^{Dipp}][HCl$_2$]** enlarged in the protonated molecule region (top) and simulated spectrum (bottom).

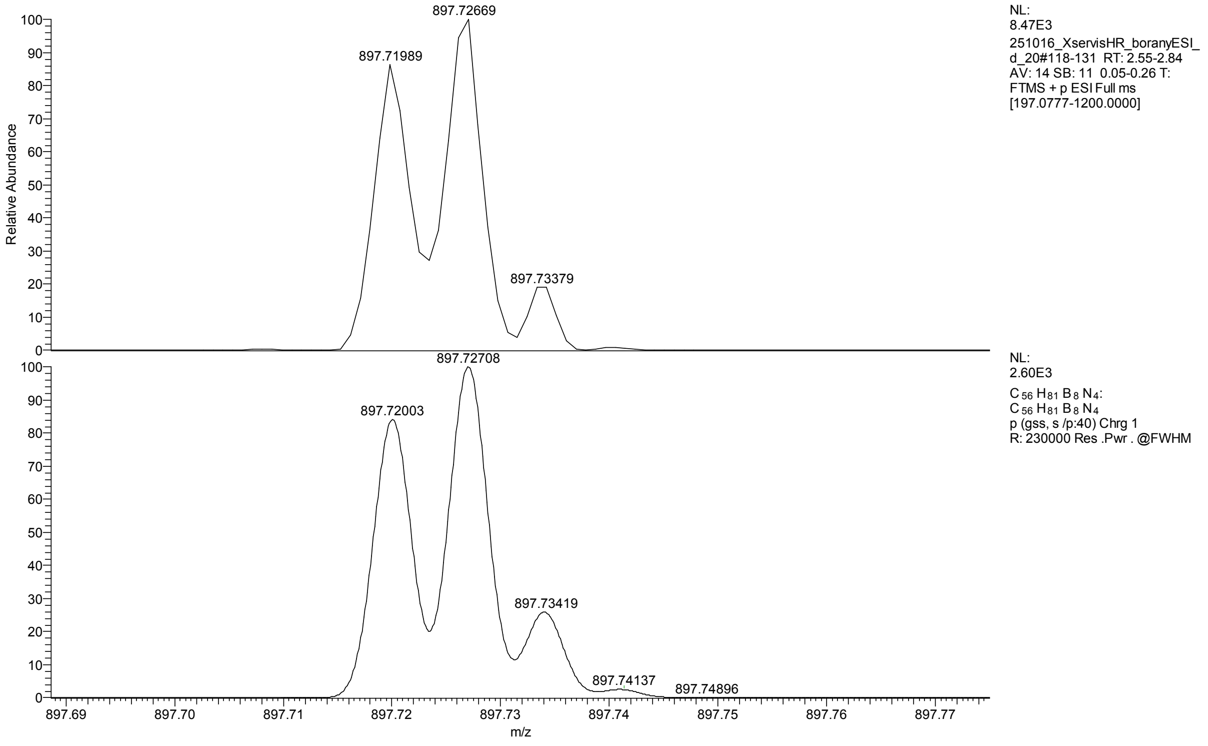

Figure S14. Spectrum of positively charged ions (ESI+, Orbitrap @ R=500,000) for **[3a^{Dipp}][HCl$_2$]** enlarged in the monoisotopic peak region (top) and simulated spectrum (bottom). Theoretical mass for $C_{56}H_{81}N_4B_8^+$: *m/z* 897.72002; experimental mass: *m/z* 897.71989; mass error -0.14 ppm.

Spectroscopic characterization of [3a^{Dipp}][H(TFA)$_2$].

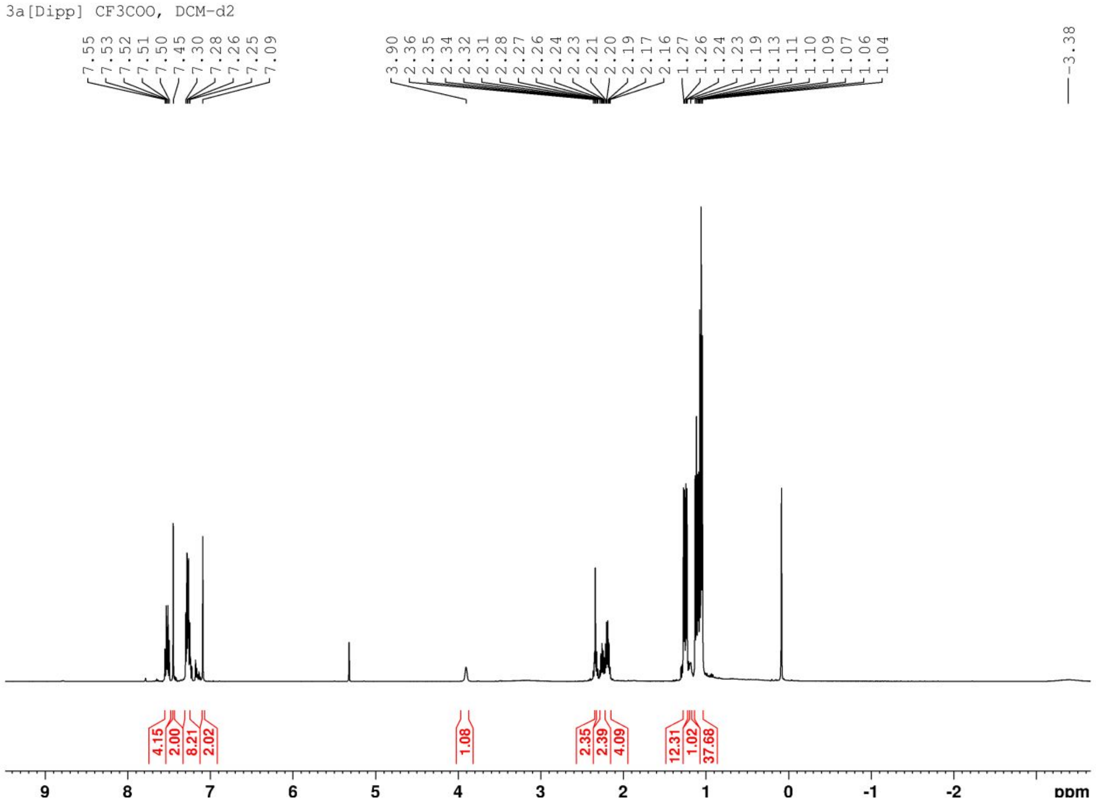

Figure S15. The ^{1}H NMR spectrum of **[3a^{Dipp}][H(TFA)$_2$]**.

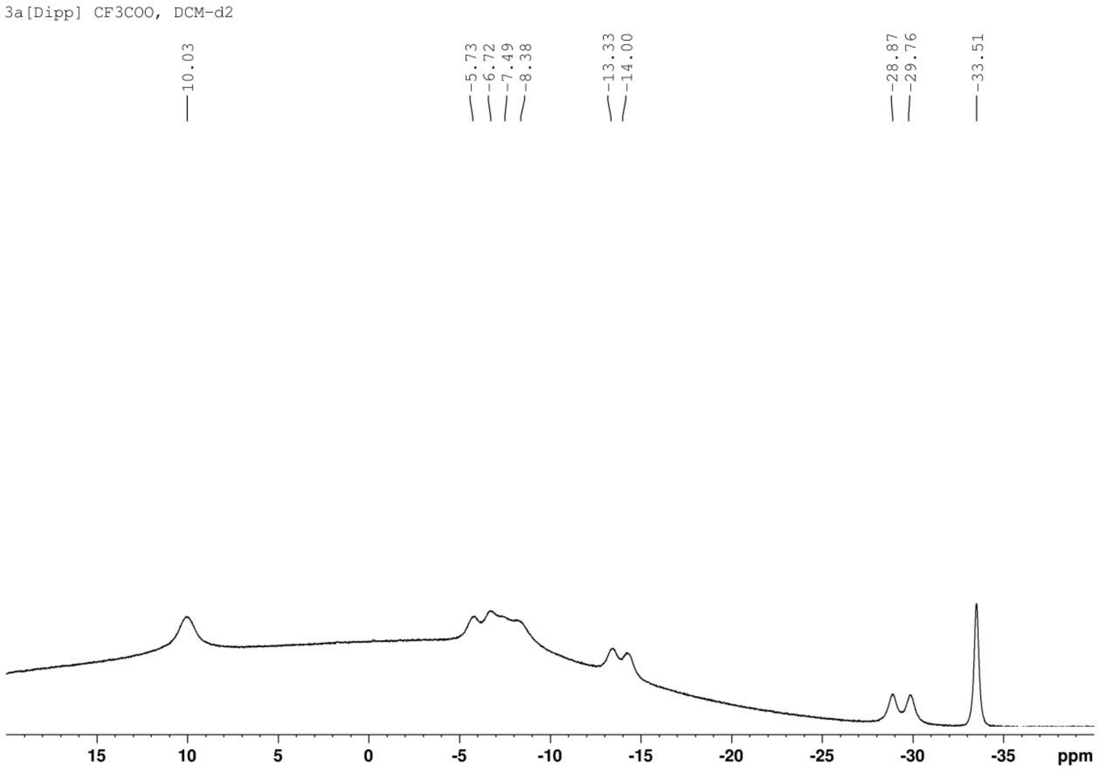

Figure S16. The ^{11}B NMR spectrum of **[3a^{Dipp}][H(TFA)$_2$]**.

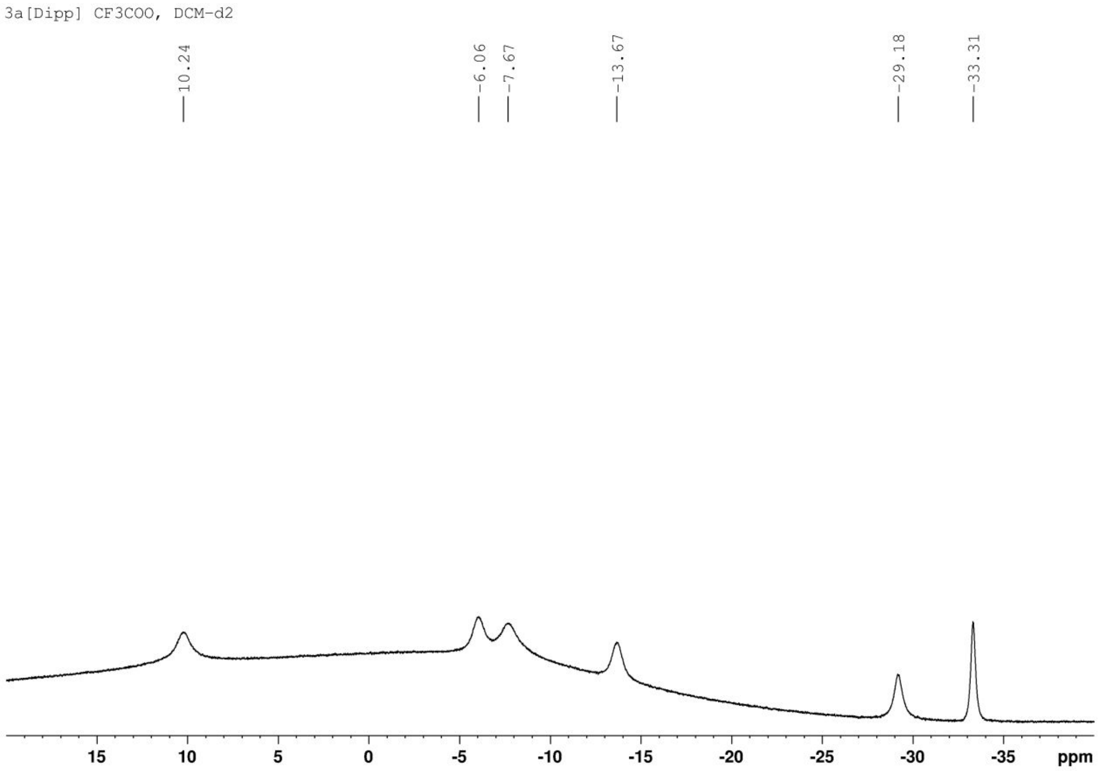

Figure S17. The $^{11}B\{^1H\}$ NMR spectrum of **[3a^{Dipp}][H(TFA)$_2$]**.

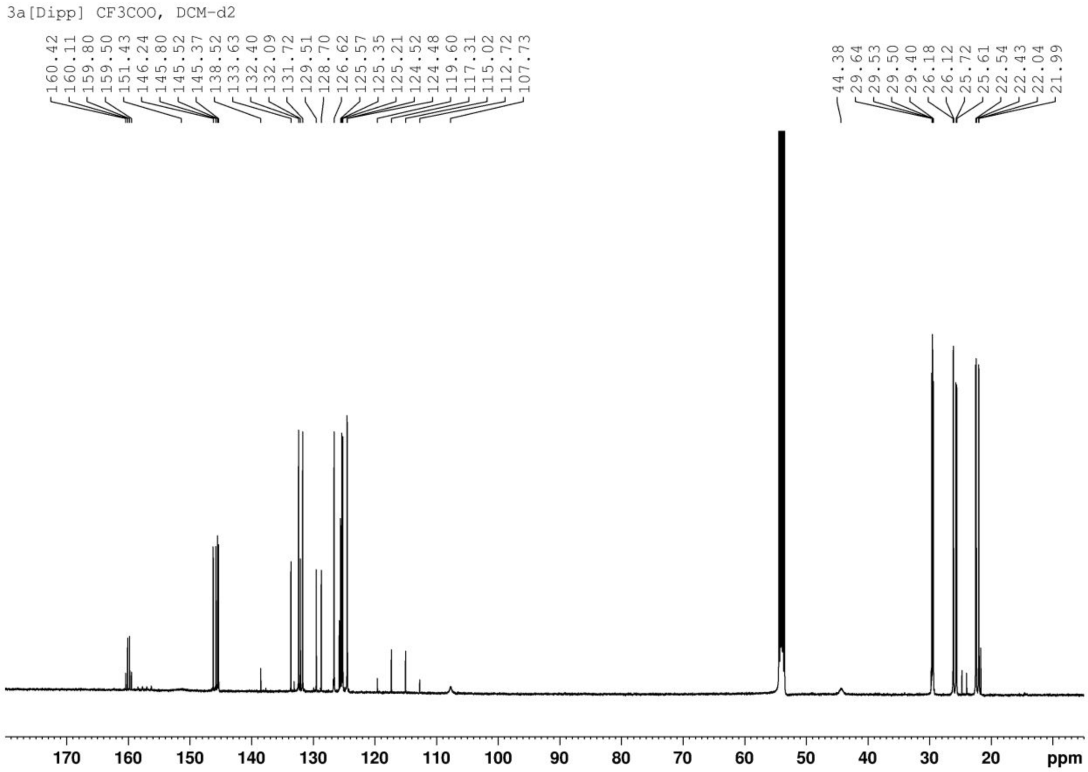

Figure S18. The $^{13}C\{^1H\}$ NMR spectrum of **[3a^{Dipp}][H(TFA)$_2$]**.

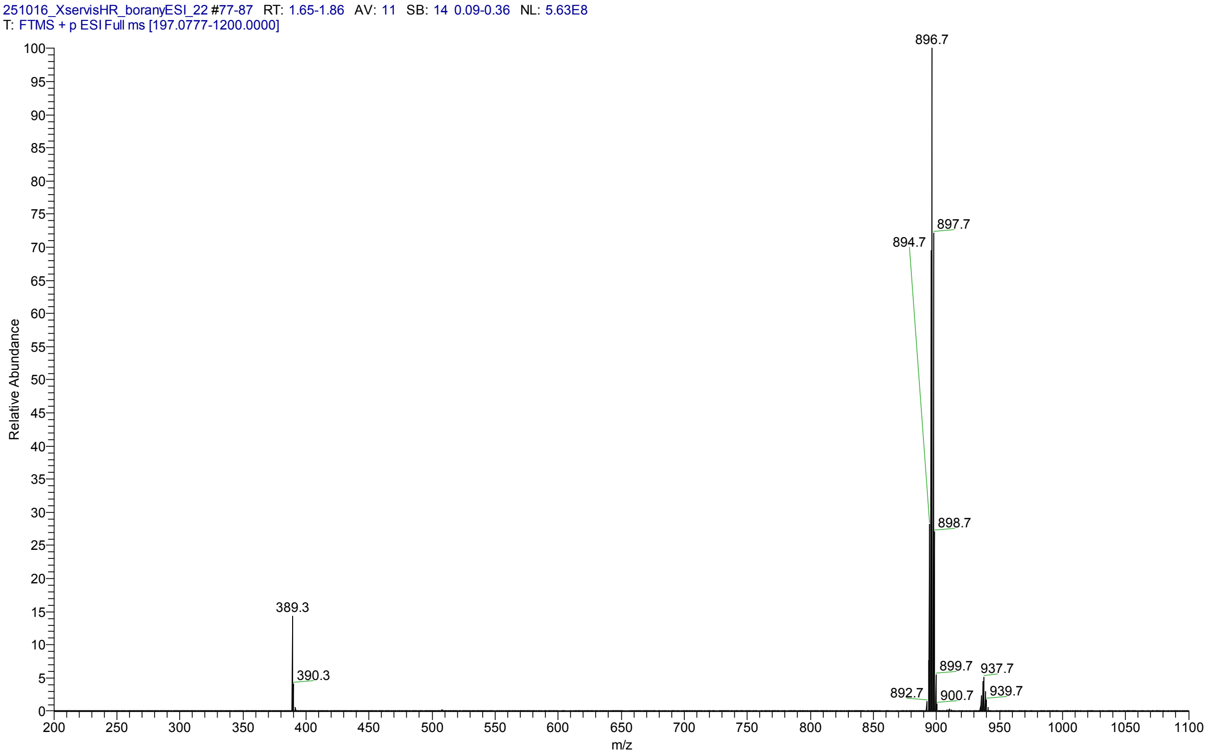

Figure S19. Mass spectrum of positively charged ions (ESI+, Orbitrap) for **[3a^{Dipp}][H(TFA)$_2$]**.

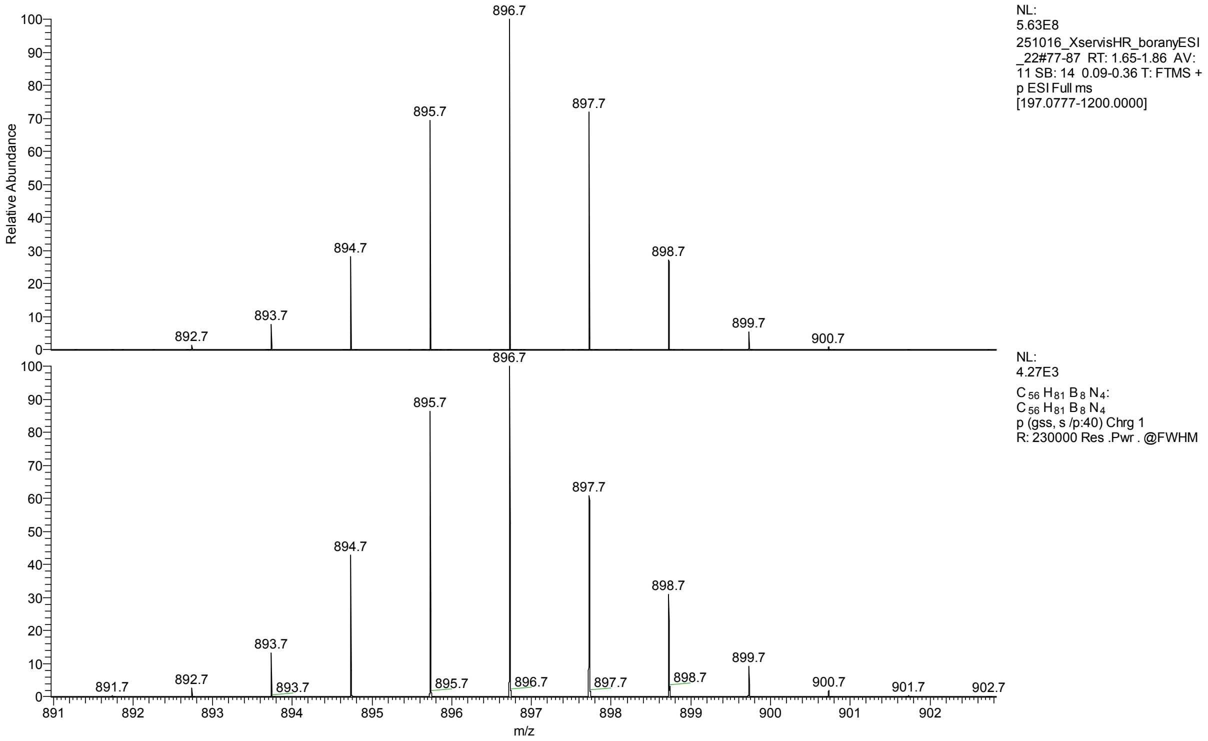

Figure S20. Spectrum of positively charged ions (ESI+, Orbitrap @ R=500,000) for **[3a^{Dipp}][H(TFA)$_2$]** enlarged in the protonated molecule region (top) and simulated spectrum (bottom).

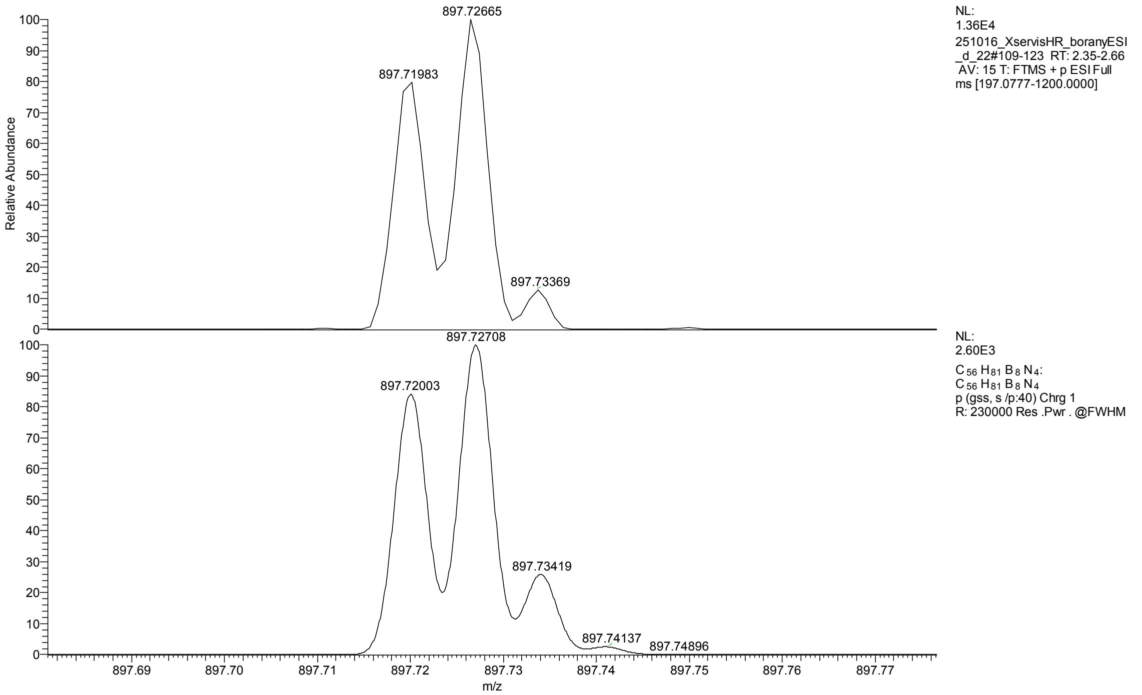

Figure S21. Spectrum of positively charged ions (ESI+, Orbitrap @ R=500,000) for **[3a^{Dipp}][H(TFA)$_2$]** enlarged in the monoisotopic peak region (top) and simulated spectrum (bottom). Theoretical mass for $C_{56}H_{81}N_4B_8^+$: *m/z* 897.72002; experimental mass: *m/z* 897.71983; mass error -0.21 ppm.

Spectroscopic characterization of 1^{MIC}.

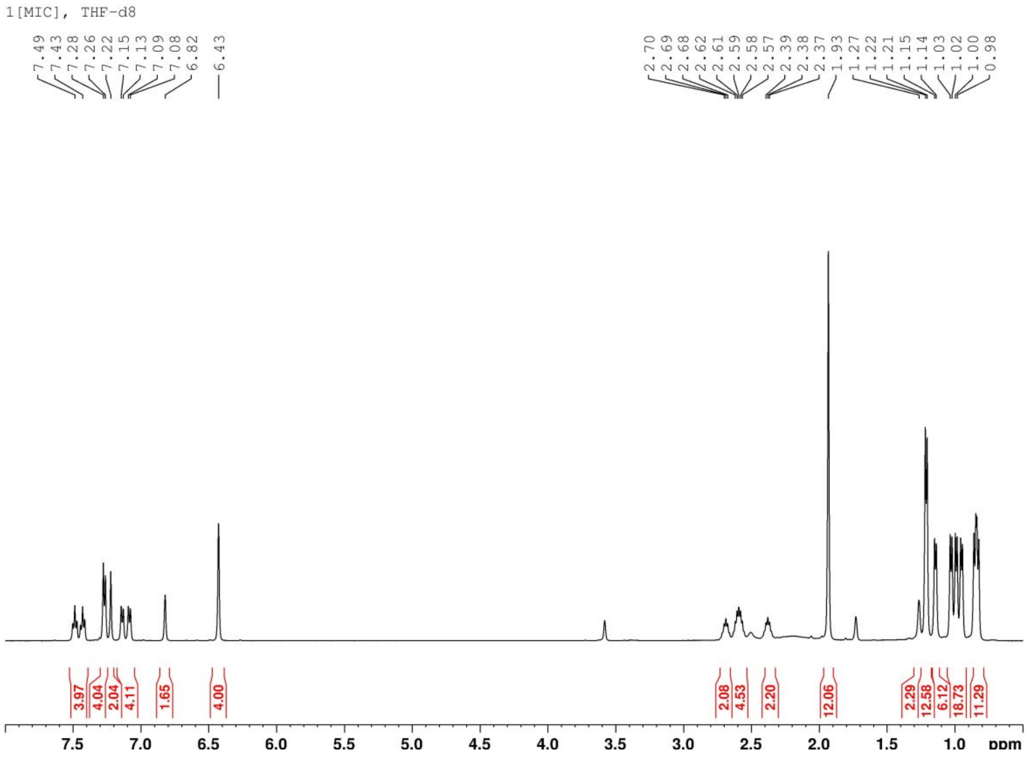

Figure S22. The ^{1}H NMR spectrum of **1^{MIC}**.

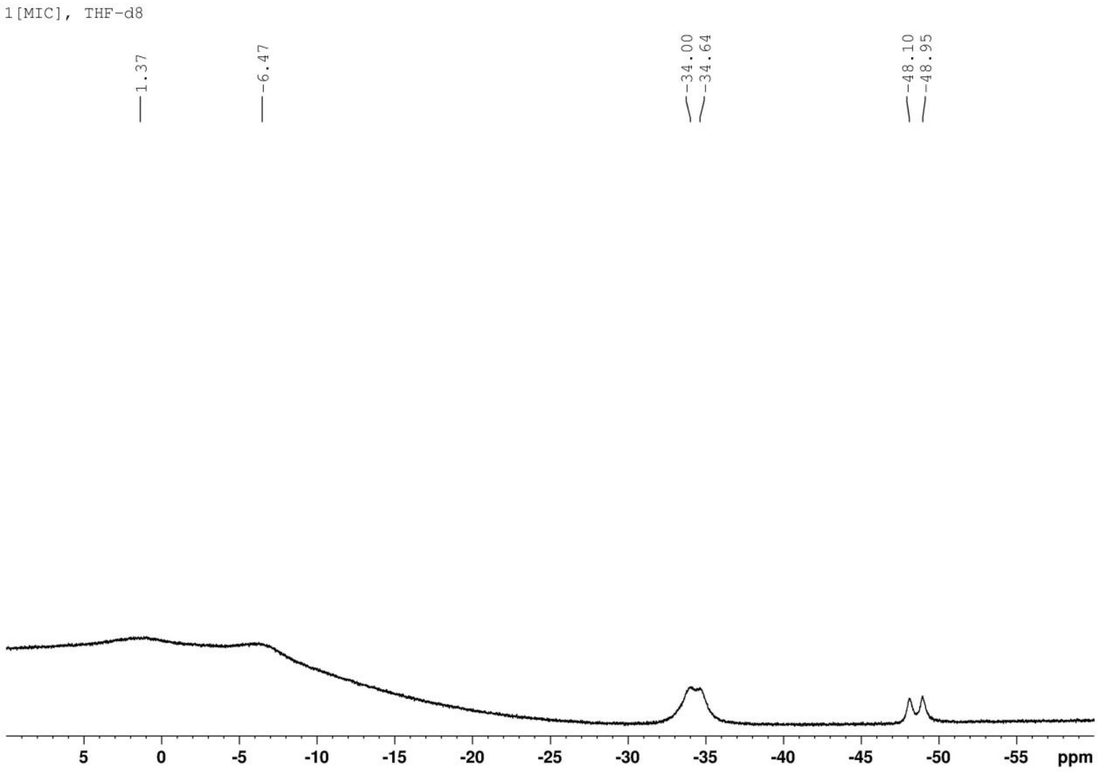

Figure S23. The ^{11}B NMR spectrum of $\mathbf{1}^{\mathbf{MIC}}$.

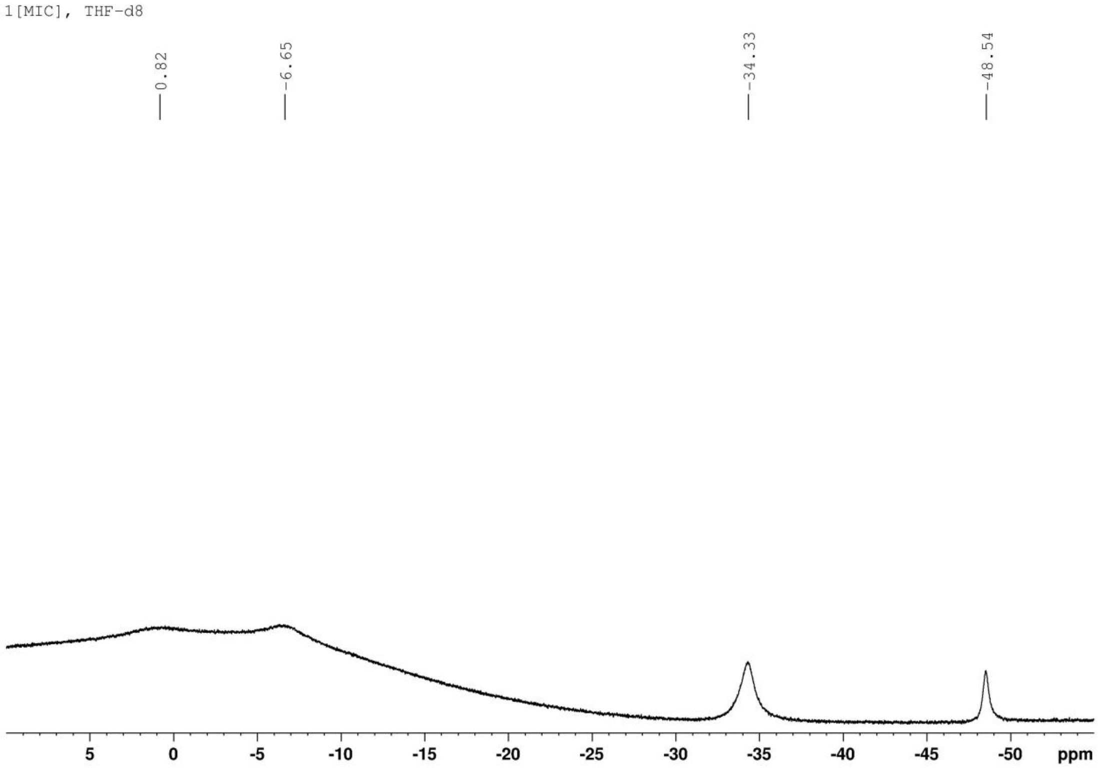

Figure S24. The $^{11}B\{^{1}H\}$ NMR spectrum of $\mathbf{1}^{\mathbf{MIC}}$.

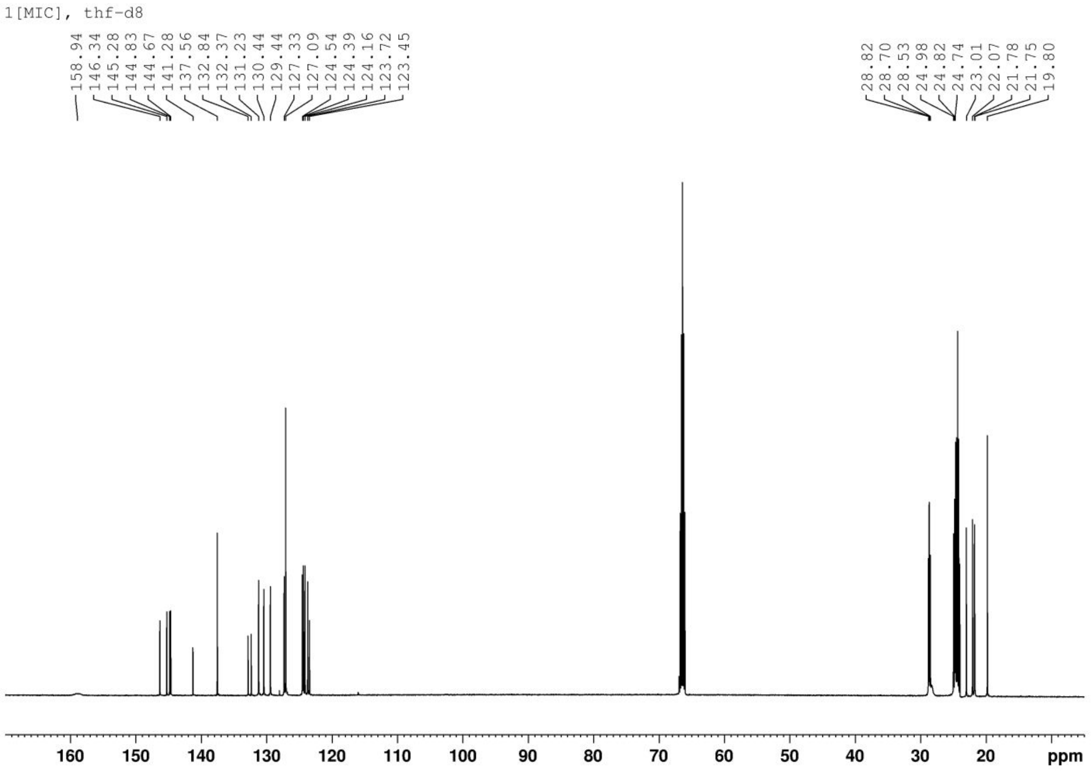

Figure S25. The $^{13}C\{^{1}H\}$ NMR spectrum of $\mathbf{1^{MIC}}$.

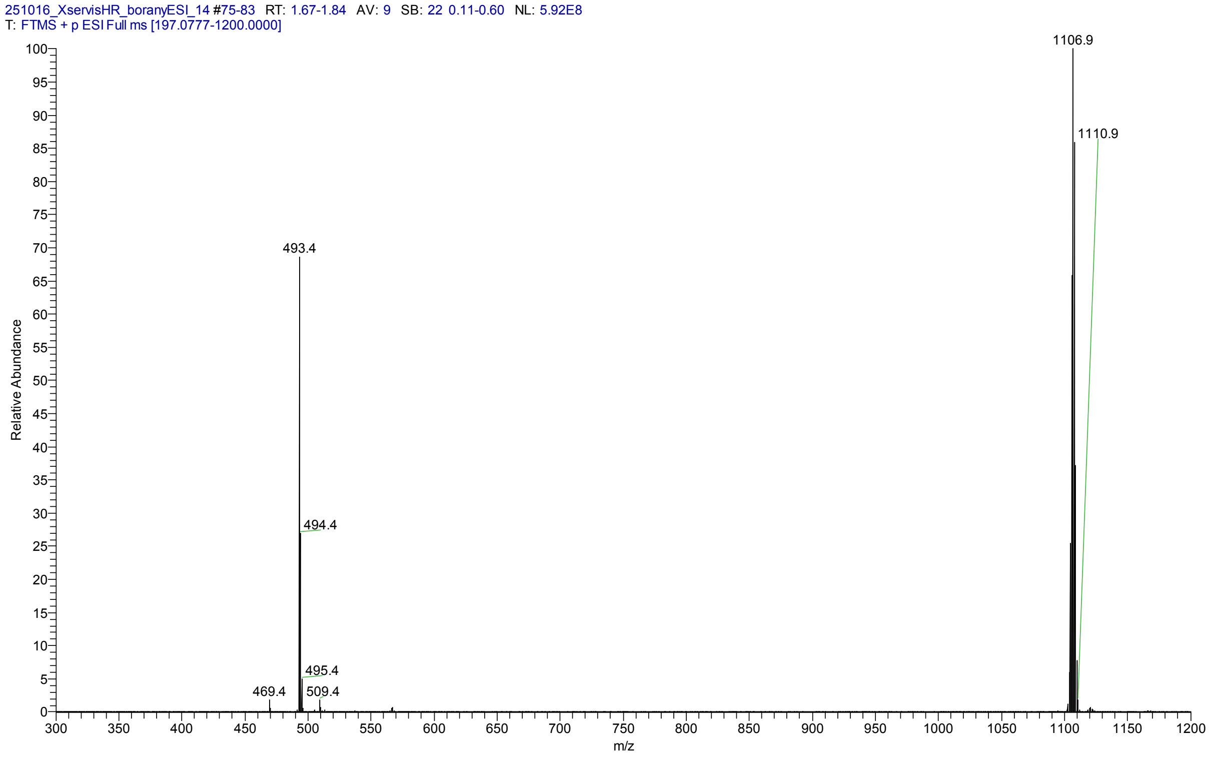

Figure S26. Mass spectrum of positively charged ions (ESI+, Orbitrap) for $\mathbf{1^{MIC}}$.

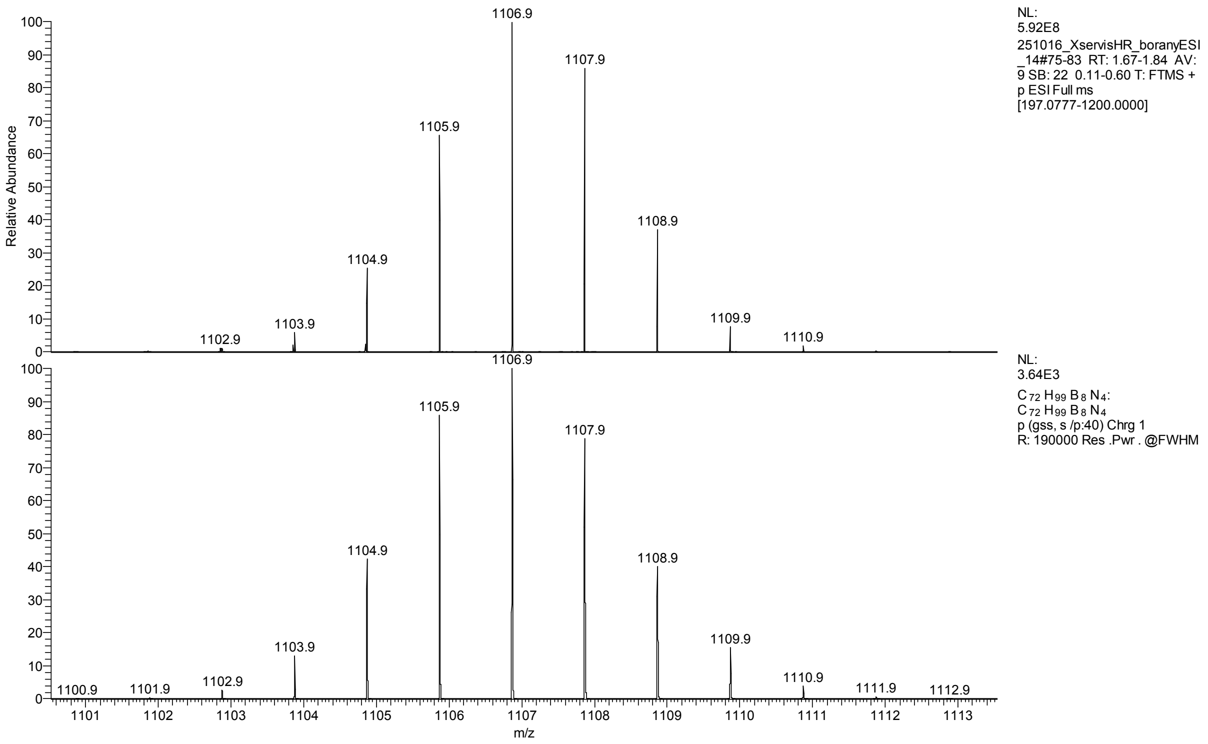

Figure S27. Spectrum of positively charged ions (ESI+, Orbitrap @ R=500,000) for **1^MIC^** enlarged in the protonated molecule region (top) and simulated spectrum (bottom).

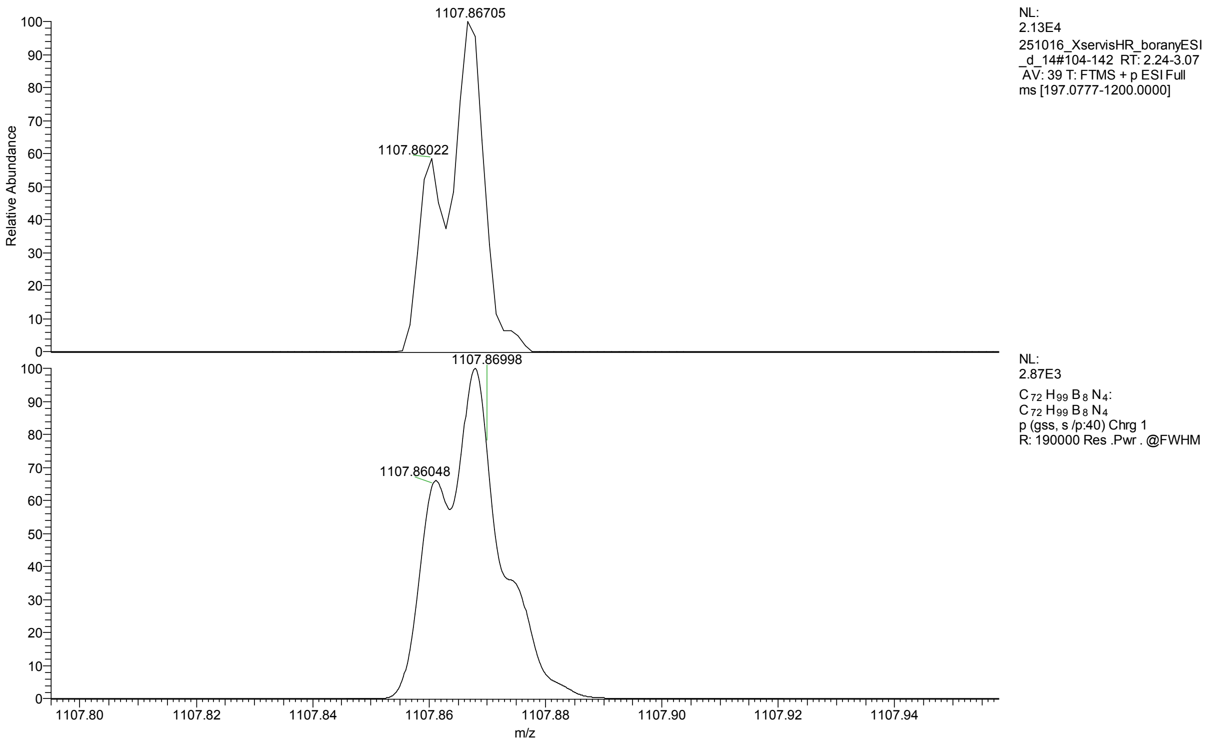

Figure S28. Spectrum of positively charged ions (ESI+, Orbitrap @ R=500,000) for **1^MIC^** enlarged in the monoisotopic peak region (top) and simulated spectrum (bottom). Theoretical mass for $C_{72}H_{99}N_4B_8^+$: *m/z* 1107.86087; experimental mass: *m/z* 1107.86022; mass error -0.59 ppm.

Spectroscopic characterization of 2^{MIC}.

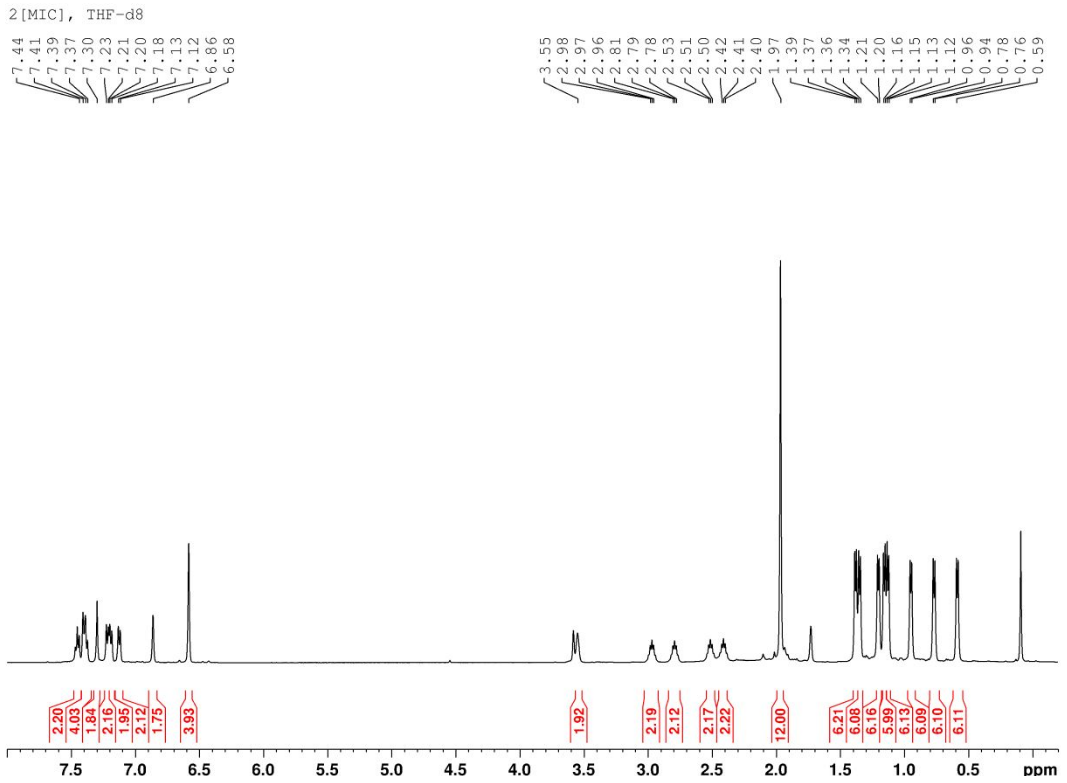

Figure S29. The ^{1}H NMR spectrum of $\mathbf{2^{MIC}}$.

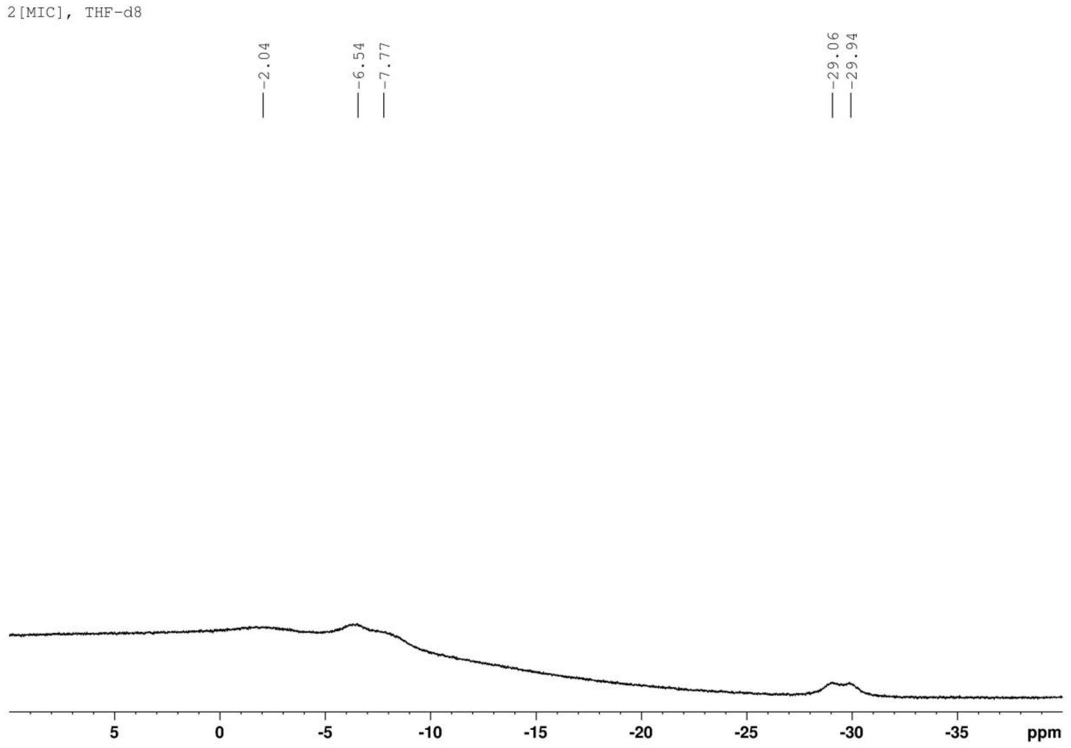

Figure S30. The ^{11}B NMR spectrum of $\mathbf{2^{MIC}}$.

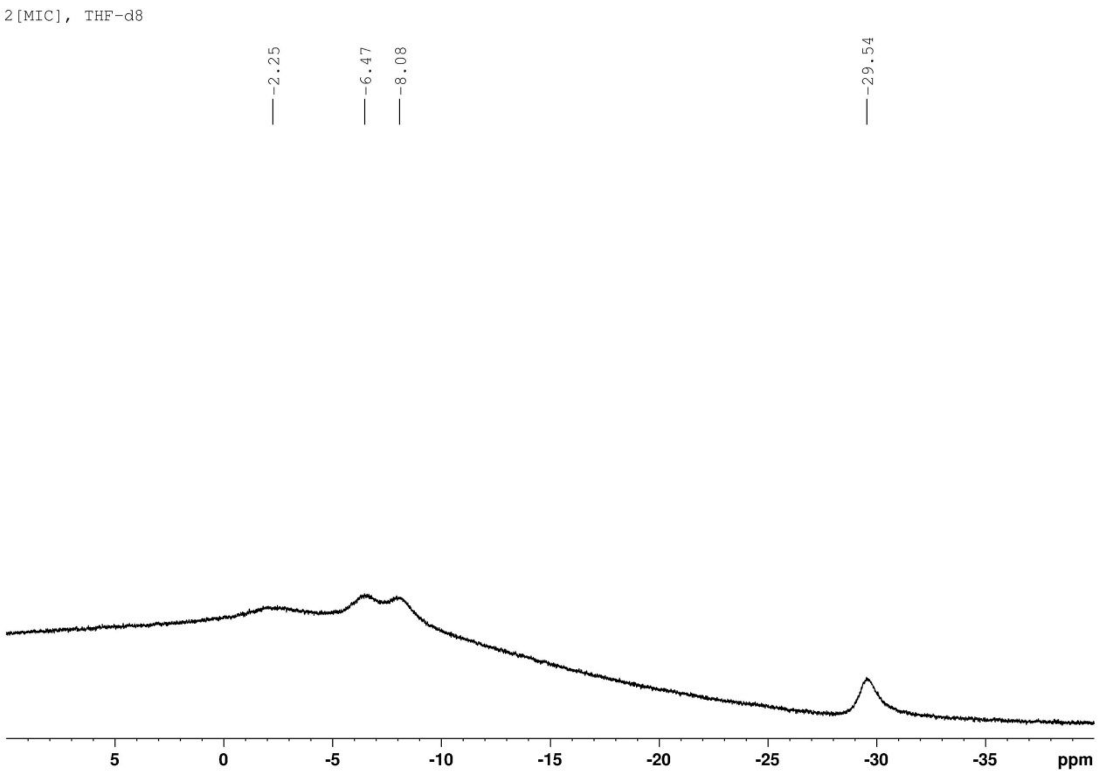

Figure S31. The $^{11}B\{^1H\}$ NMR spectrum of $\mathbf{2}^{\mathbf{MIC}}$.

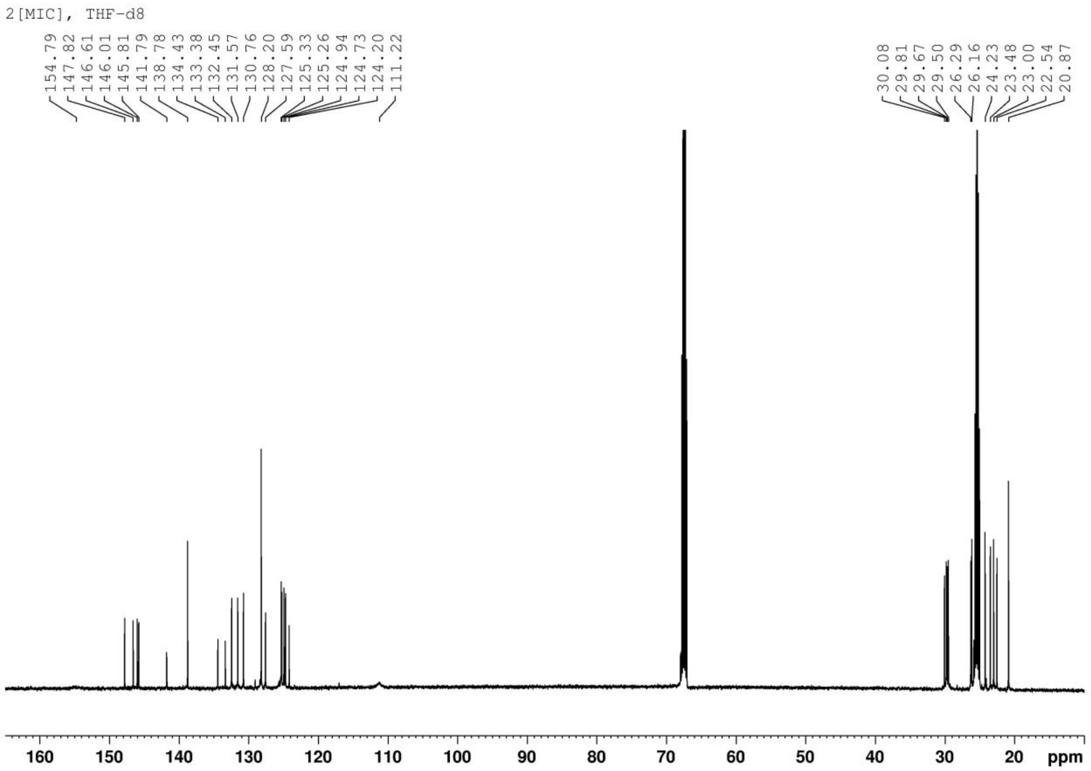

Figure S32. The $^{13}C\{^1H\}$ NMR spectrum of $\mathbf{2}^{\mathbf{MIC}}$.

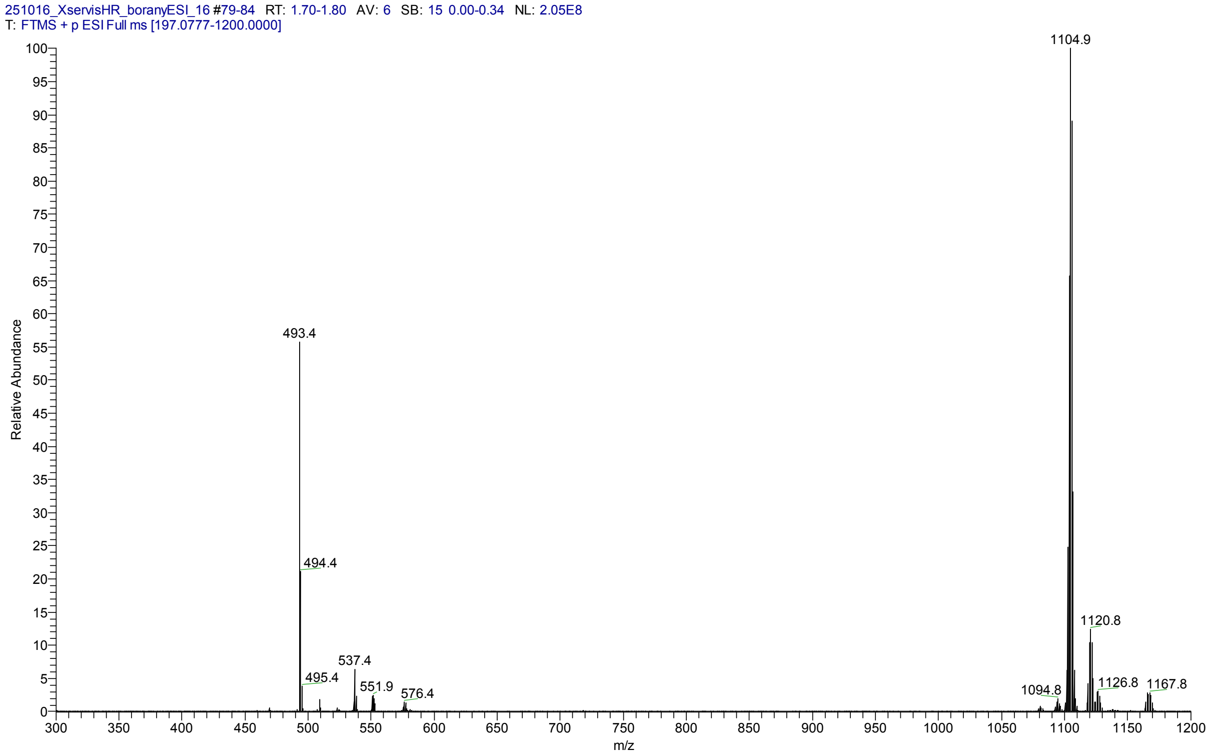

Figure S33. Mass spectrum of positively charged ions (ESI+, Orbitrap) for **2**$^{\mathbf{MIC}}$.

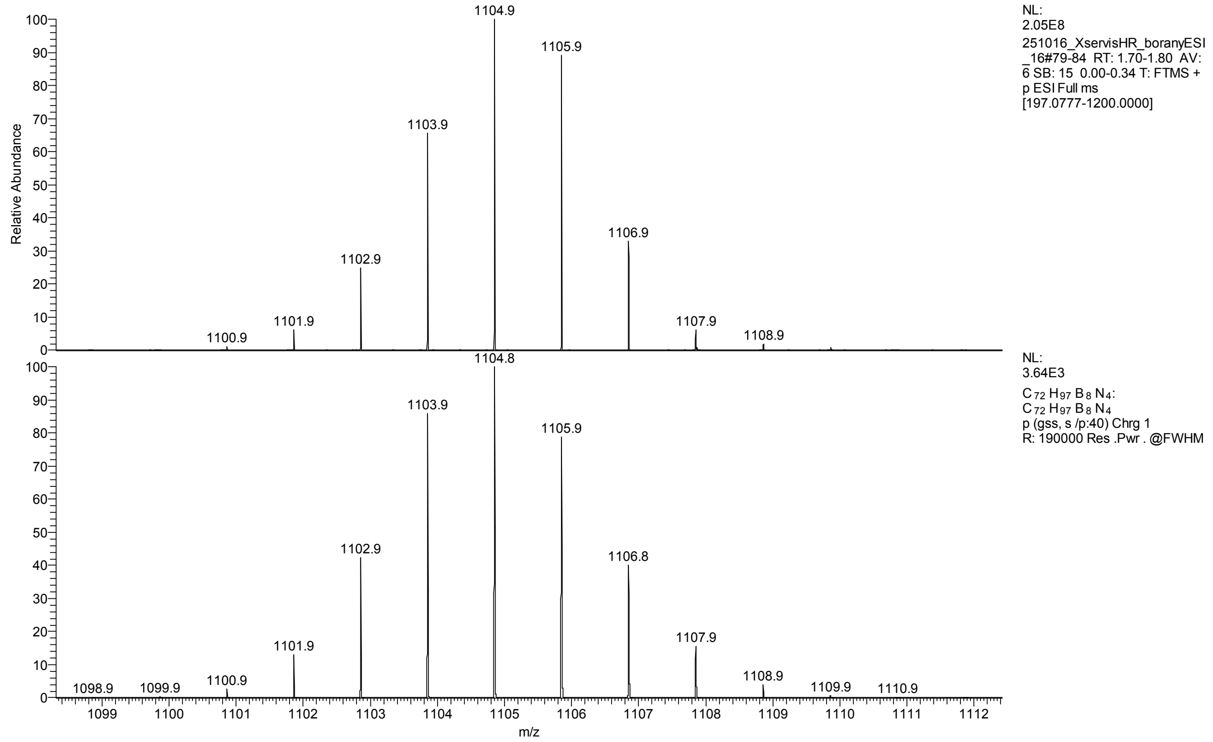

Figure S34. Spectrum of positively charged ions (ESI+, Orbitrap @ R=500,000) for **2**$^{\mathbf{MIC}}$ enlarged in the protonated molecule region (top) and simulated spectrum (bottom).

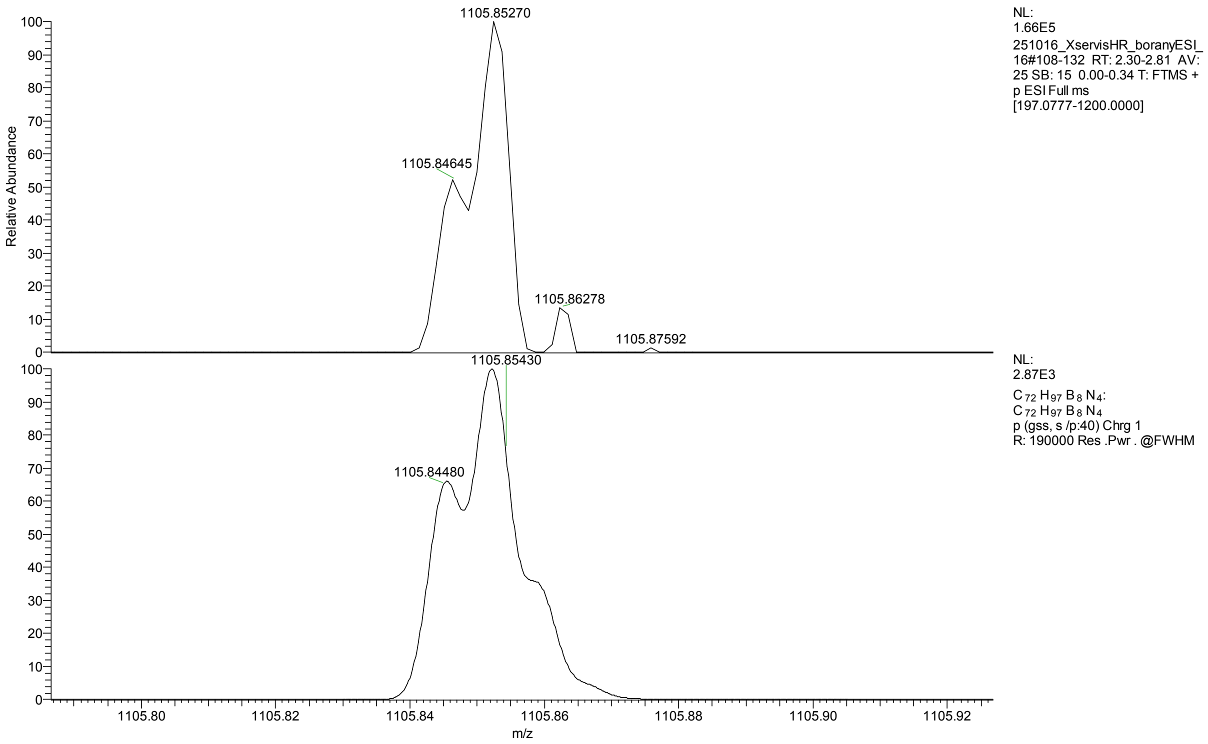

Figure S35. Spectrum of positively charged ions (ESI+, Orbitrap @ R=500,000) for **2^MIC^** enlarged in the monoisotopic peak region (top) and simulated spectrum (bottom). Theoretical mass for $C_{72}H_{97}N_4B_8^+$: *m/z* 1105.84522; experimental mass: *m/z* 1105.84645; mass error 1.12 ppm.

Spectroscopic characterization of 1^*i*Pr^.

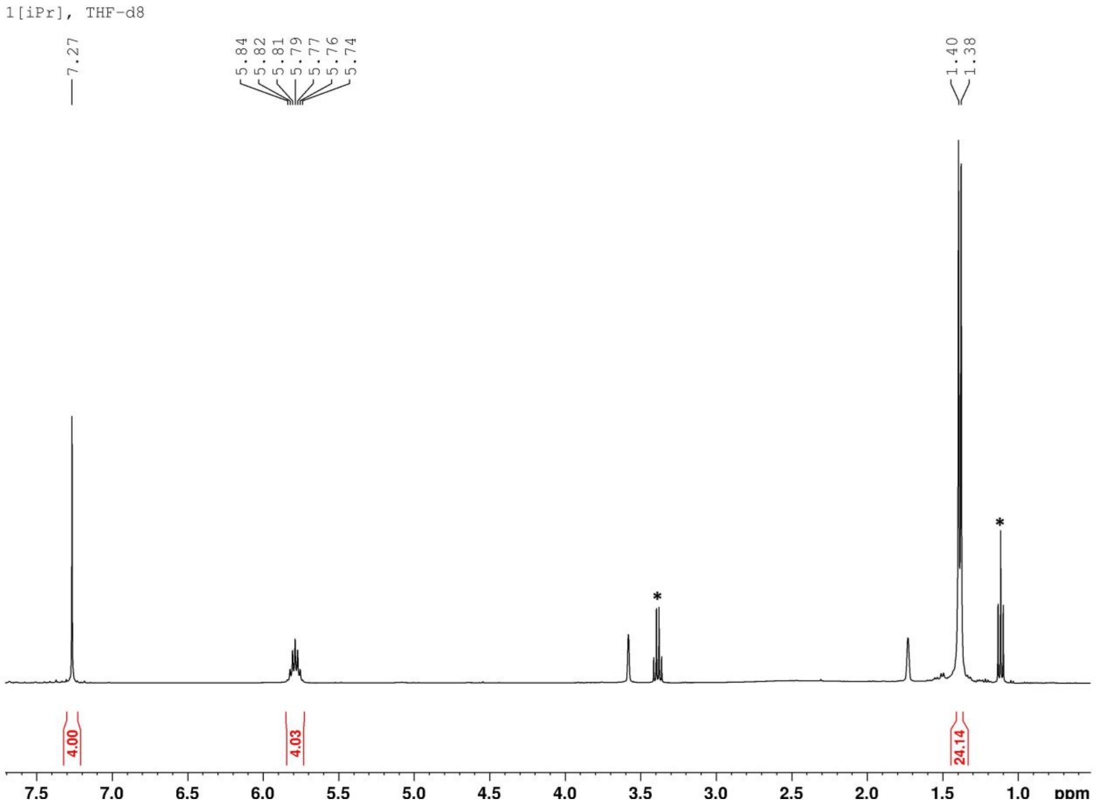

Figure S36. The ^{1}H NMR spectrum of **1^*i*Pr^**. Residual diethyl ether is marked by *.

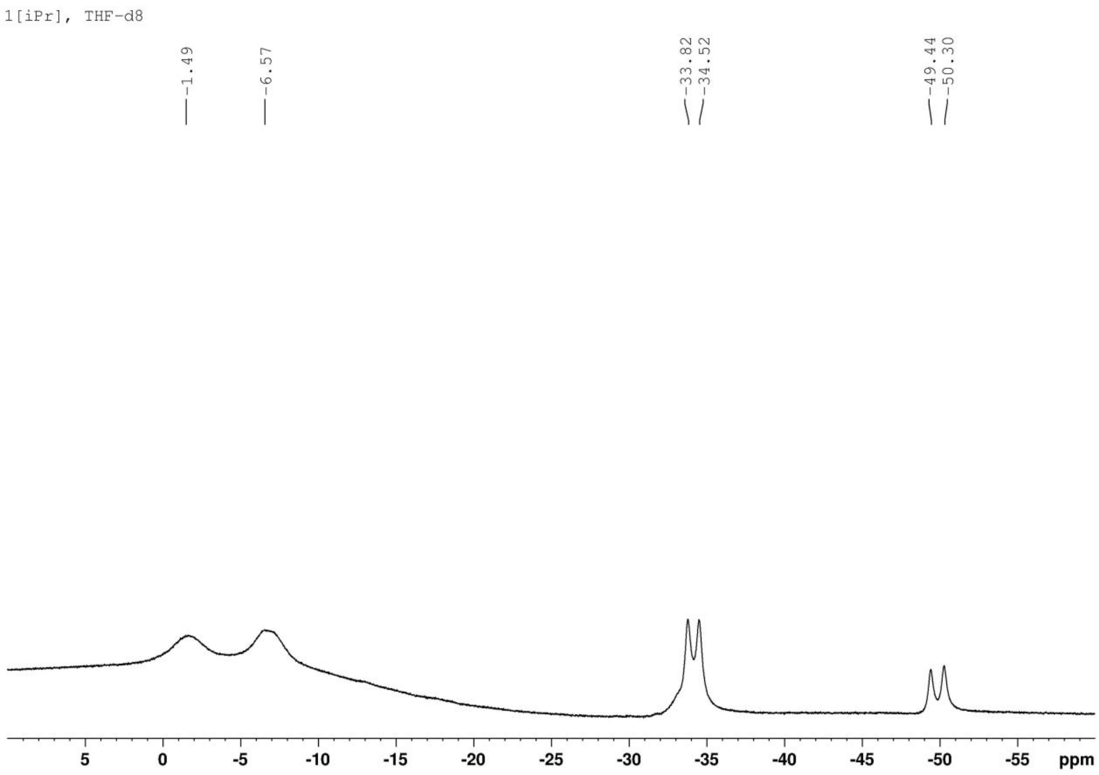

Figure S37. The ^{11}B NMR spectrum of $\mathbf{1}^{iPr}$.

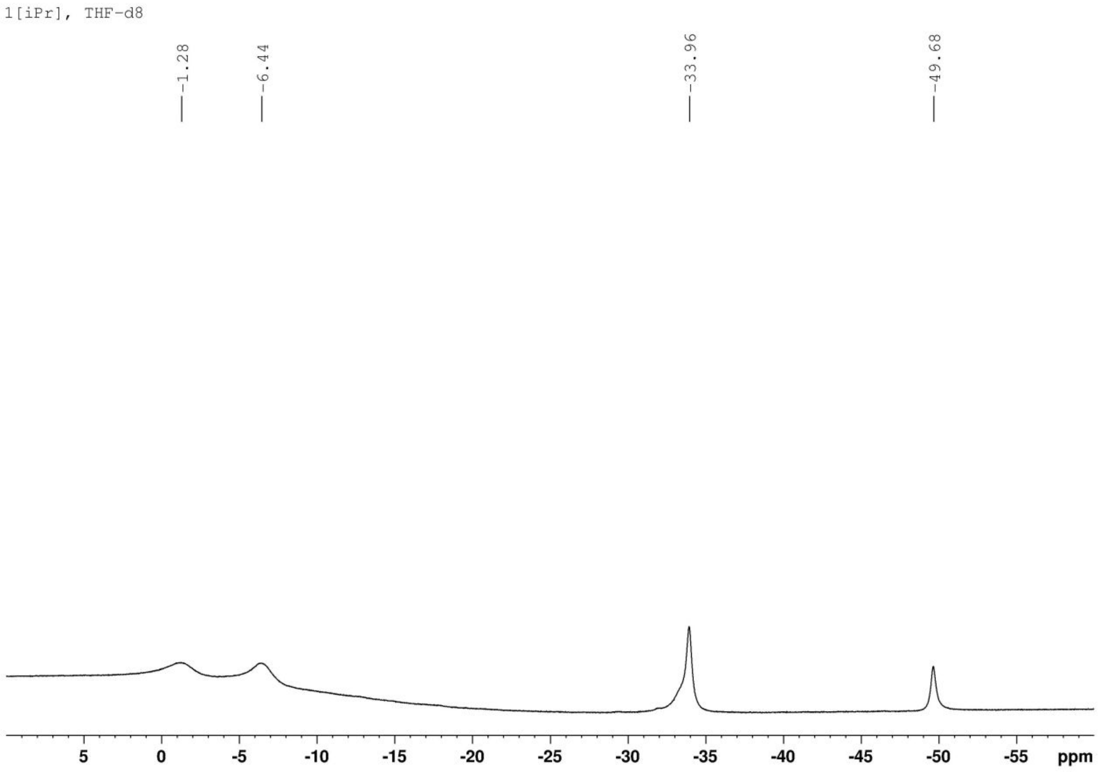

Figure S38. The $^{11}B\{^1H\}$ NMR spectrum of $\mathbf{1}^{iPr}$.

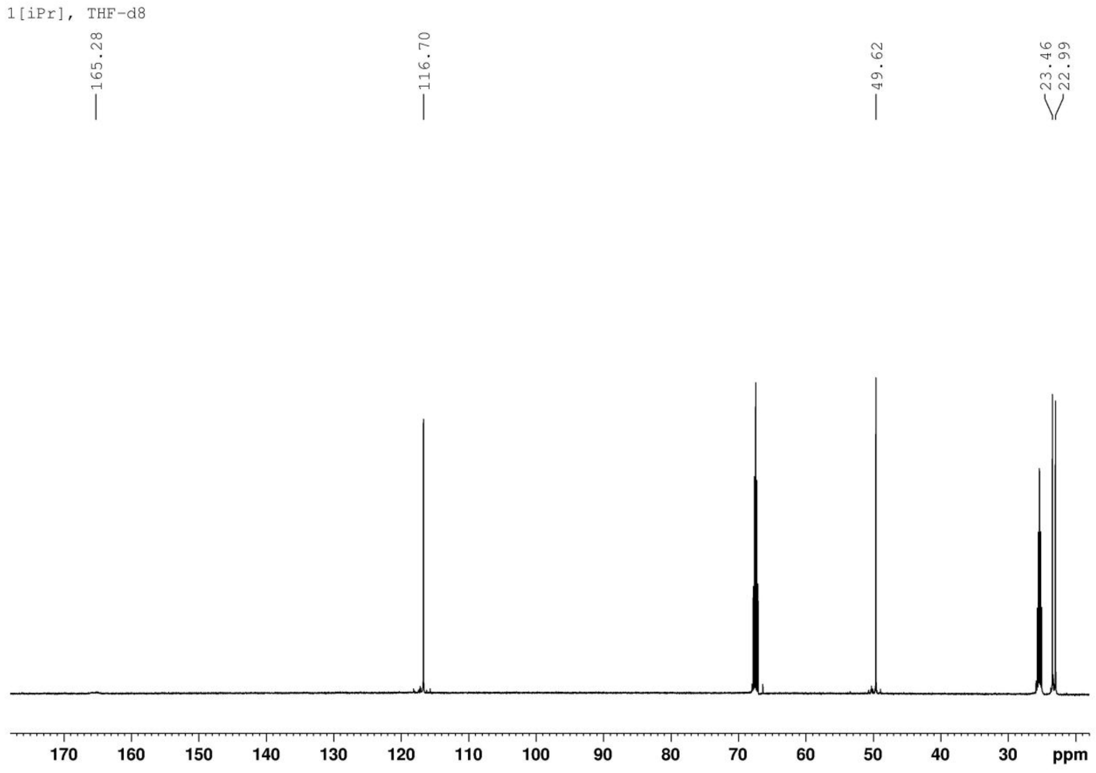

Figure S39. The $^{13}C\{^{1}H\}$ NMR spectrum of **1^{iPr}**.

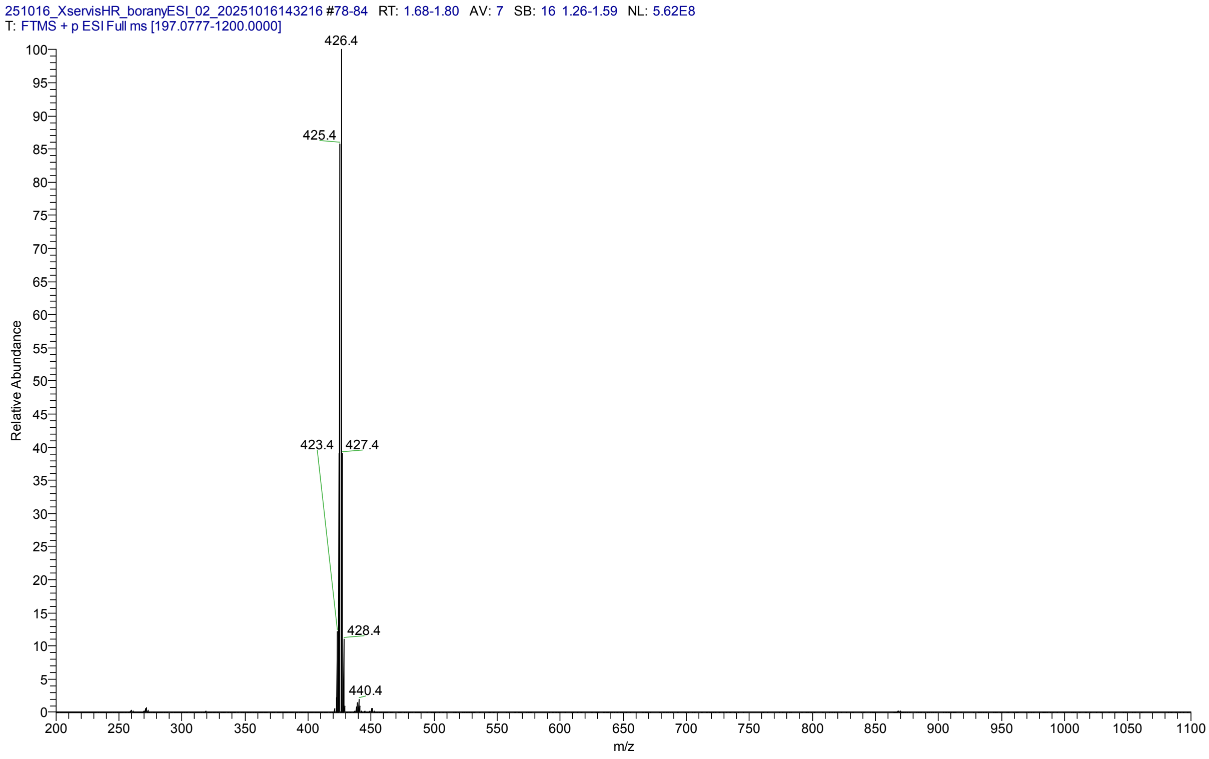

Figure S40. Mass spectrum of positively charged ions (ESI+, Orbitrap) for **1^{iPr}**.

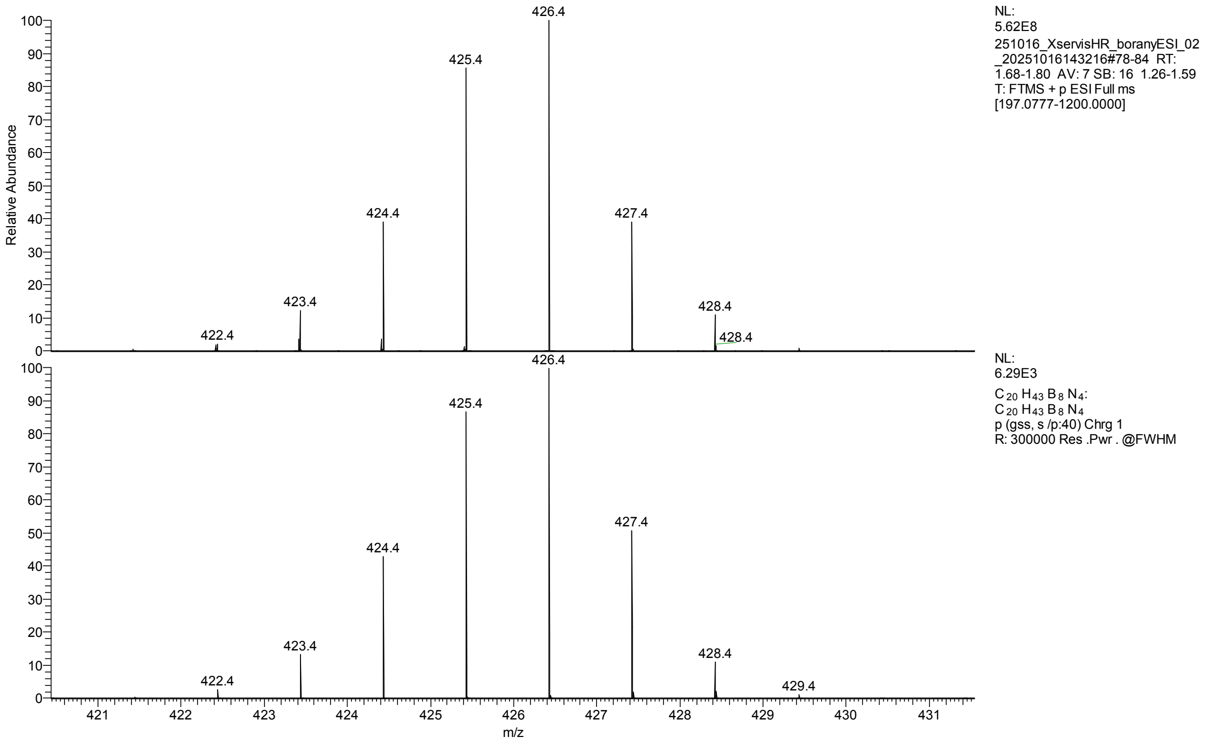

Figure S41. Spectrum of positively charged ions (ESI+, Orbitrap @ R=500,000) for **1**^{iPr} enlarged in the protonated molecule region (top) and simulated spectrum (bottom).

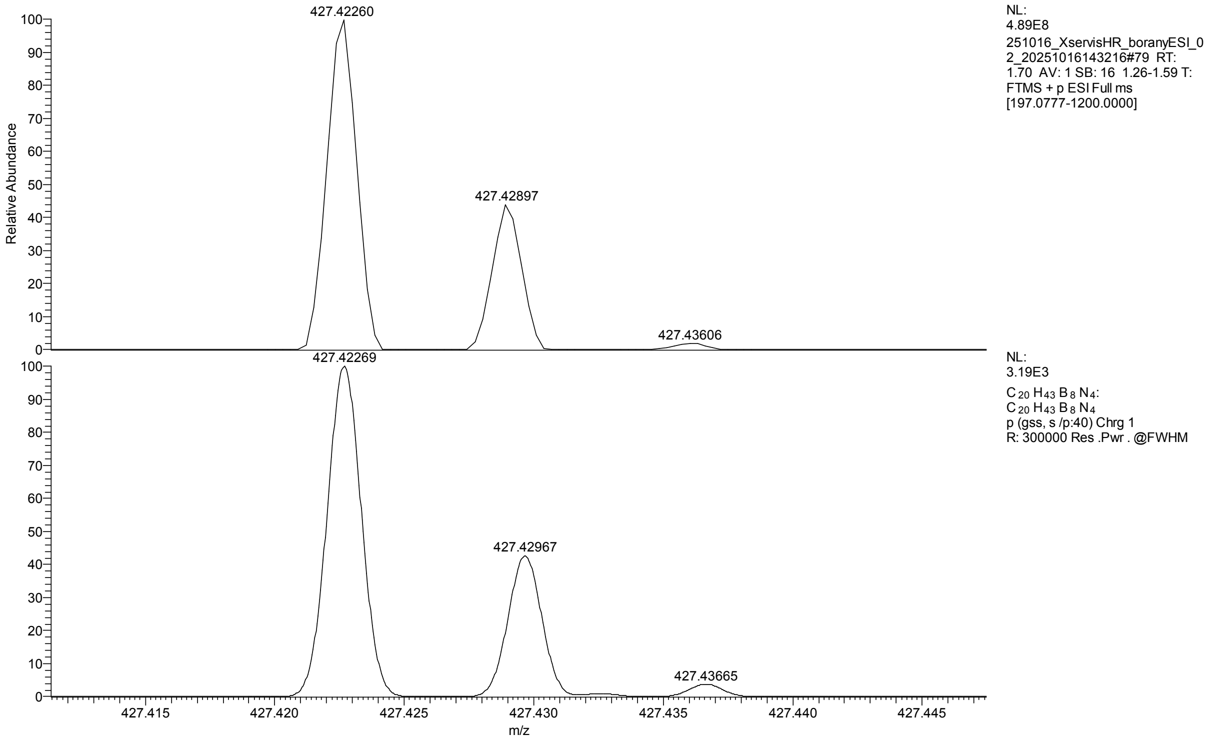

Figure S42. Spectrum of positively charged ions (ESI+, Orbitrap @ R=500,000) for **1**^{iPr} enlarged in the monoisotopic peak region (top) and simulated spectrum (bottom). Theoretical

mass for $C_{20}H_{43}N_4B_8^+$: *m/z* 427.42267; experimental mass: *m/z* 427.42260; mass error -0.16 ppm.

Spectroscopic characterization of 2a^{iPr}.

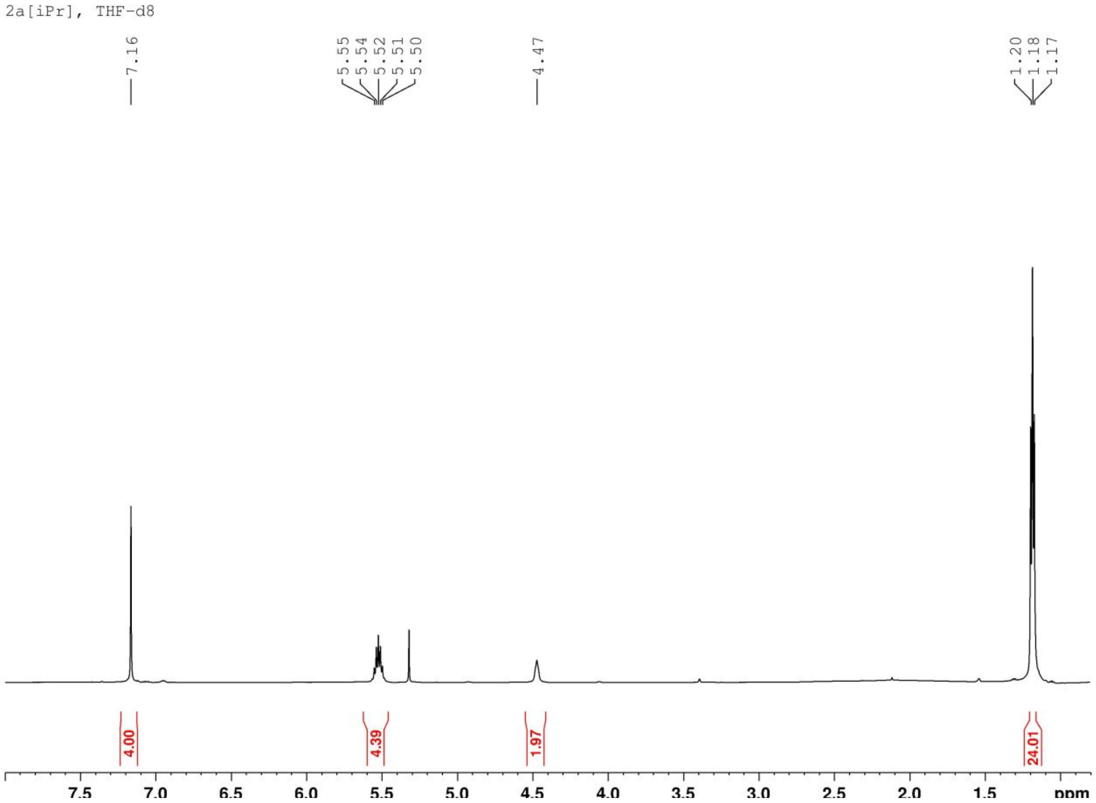

Figure S43. The ^{1}H NMR spectrum of **2a^{iPr}**.

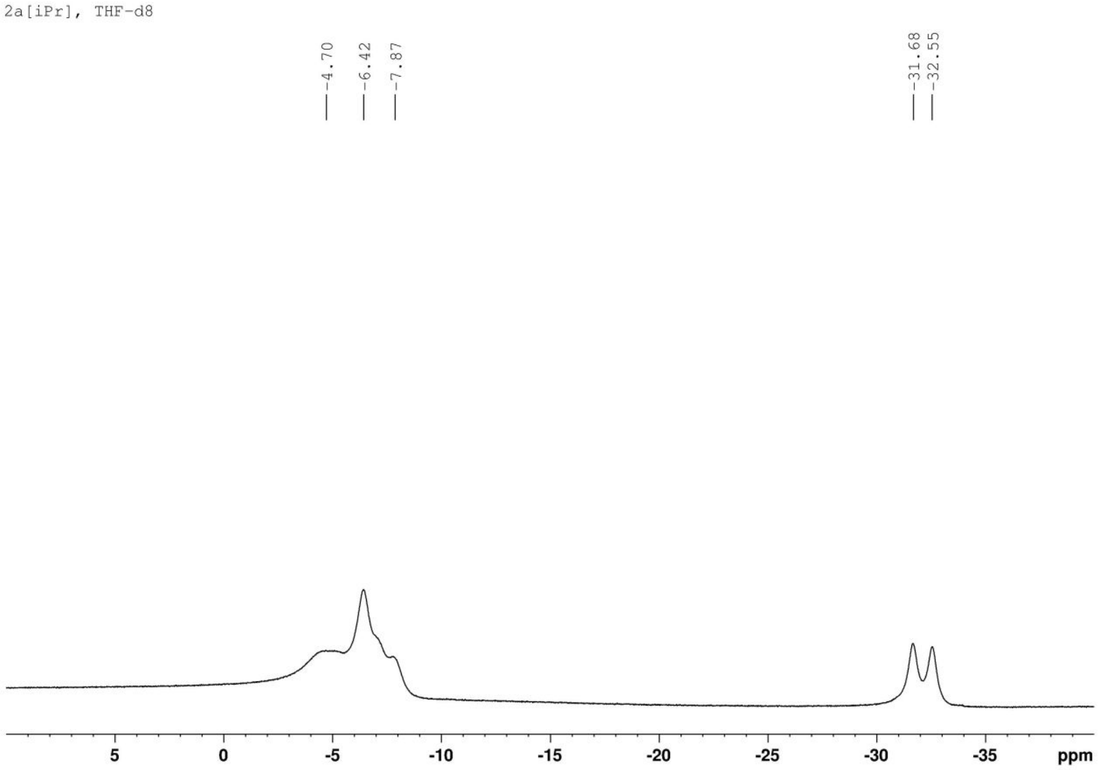

Figure S44. The ${}^{11}B$ NMR spectrum of **2a^{iPr}**.

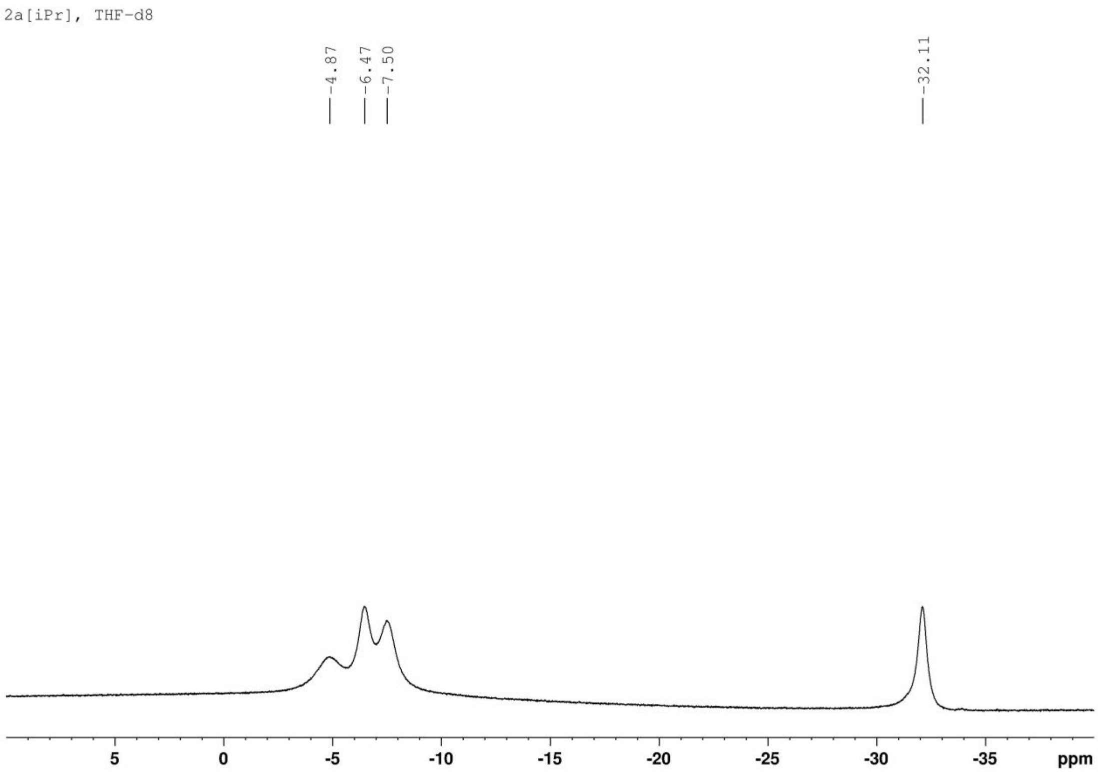

Figure S45. The ${}^{11}B\{{}^{1}H\}$ NMR spectrum of **2a^{iPr}**.

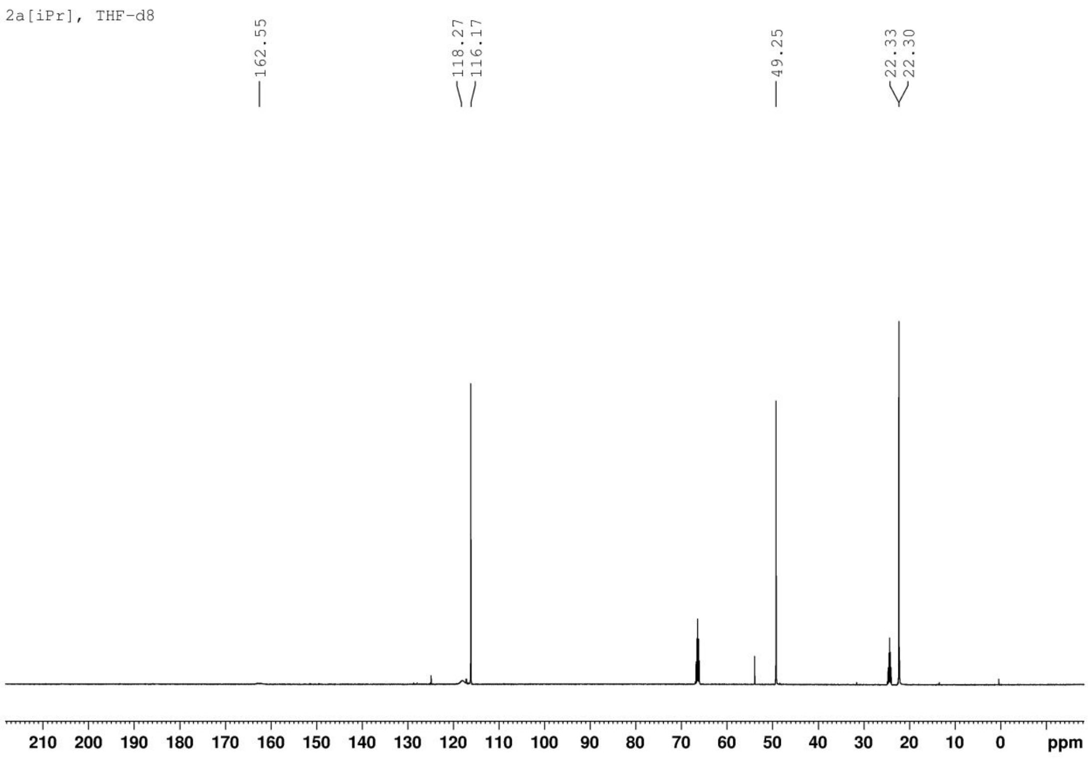

Figure S46. The ${}^{13}C\{{}^{1}H\}$ NMR spectrum of **2a^{iPr}**.

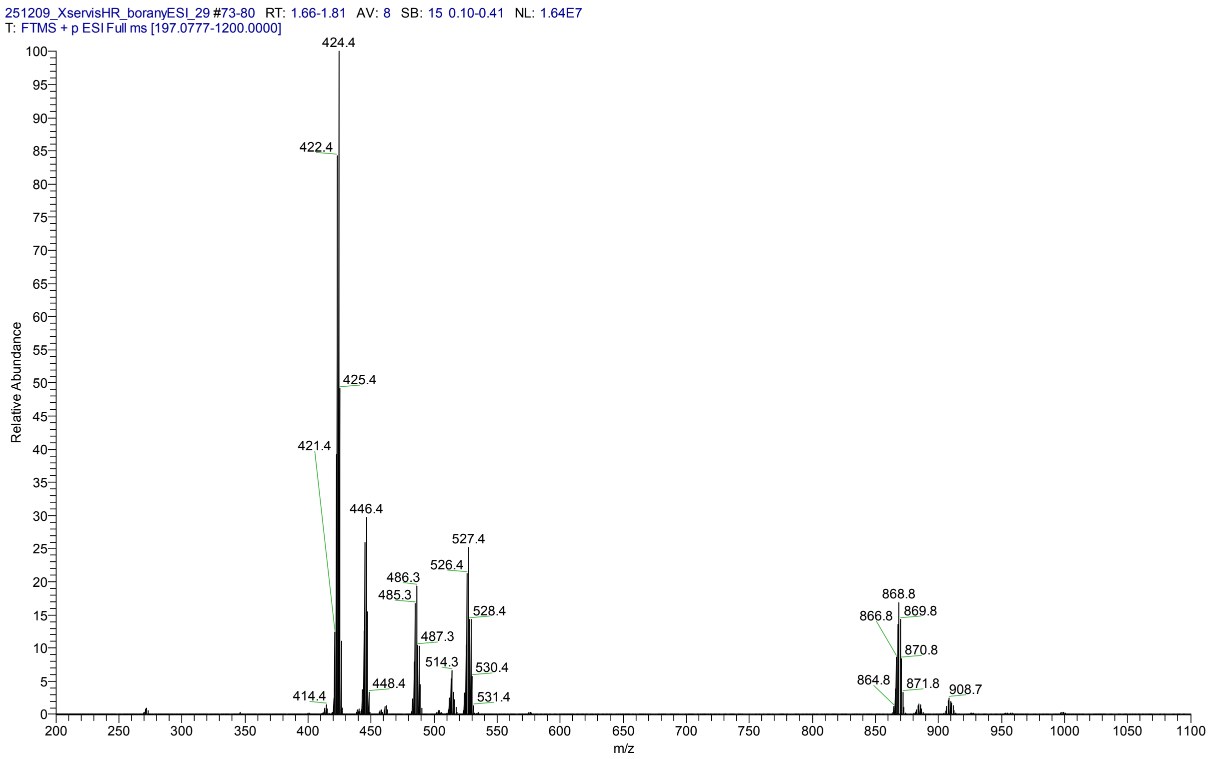

Figure S47. Mass spectrum of positively charged ions (ESI+, Orbitrap) for **2a**^{iPr}.

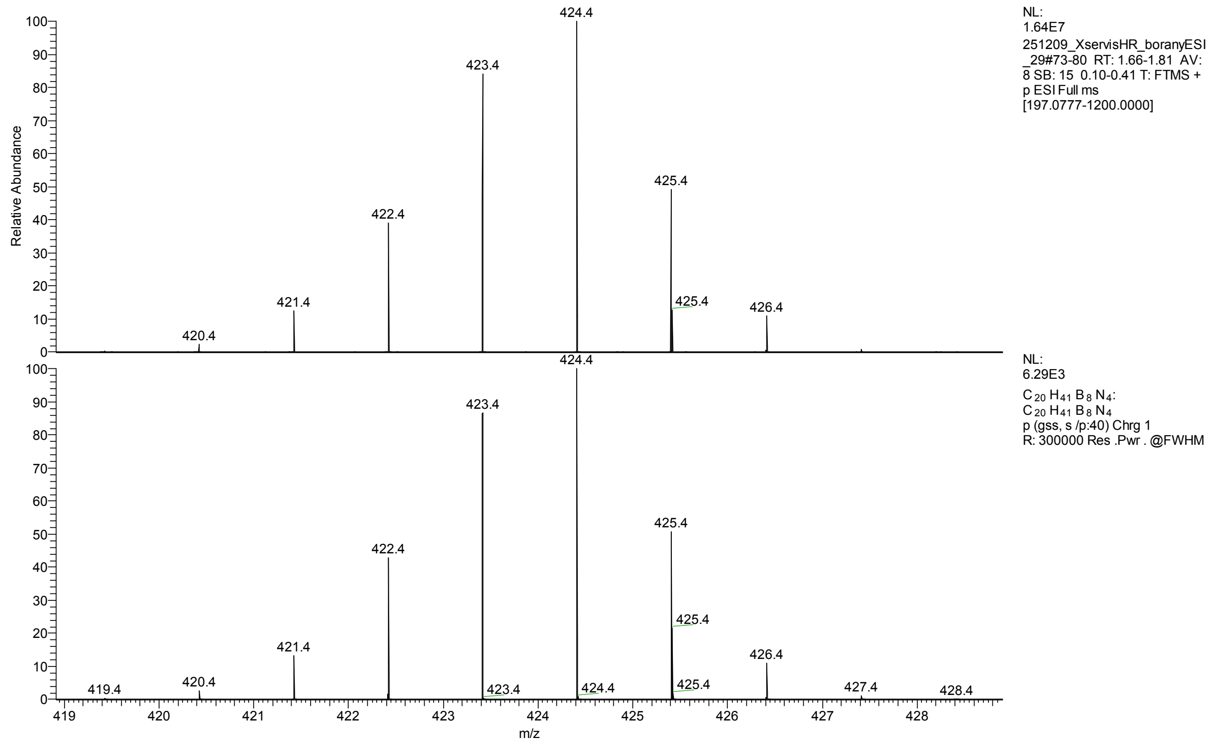

Figure S48. Spectrum of positively charged ions (ESI+, Orbitrap @ R=500,000) for **2a**^{iPr} enlarged in the protonated molecule region (top) and simulated spectrum (bottom).

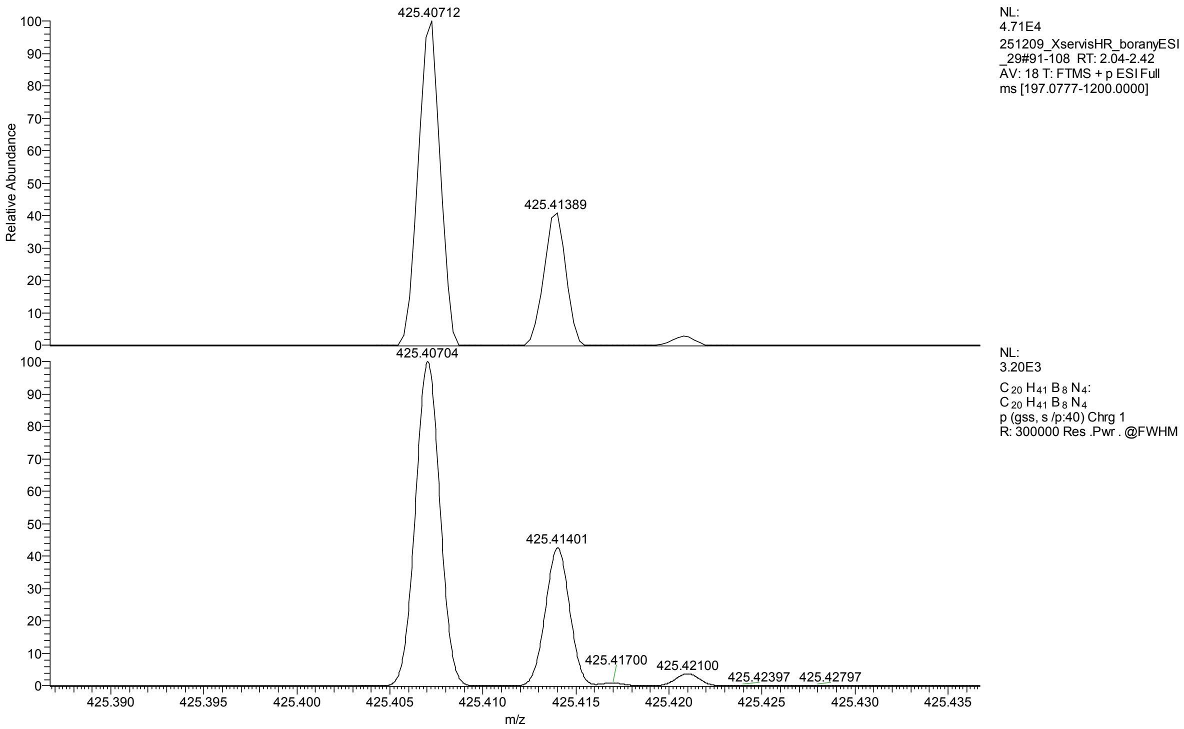

Figure S49. Spectrum of positively charged ions (ESI+, Orbitrap @ R=500,000) for **2a**** enlarged in the monoisotopic peak region (top) and simulated spectrum (bottom). Theoretical mass for $C_{20}H_{41}N_4B_8^+$: *m/z* 425.40702; experimental mass: *m/z* 425.40712; mass error 0.25 ppm.

Spectroscopic characterization of 2b^{iPr}.

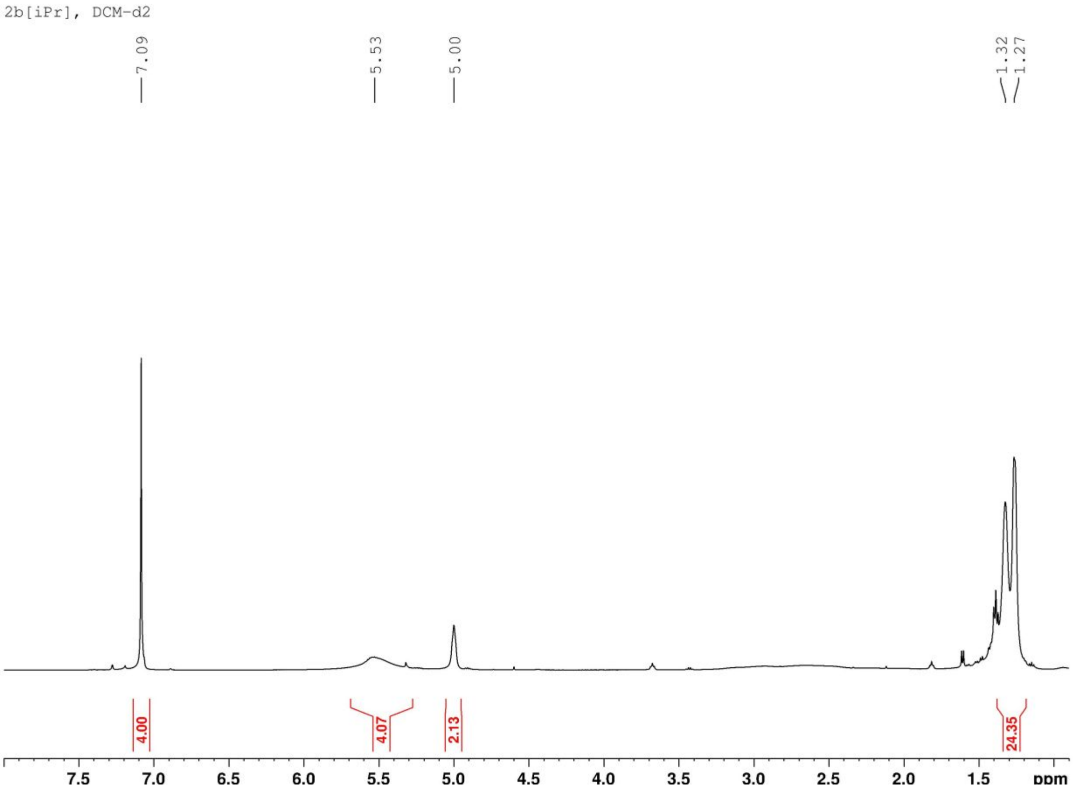

Figure S50. The ^{1}H NMR spectrum of **2b^{iPr}**.

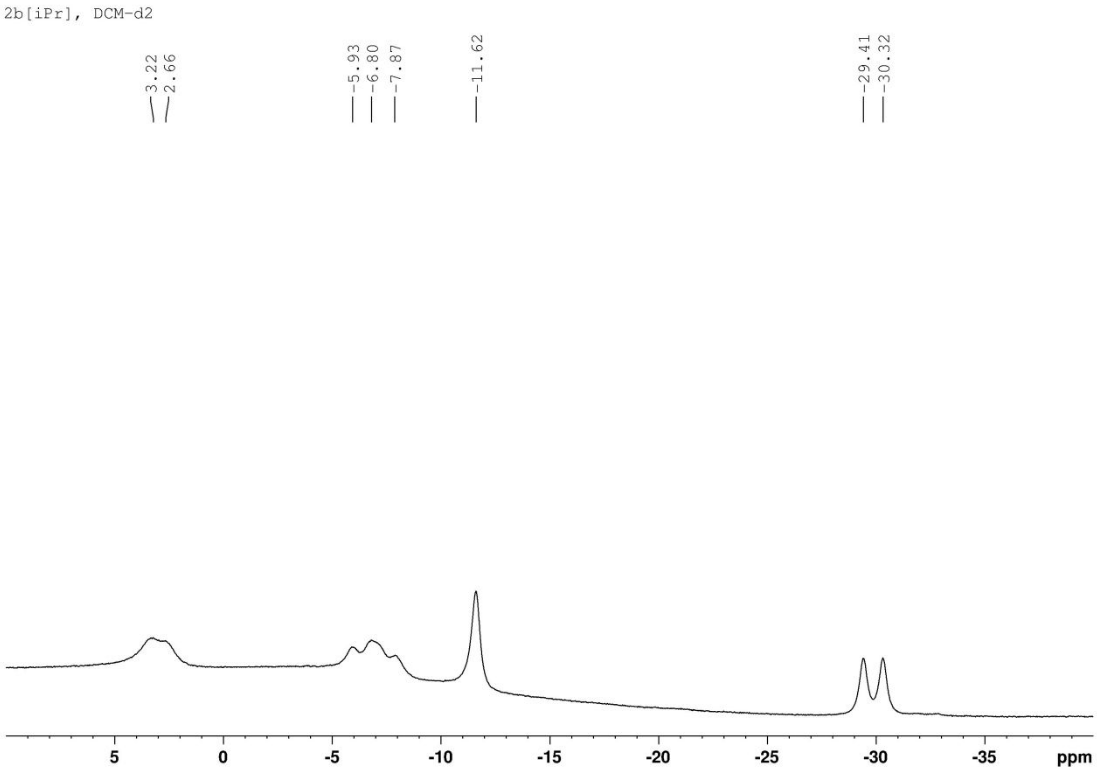

Figure S51. The ^{11}B NMR spectrum of **2b^{iPr}**.

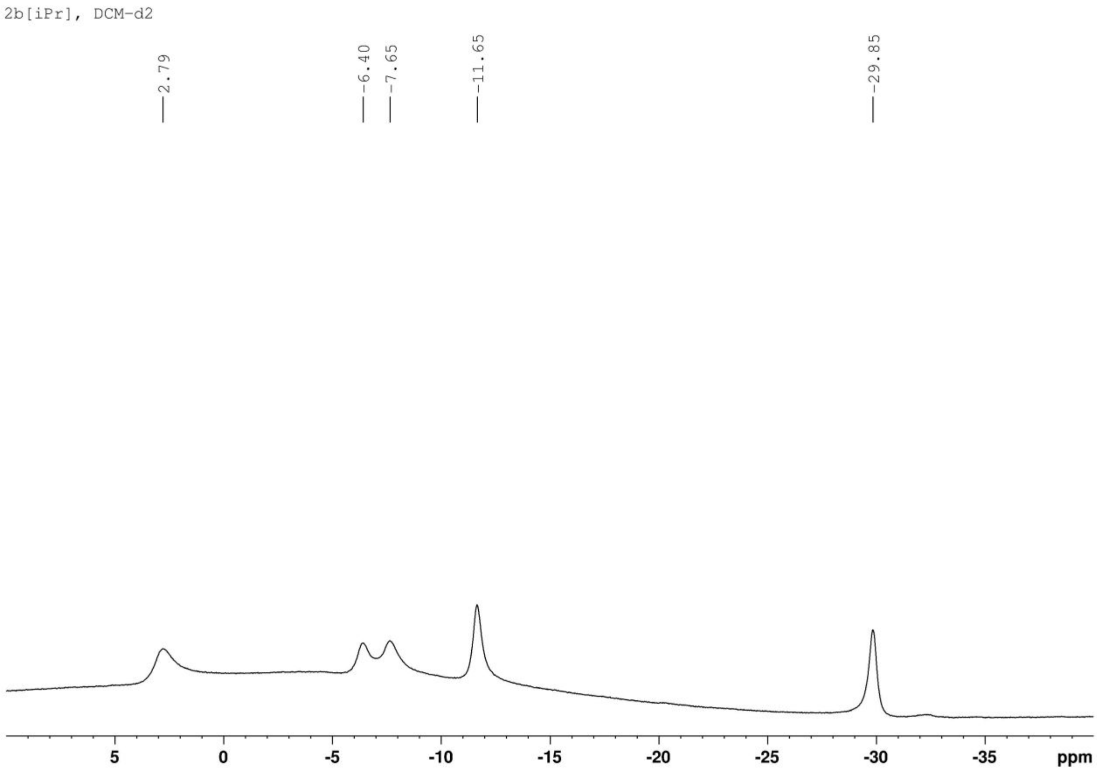

Figure S52. The $^{11}B\{^{1}H\}$ NMR spectrum of **2b^{iPr}**.

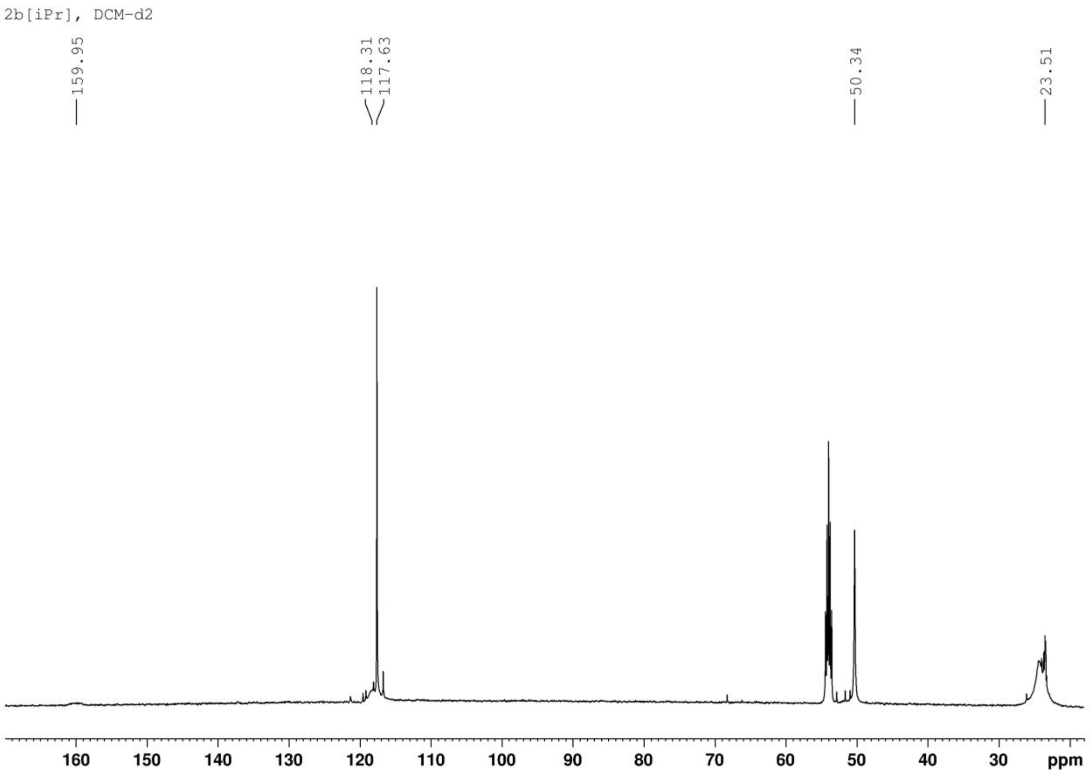

Figure S53. The $^{13}C\{^{1}H\}$ NMR spectrum of **2b**^{iPr}.

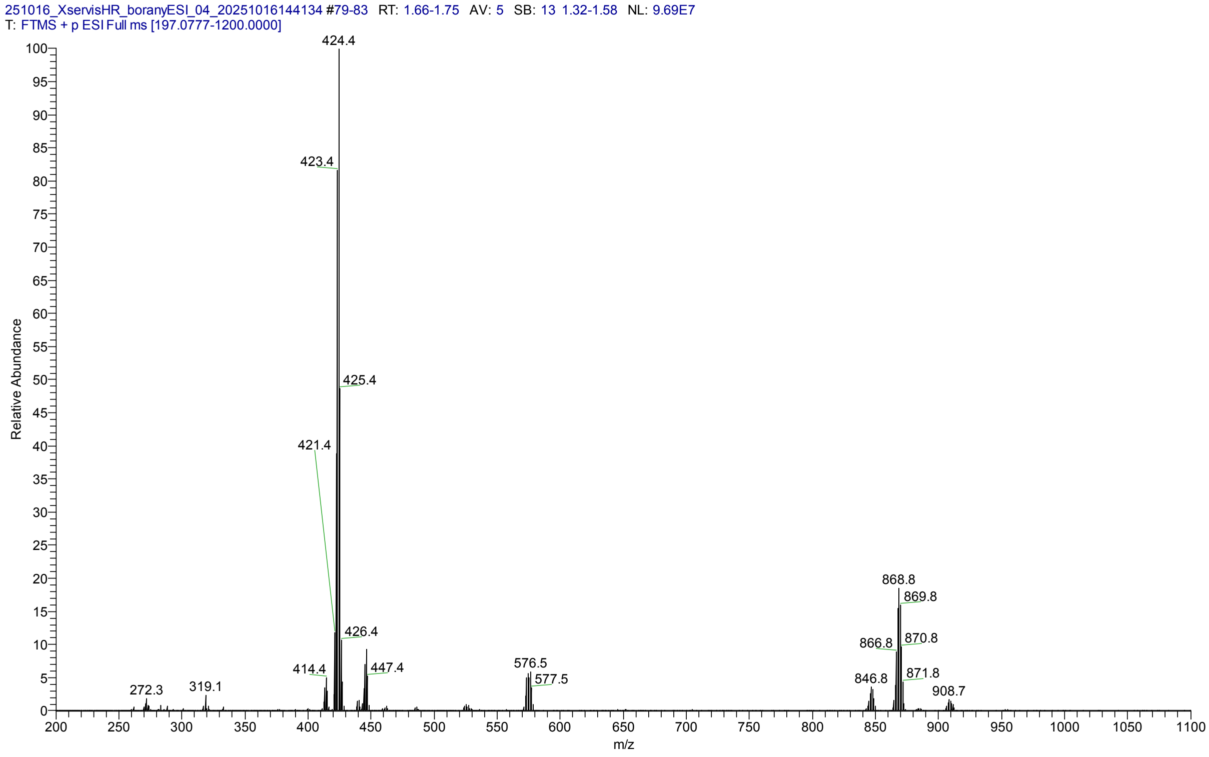

Figure S54. Mass spectrum of positively charged ions (ESI+, Orbitrap) for **2b**^{iPr}.

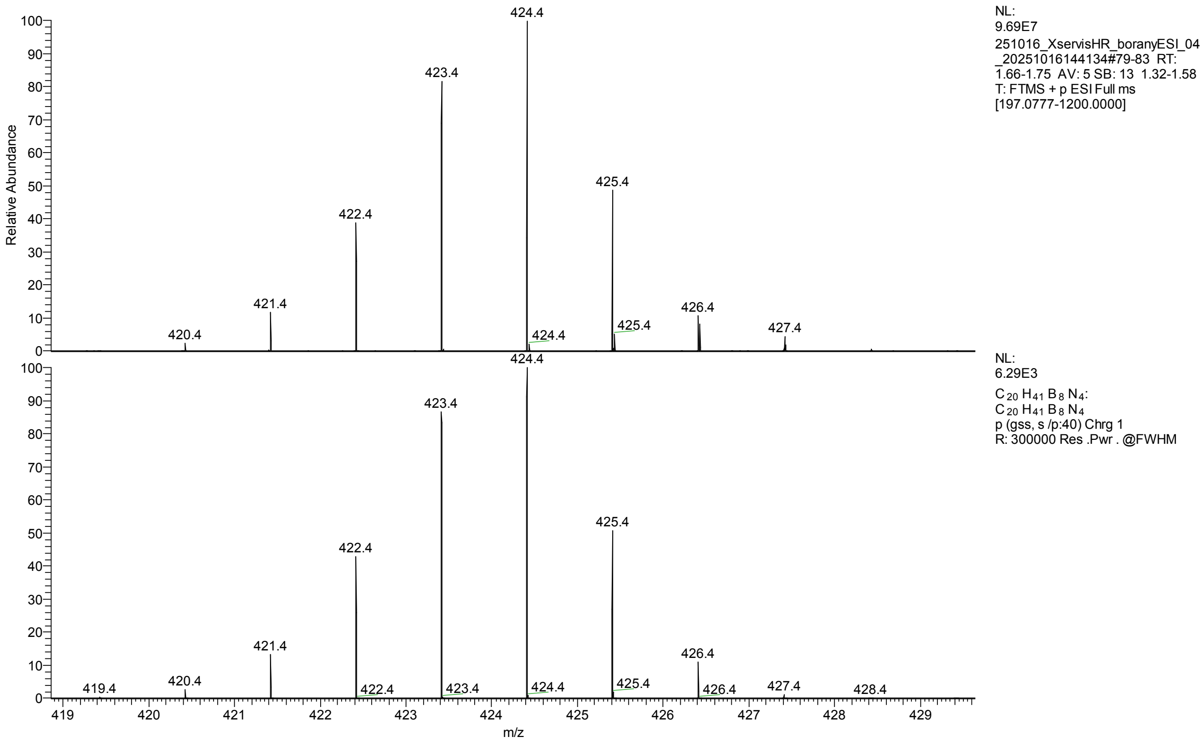

Figure S55. Spectrum of positively charged ions (ESI+, Orbitrap @ R=500,000) for **2b^{iPr}** enlarged in the protonated molecule region (top) and simulated spectrum (bottom).

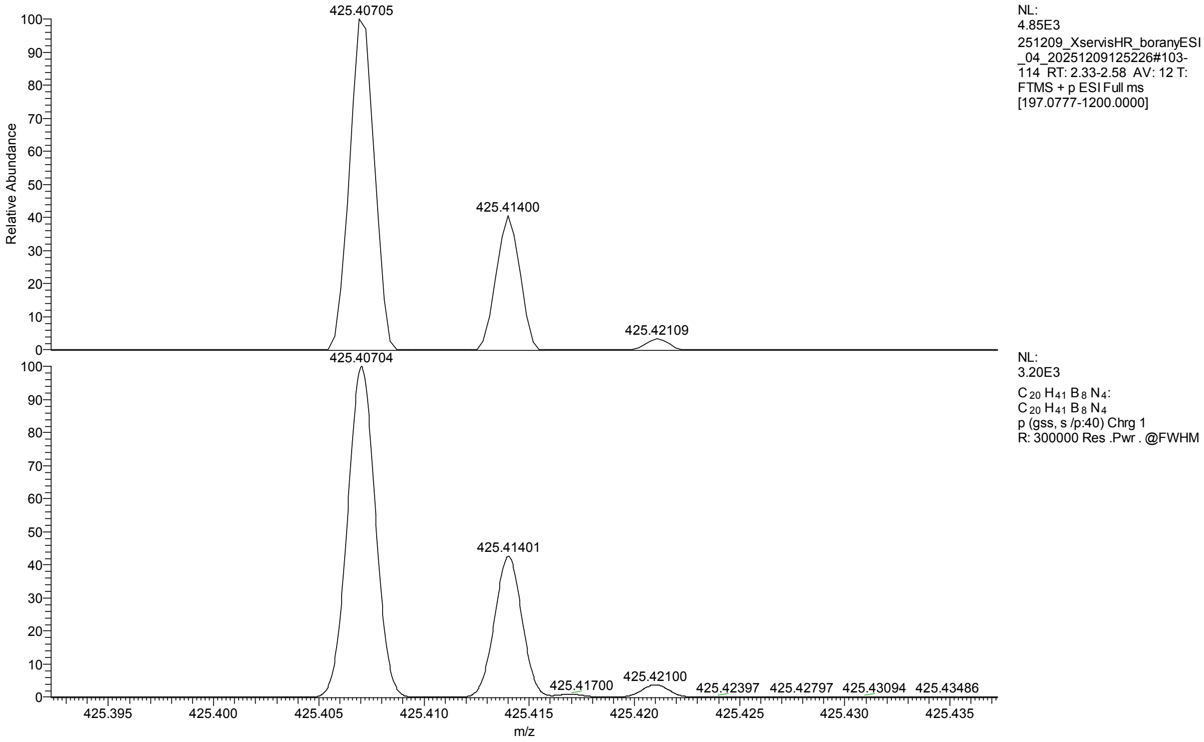

Figure S56. Spectrum of positively charged ions (ESI+, Orbitrap @ R=500,000) for **2b^{iPr}** enlarged in the monoisotopic peak region (top) and simulated spectrum (bottom). Theoretical mass for $C_{20}H_{41}N_4B_8^+$: *m/z* 425.40702; experimental mass: *m/z* 425.40705; mass error 0.06 ppm.

Spectroscopic characterization of 2c^{iPr}.

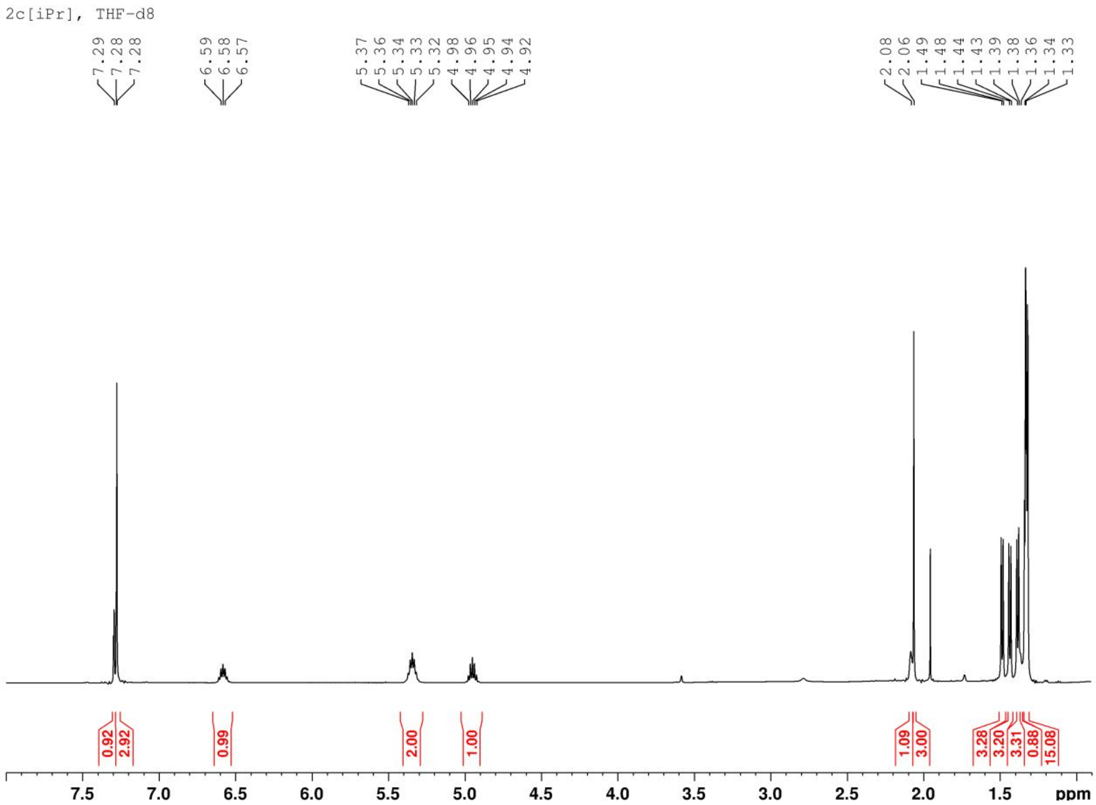

Figure S57. The ^{1}H NMR spectrum of **2c^{iPr}**.

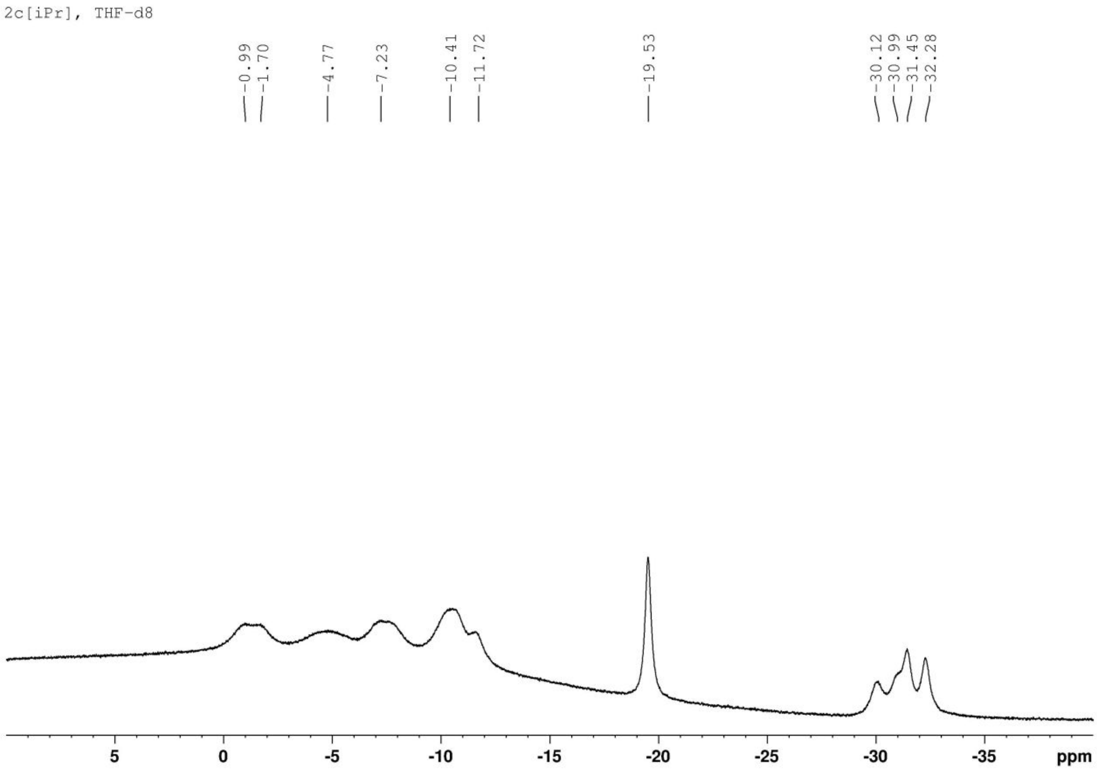

Figure S58. The ^{11}B NMR spectrum of **2c^{iPr}**.

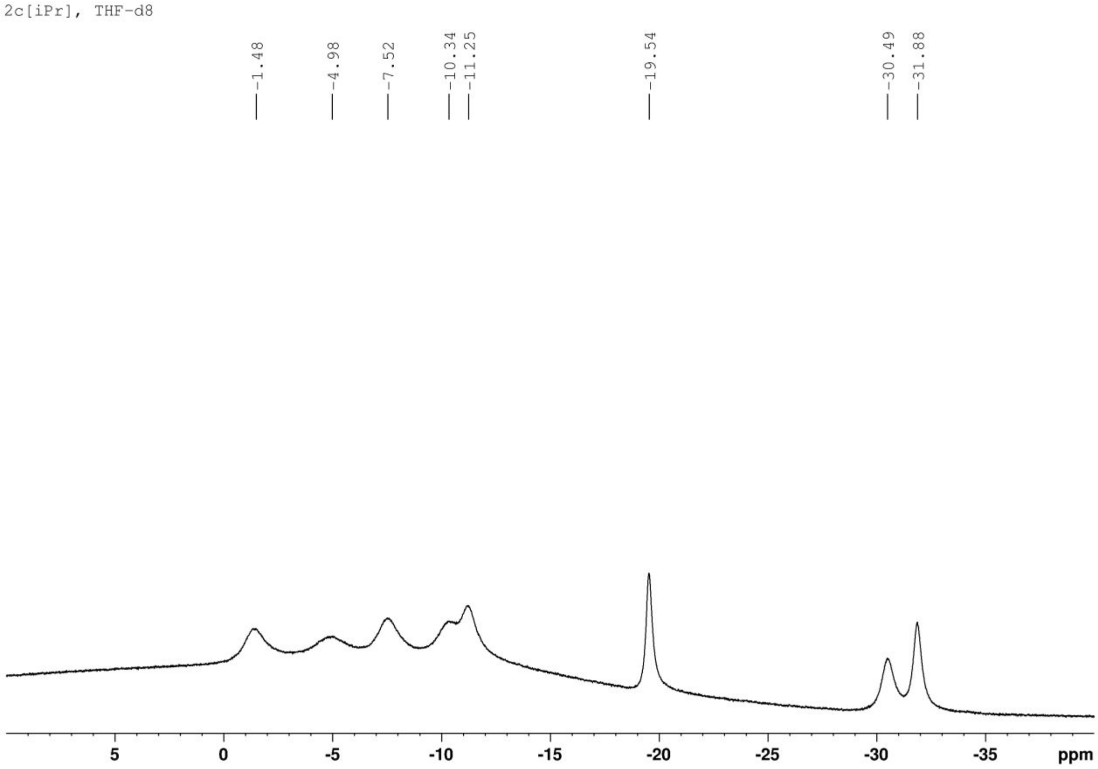

Figure S59. The $^{11}B\{^{1}H\}$ NMR spectrum of $\mathbf{2c}^{iPr}$.

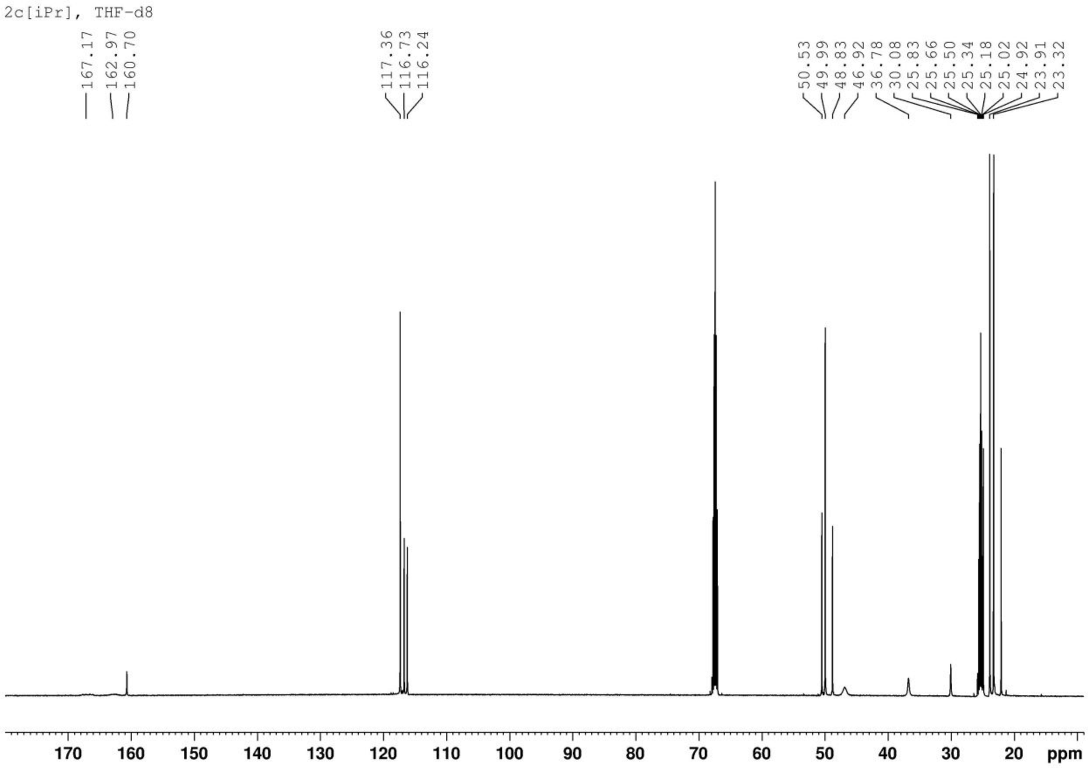

Figure S60. The $^{13}C\{^{1}H\}$ NMR spectrum of $\mathbf{2c}^{iPr}$.

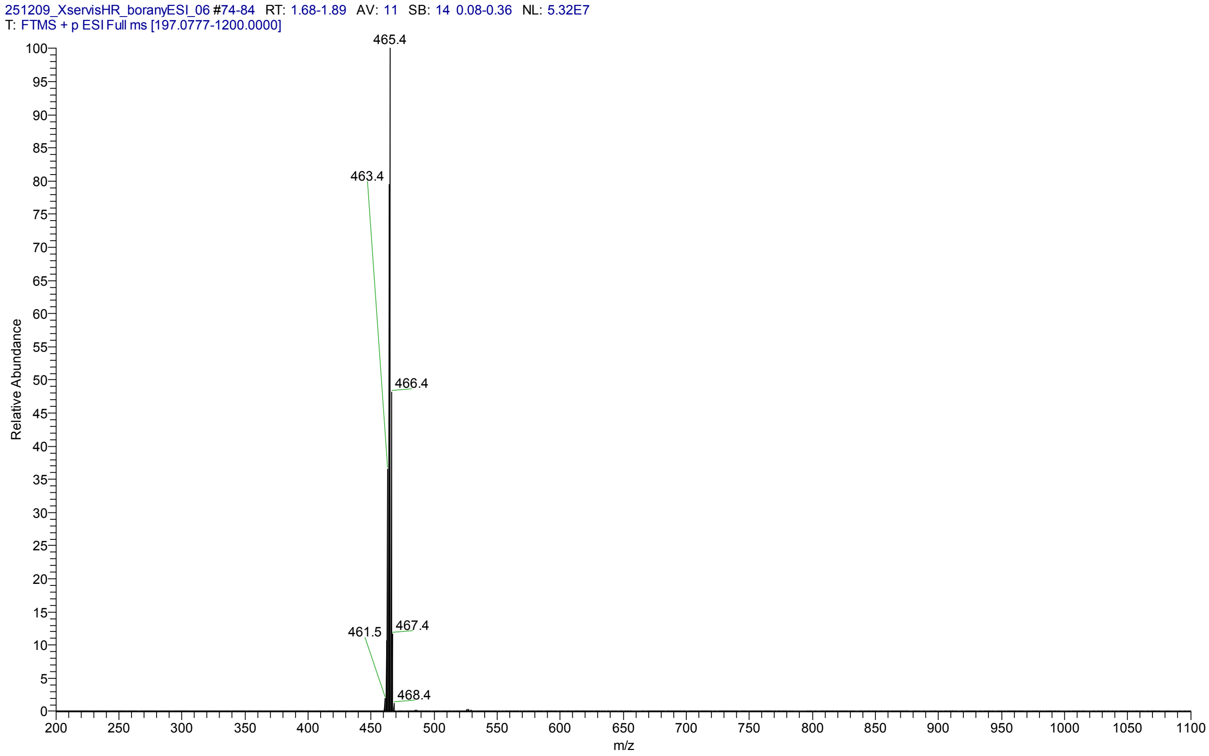

Figure S61. Mass spectrum of positively charged ions (ESI+, Orbitrap) for **2c^{iPr}**.

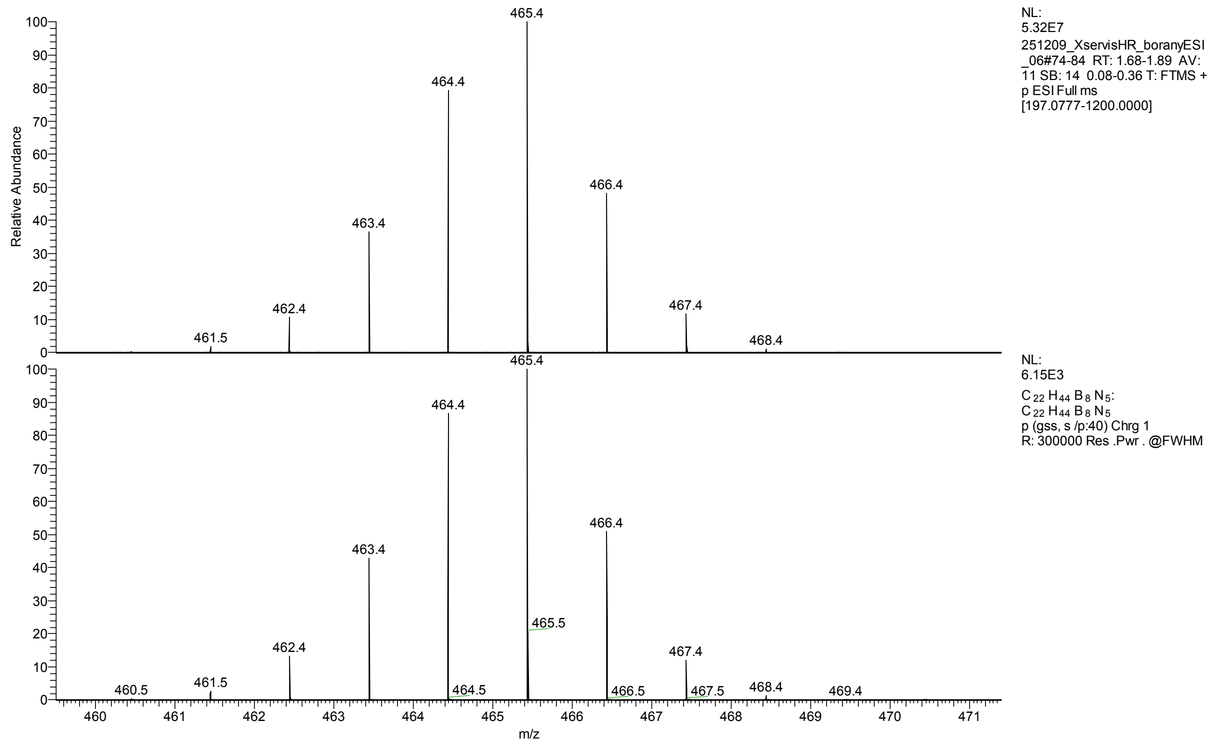

Figure S62. Spectrum of positively charged ions (ESI+, Orbitrap @ R=500,000) for **2c^{iPr}** enlarged in the protonated molecule region (top) and simulated spectrum (bottom).

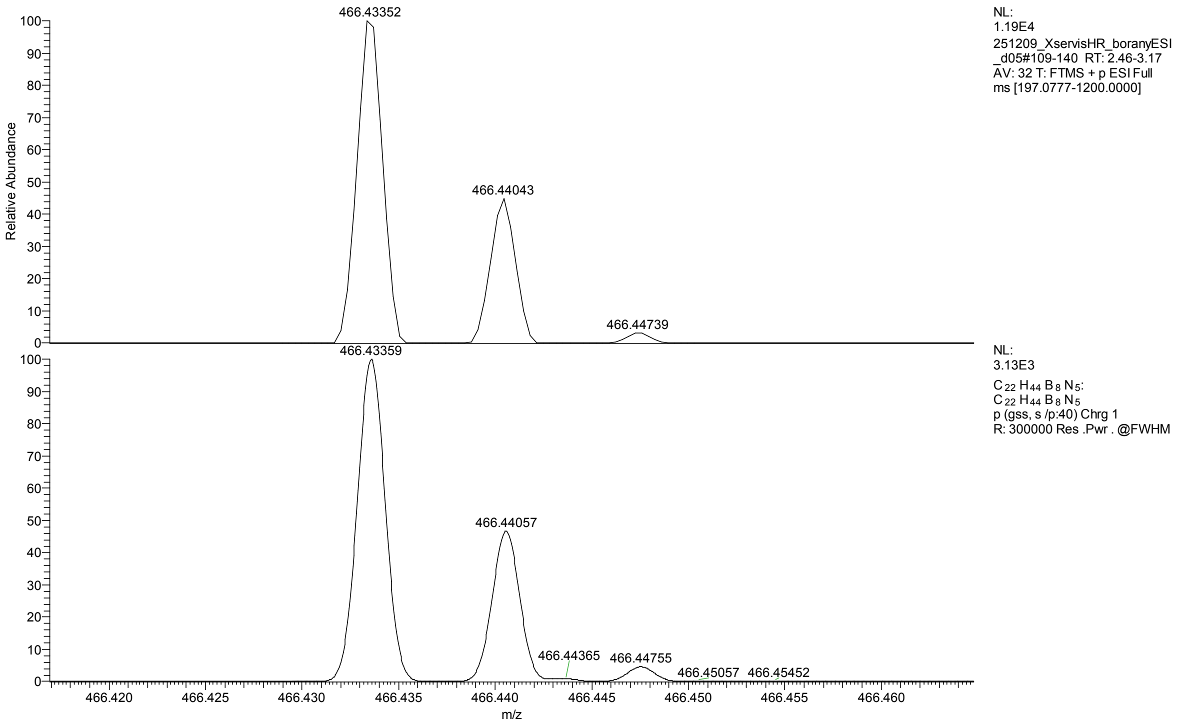

Figure S63. Spectrum of positively charged ions (ESI+, Orbitrap @ R=500,000) for **2c*iPr*** enlarged in the monoisotopic peak region (top) and simulated spectrum (bottom). Theoretical mass for $C_{22}H_{44}N_5B_8^+$: *m/z* 466.43357; experimental mass: *m/z* 466.43352; mass error -0.09 ppm.

Spectroscopic characterization of [3a*iPr*]Cl.

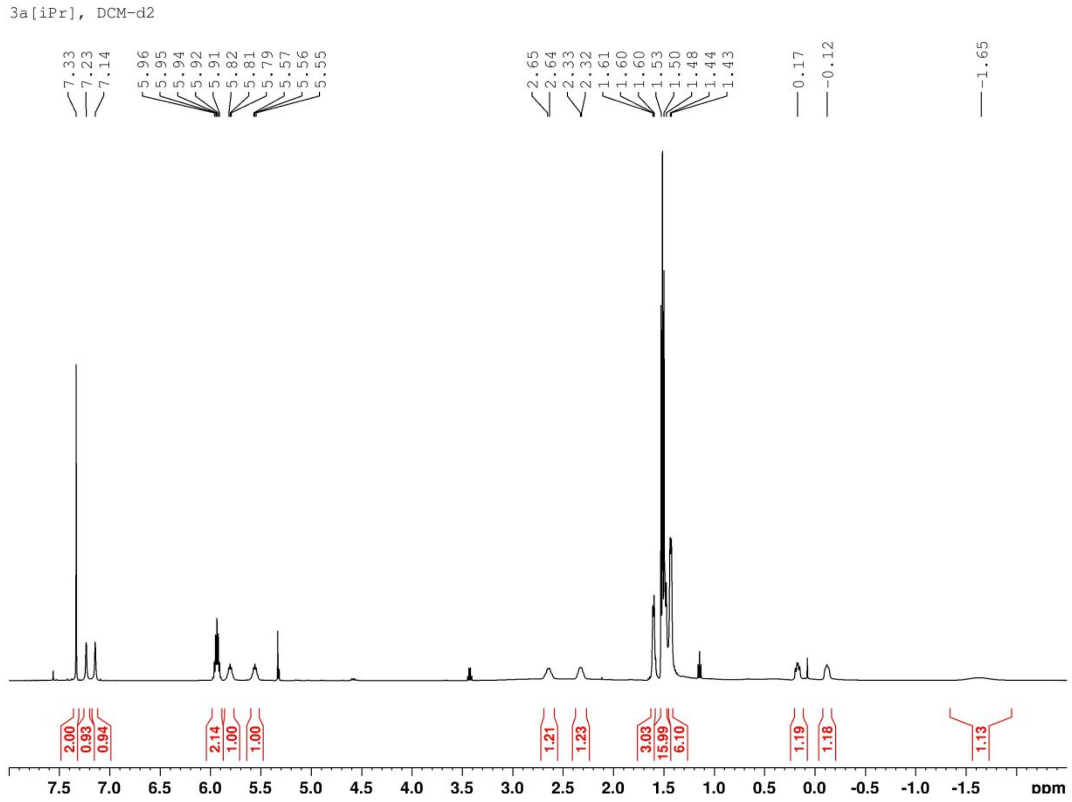

Figure S64. The ^{1}H NMR spectrum of **[3a^{iPr}]Cl**.

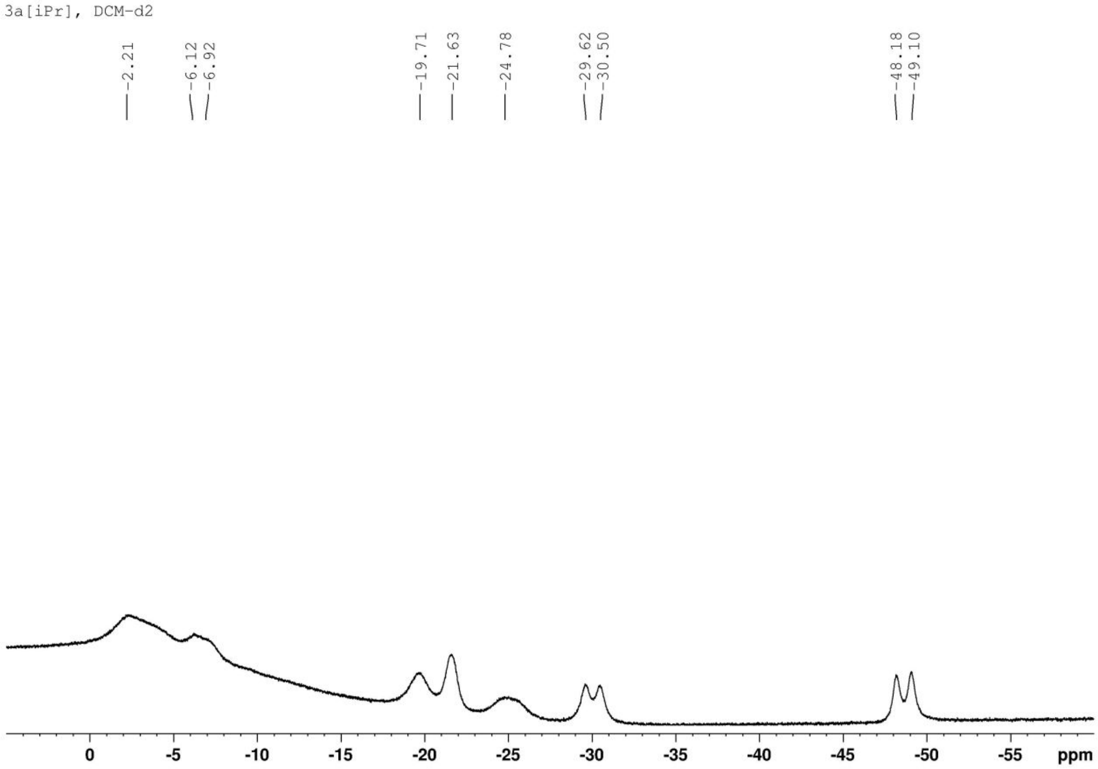

Figure S65. The ^{11}B NMR spectrum of **[3a^{iPr}]Cl**.

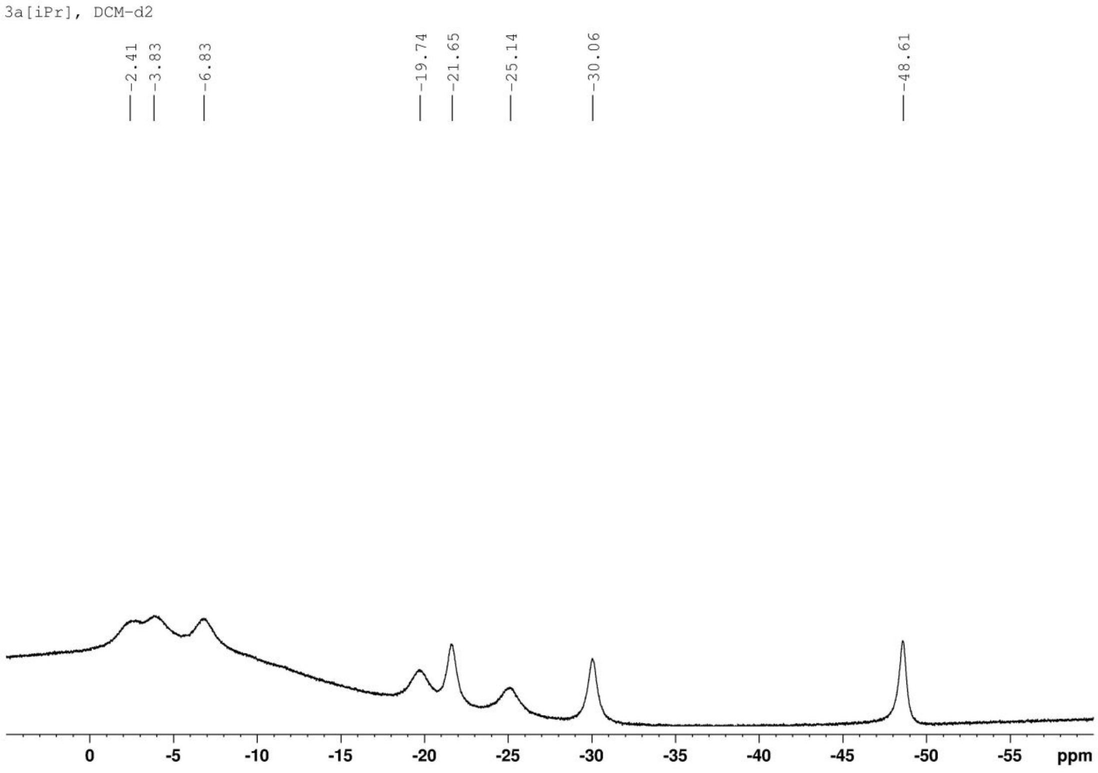

Figure S66. The ^{11}B{^{1}H} NMR spectrum of **[3a^{iPr}]Cl**.

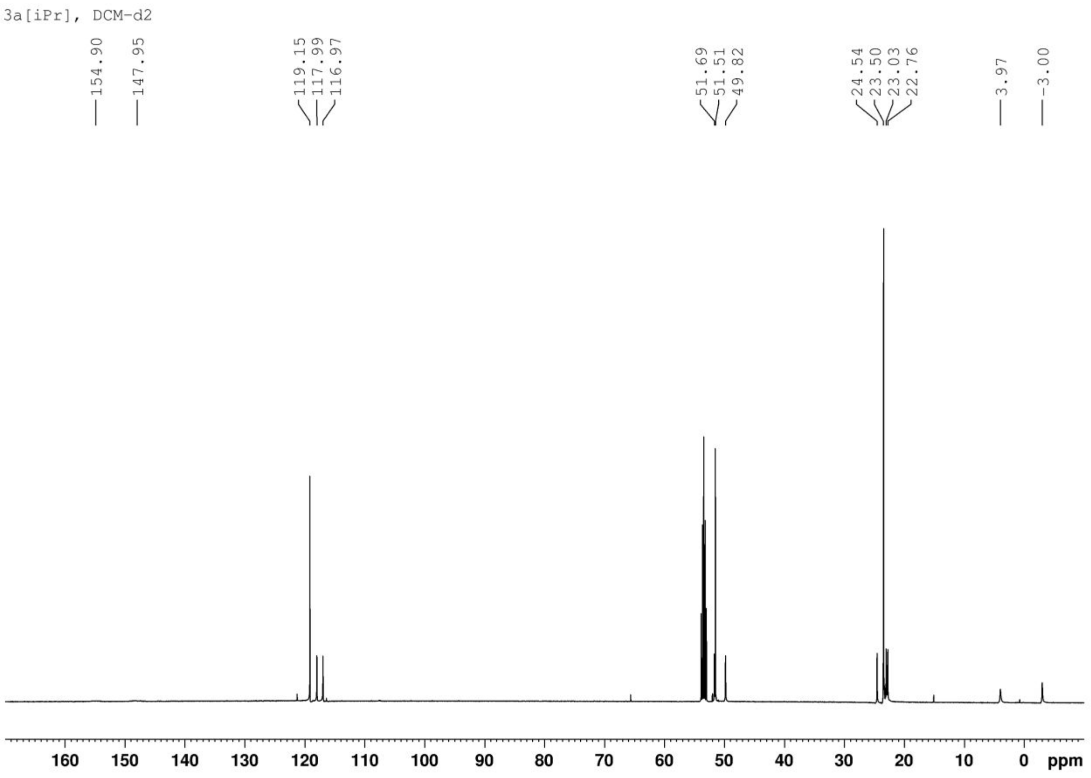

Figure S67. The $^{13}C\{^{1}H\}$ NMR spectrum of **[3a^{iPr}]Cl**.

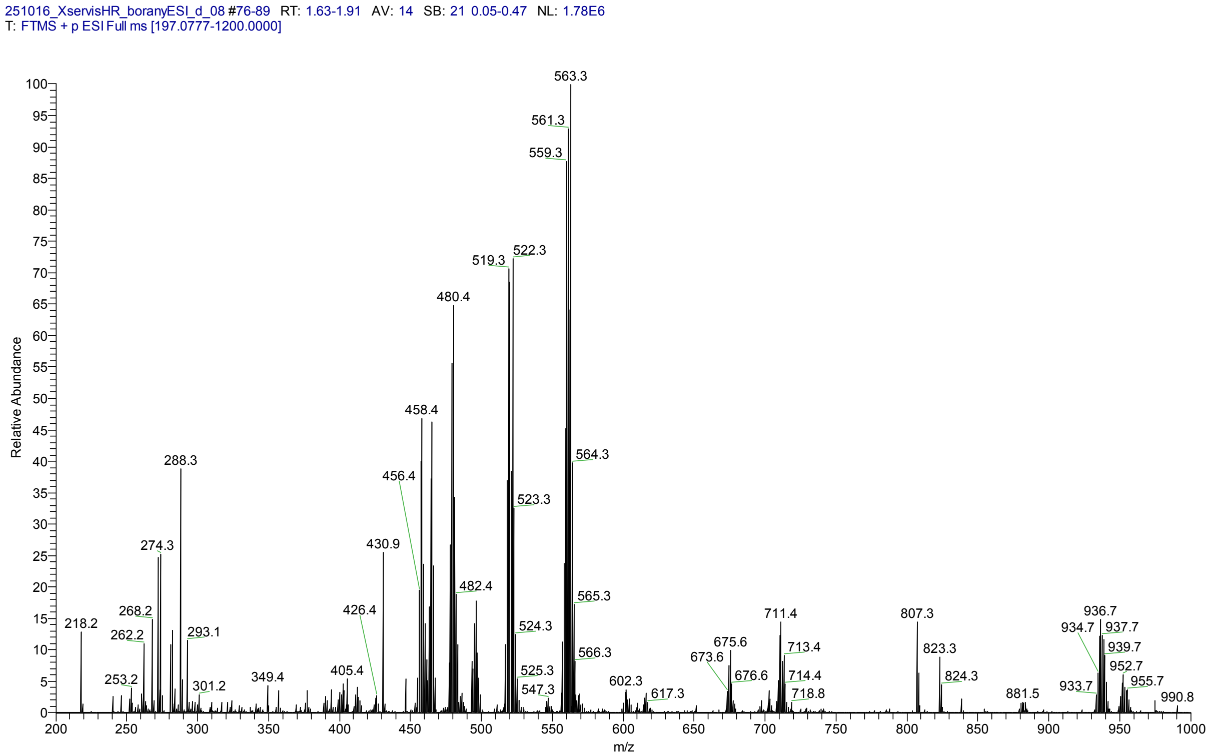

Figure S68. Mass spectrum of positively charged ions (ESI+, Orbitrap) for **[3a^{iPr}]Cl**.

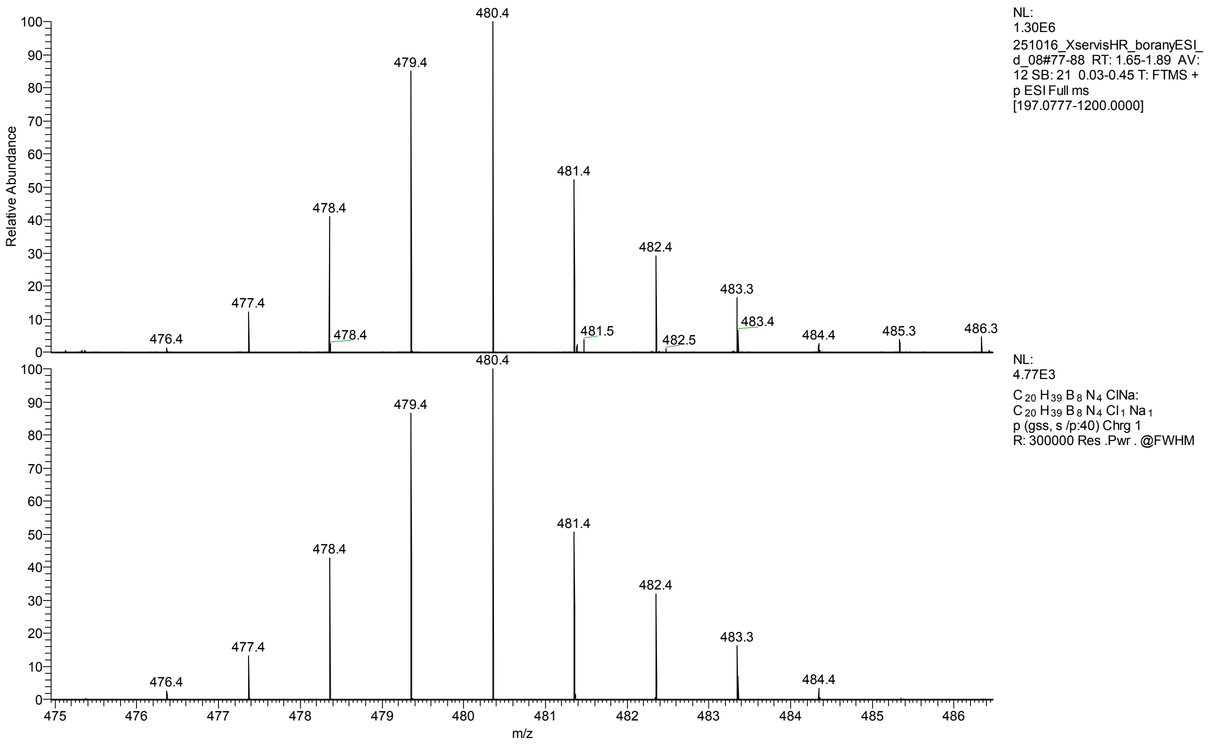

Figure S69. Spectrum of positively charged ions (ESI+, Orbitrap @ R=500,000) for **[3a^{iPr}]Cl** enlarged in the sodium adduct region (top) and simulated spectrum (bottom).

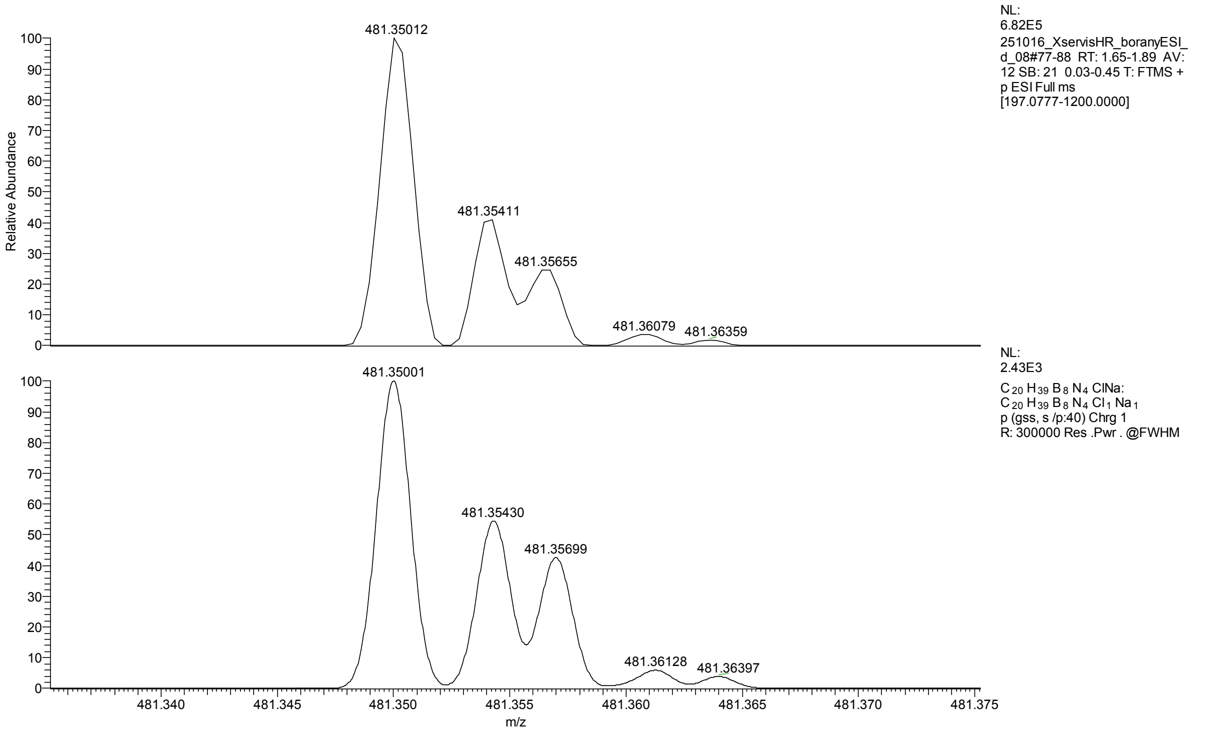

Figure S70. Spectrum of positively charged ions (ESI+, Orbitrap @ R=500,000) for **[3a^{iPr}]Cl** enlarged in the monoisotopic peak region (top) and simulated spectrum (bottom). Theoretical mass for $C_{20}H_{39}N_4B_8ClNa^+$: *m/z* 481.34999; experimental mass: *m/z* 481.35012; mass error 0.30 ppm.

Spectroscopic characterization of [3b^{iPr}]Cl.

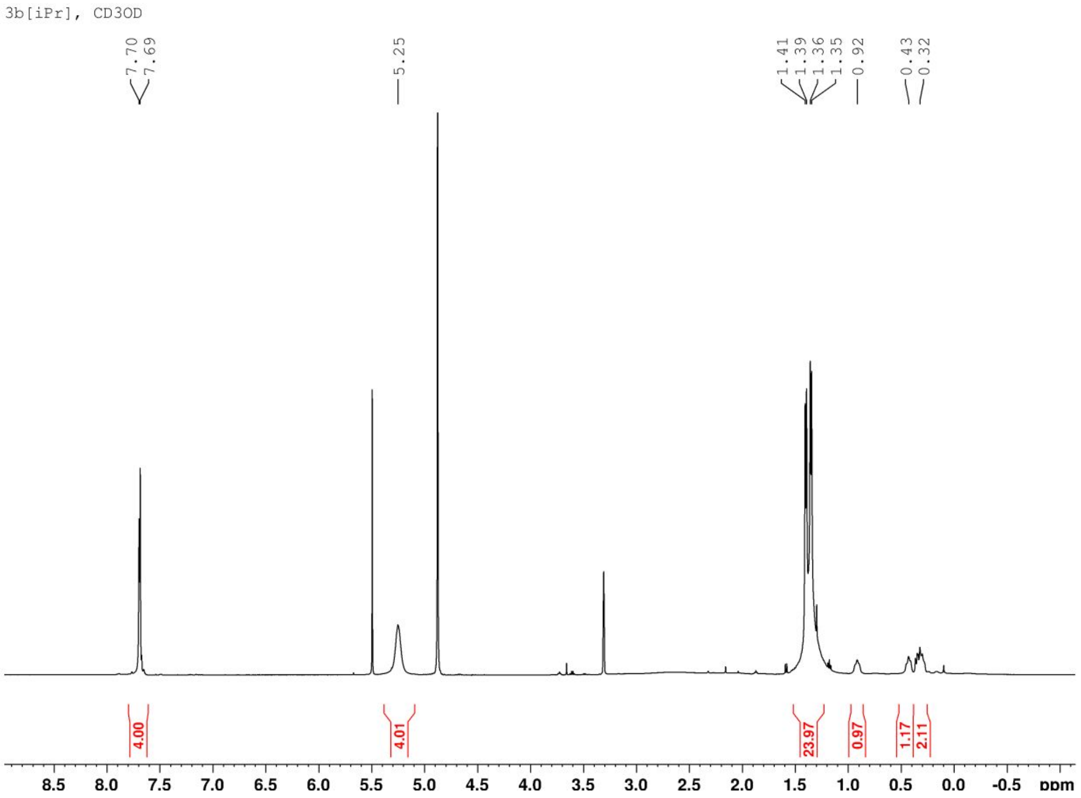

Figure S71. The ^{1}H NMR spectrum of **[3b^{iPr}]Cl**.

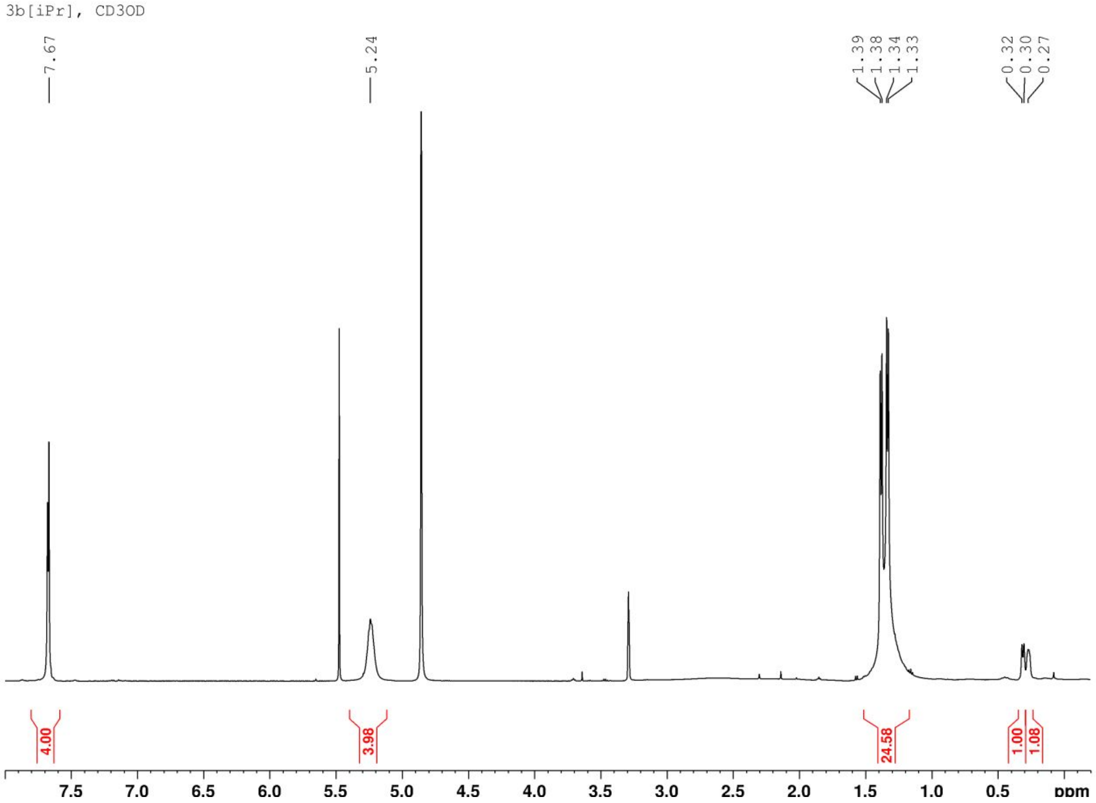

Figure S72. The ^{1}H NMR spectrum of **[3b^{iPr}]Cl** isolated from the reaction of **2b^{iPr}** and DCl.

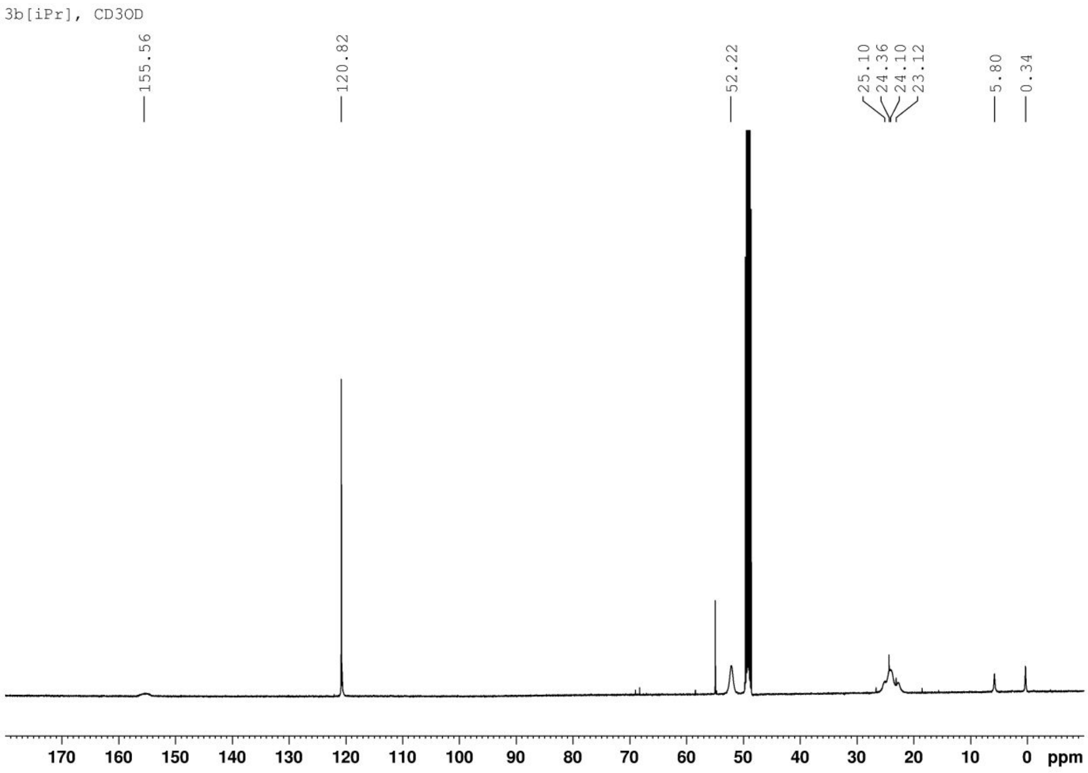

Figure S73. The $^{13}C\{^{1}H\}$ NMR spectrum of **[3b^{iPr}]Cl**.

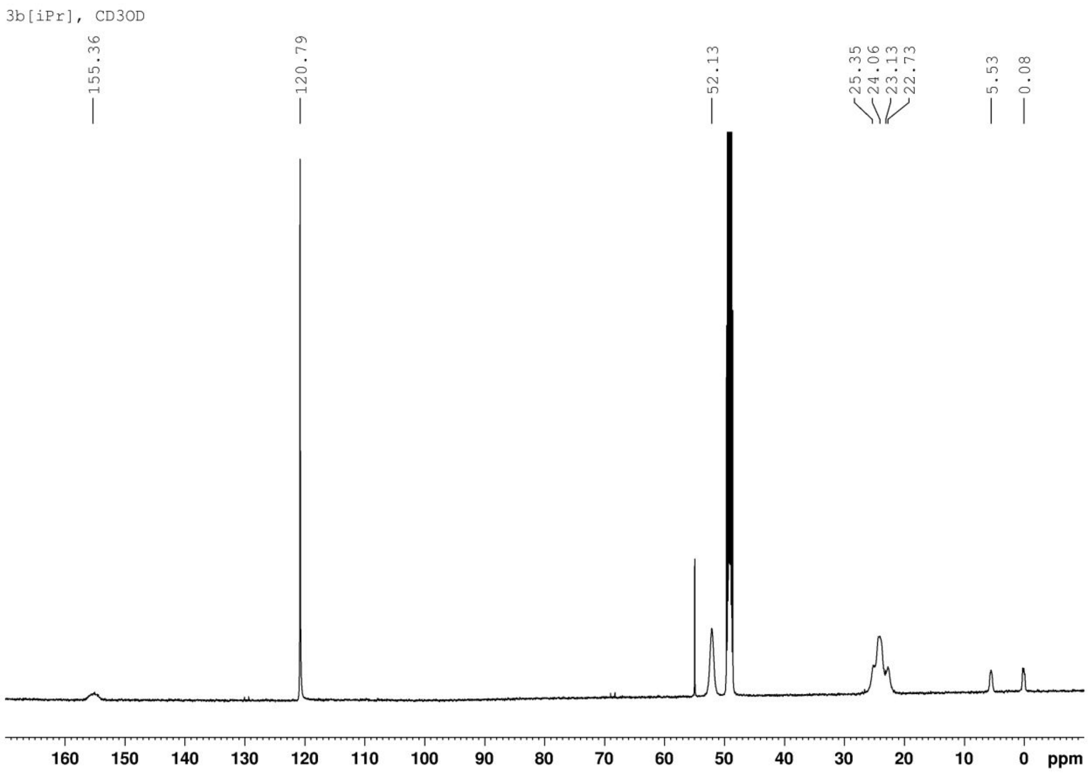

Figure S74. The $^{13}C\{^{1}H\}$ NMR spectrum of **[3b^{iPr}]Cl** isolated from the reaction of **2b^{iPr}** and DCl.

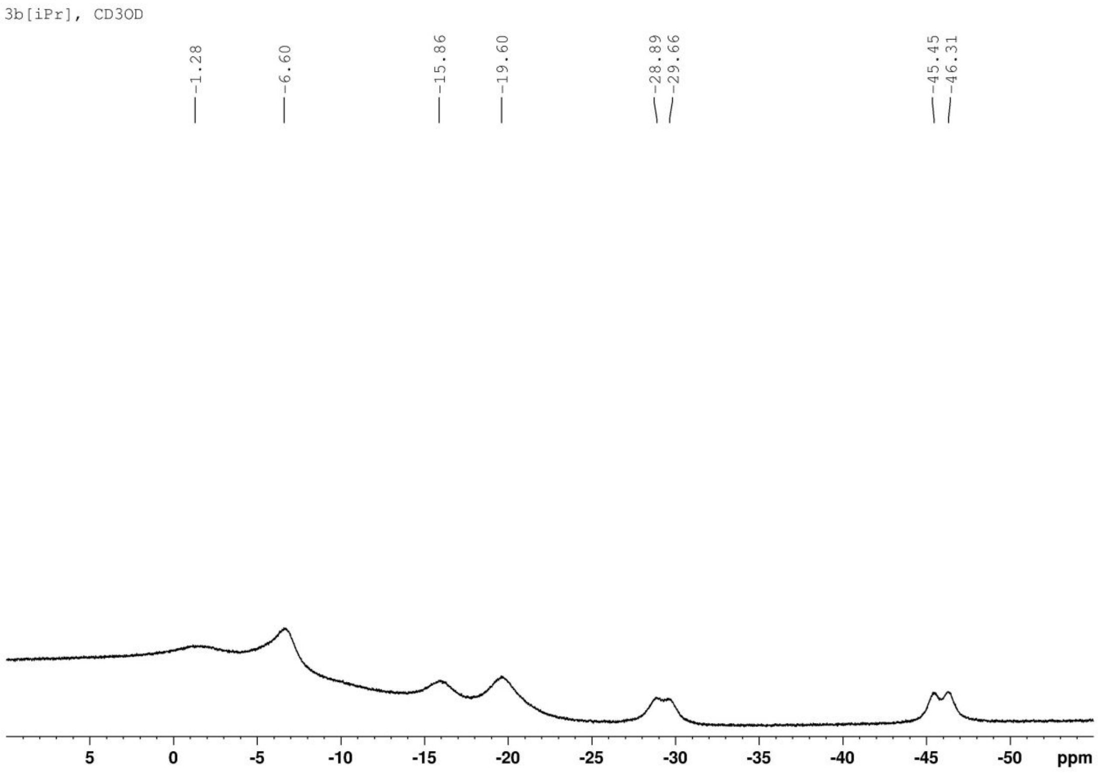

Figure S75. The ^{11}B NMR spectrum of **[3b^{iPr}]Cl**.

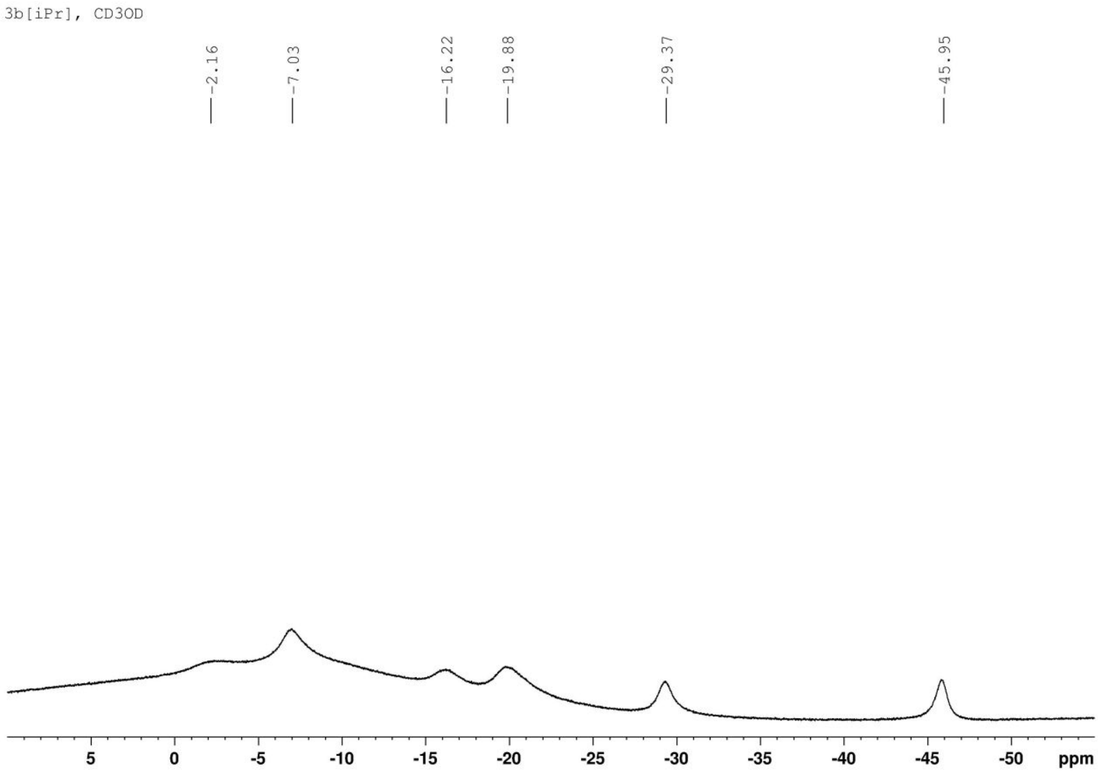

Figure S76. The $^{11}B\{^1H\}$ NMR spectrum of **[3b^{iPr}]Cl**.

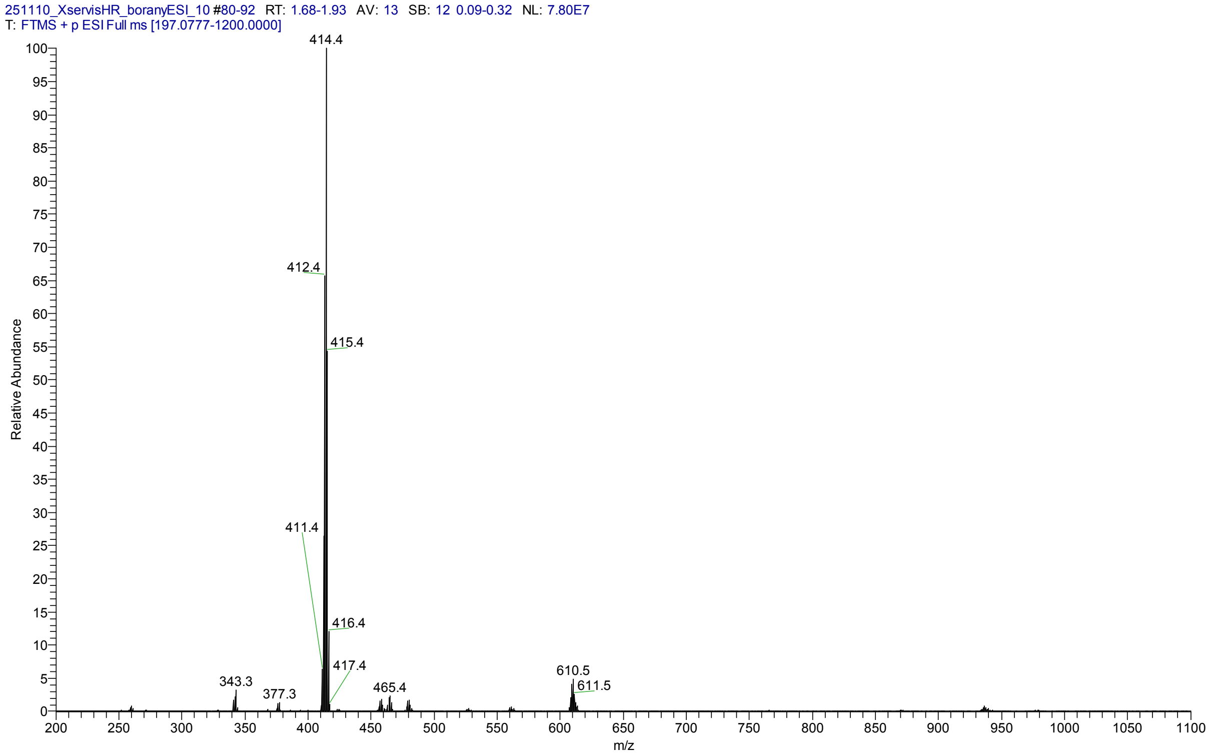

Figure S77. Mass spectrum of positively charged ions (ESI+, Orbitrap) for **[3b^{iPr}]Cl**.

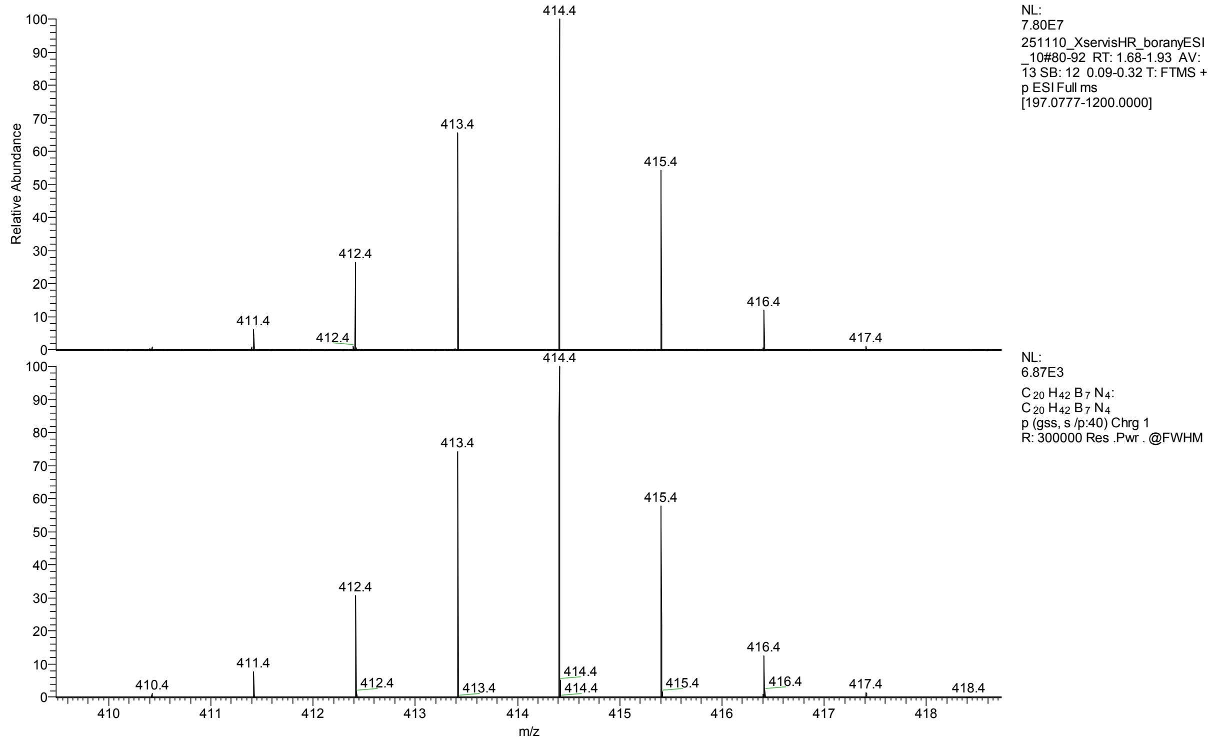

Figure S78. Spectrum of positively charged ions (ESI+, Orbitrap @ R=500,000) for **[3b^{iPr}]Cl** enlarged in the sodium adduct region (top) and simulated spectrum (bottom).

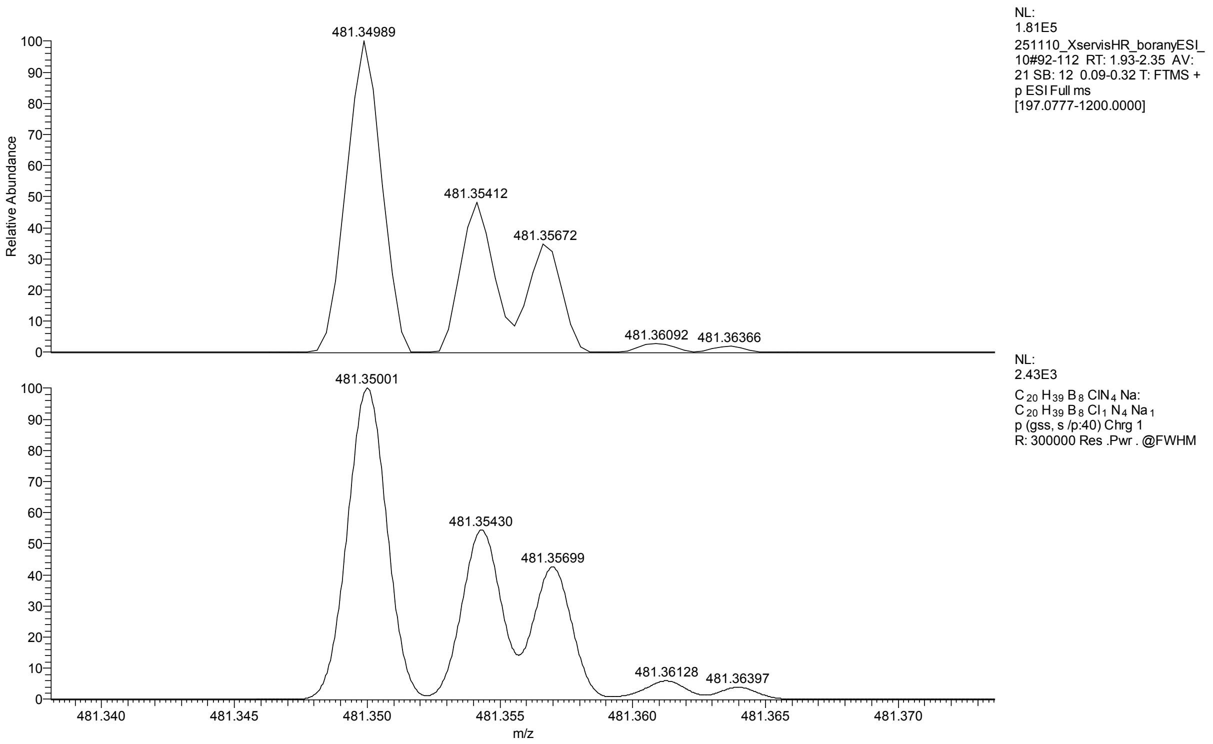

Figure S79. Spectrum of positively charged ions (ESI+, Orbitrap @ R=500,000) for **[3b^{iPr}]Cl** enlarged in the monoisotopic peak region (top) and simulated spectrum (bottom). Theoretical mass for $C_{20}H_{39}N_4B_8ClNa^+$: *m/z* 481.34999; experimental mass: *m/z* 481.34989; mass error -0.20 ppm.

Spectroscopic characterization of [3c^{iPr}][HCl$_2$].

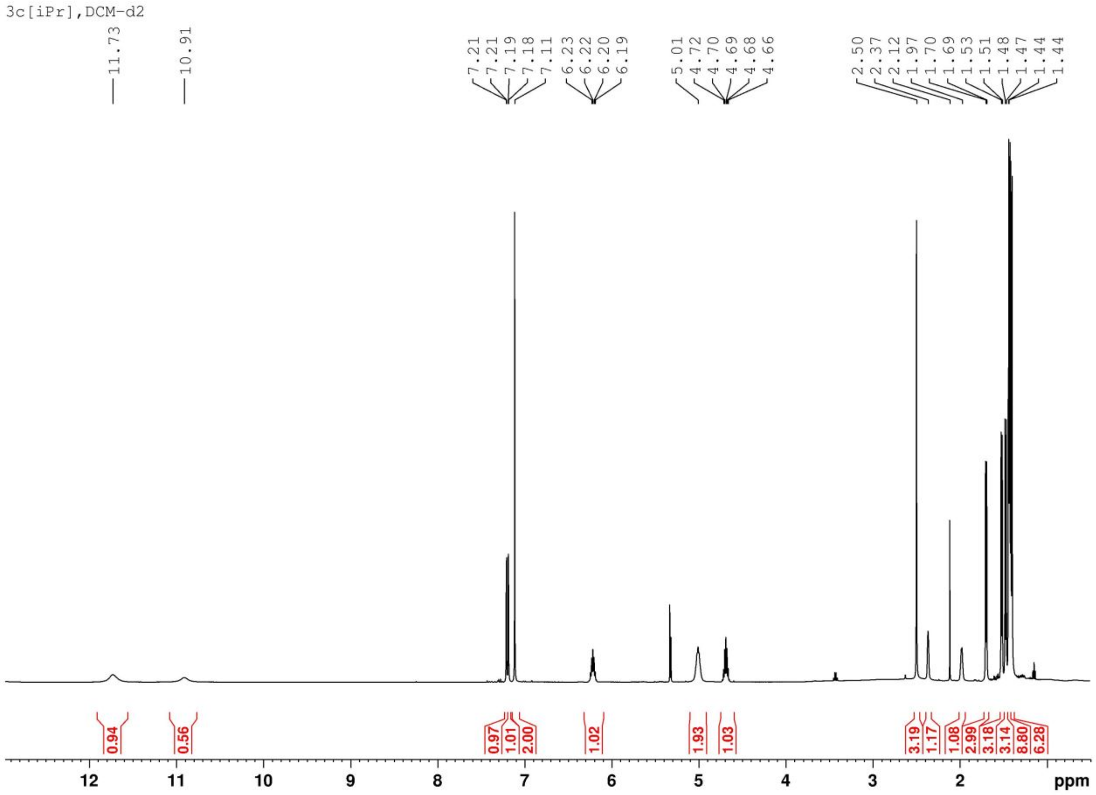

Figure S80. The ^{1}H NMR spectrum of **[3c^{iPr}][HCl$_2$]**.

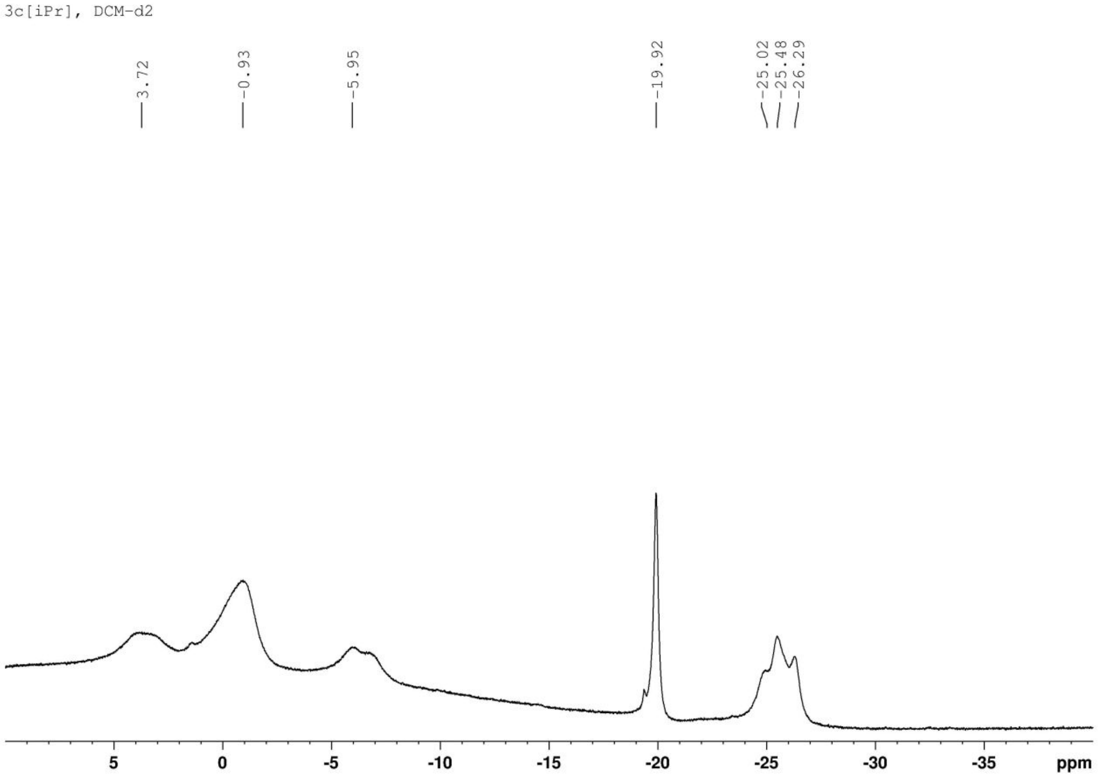

Figure S80. The ^{11}B NMR spectrum of **[3c^{iPr}][HCl$_2$]**.

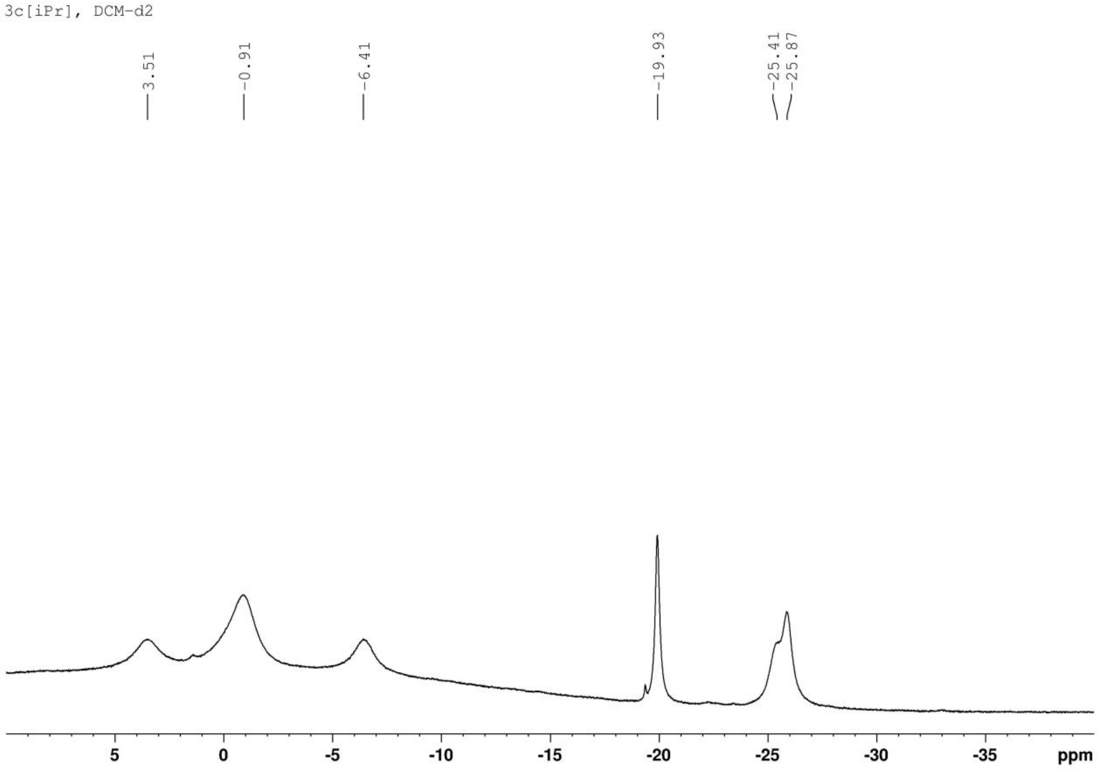

Figure S81. The ^{11}B{^{1}H} NMR spectrum of **[3c^{iPr}][HCl$_2$]**.

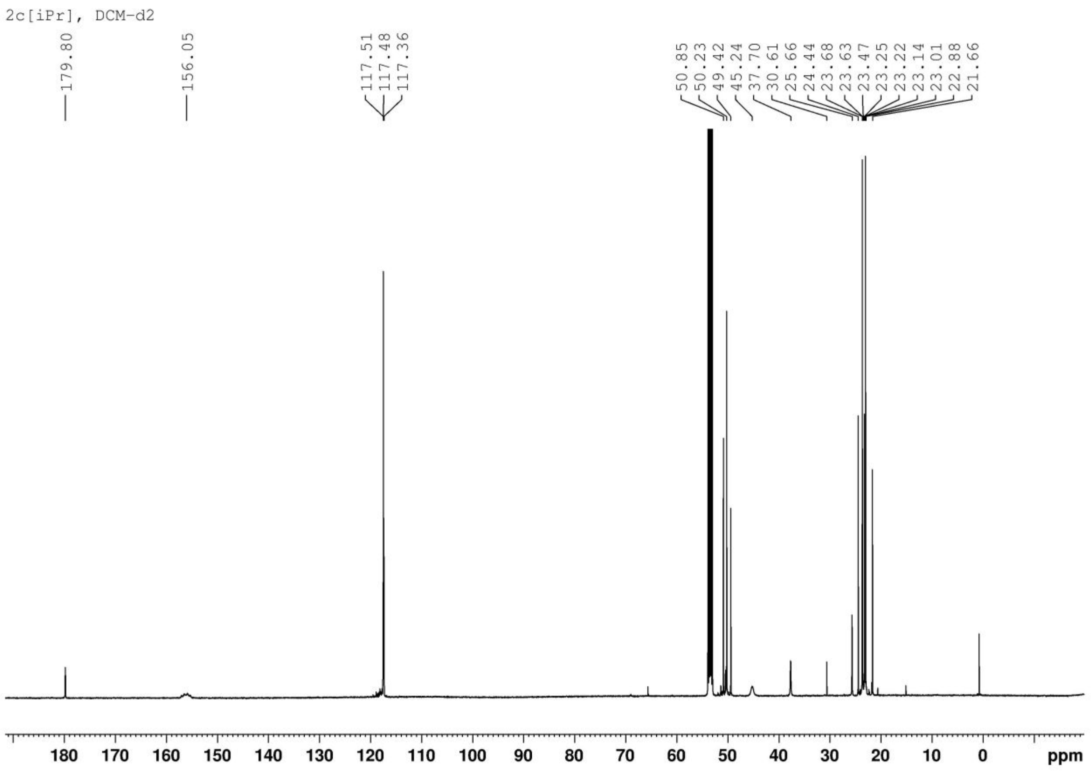

Figure S82. The $^{13}C\{^{1}H\}$ NMR spectrum of **[3c^{iPr}][HCl$_2$]**.

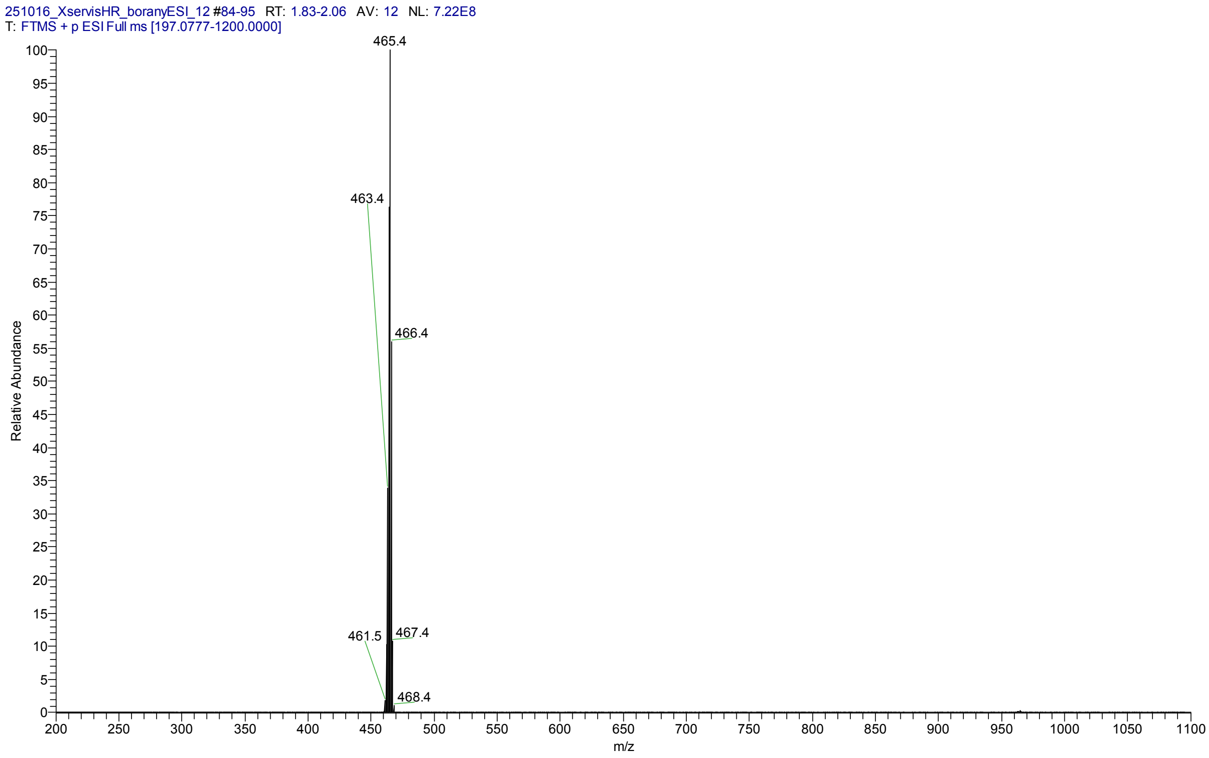

Figure S83. Mass spectrum of positively charged ions (ESI+, Orbitrap) for **[3c^{iPr}][HCl$_2$]**.

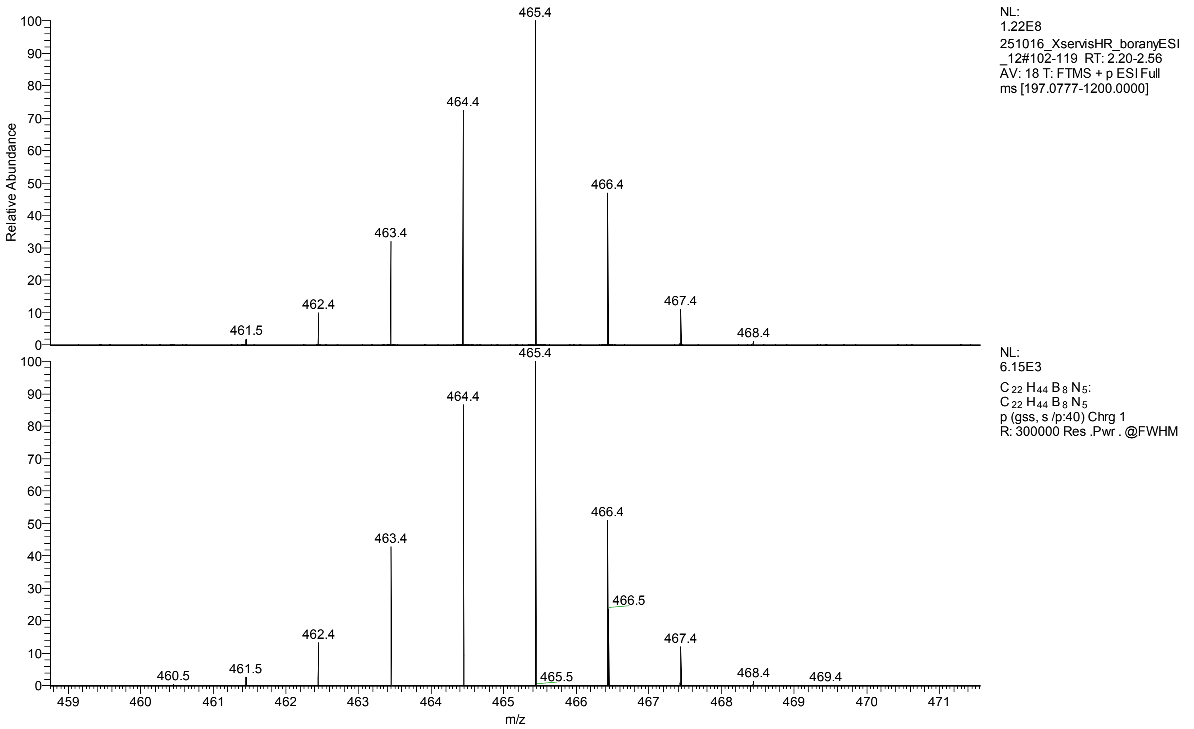

Figure S84. Spectrum of positively charged ions (ESI+, Orbitrap @ R=500,000) for **[3c^{iPr}][HCl$_2$]** enlarged in the protonated molecule region (top) and simulated spectrum (bottom).

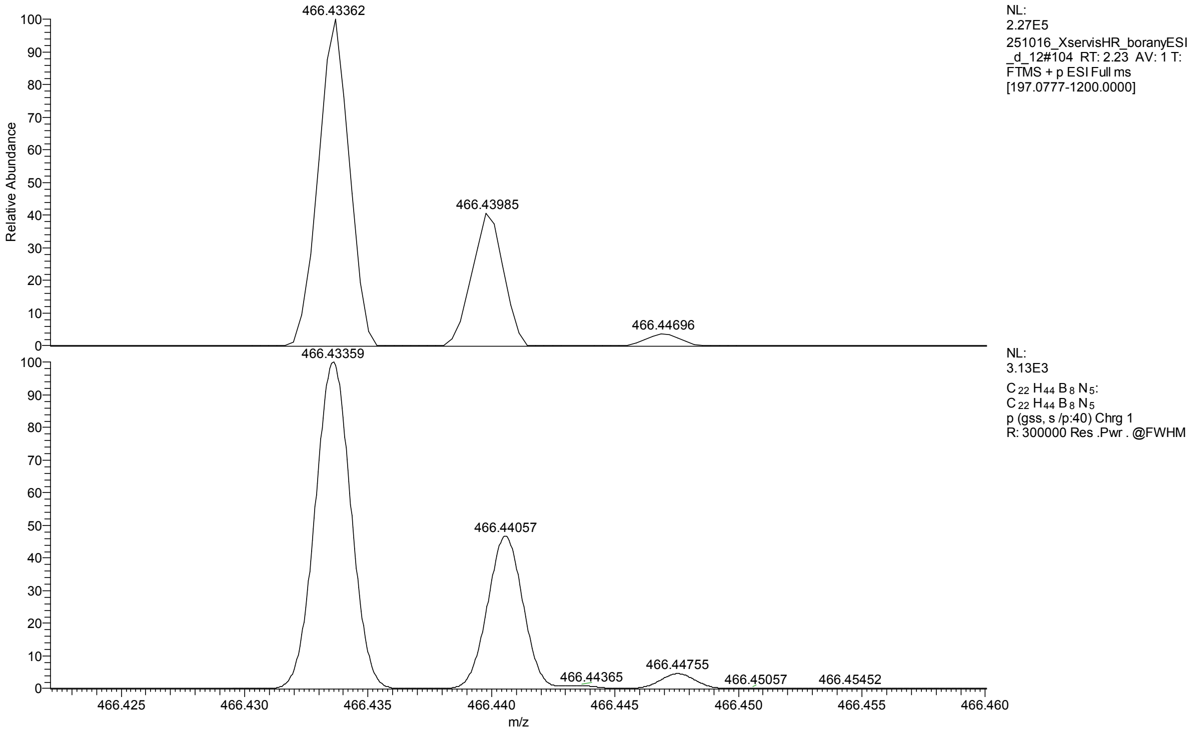

Figure S85. Spectrum of positively charged ions (ESI+, Orbitrap @ R=500,000) for **[3c^{iPr}][HCl$_2$]** enlarged in the monoisotopic peak region (top) and simulated spectrum (bottom). Theoretical mass for $C_{22}H_{44}N_5B_8^+$: *m/z* 466.43357; experimental mass: *m/z* 466.43362; mass error 0.12 ppm.

NMR Data

^{11}B NMR

The ^{11}B NMR data of the prepared compounds are summarized in Table S1 and their correlation diagram (Figure S86) shows no significant deviations from the patterns found in analogical compounds (*arachno*-6,9-$C_2B_8H_{14}$, ***m*-2** and **1^{Dipp}**)[26,32]. The spectra are typically framed by upfield-shifted (–30–(–50) ppm) B1 and B3 and downfield-shifted broaden B2 and B4 signals. The chemical shifts of the vertices from the upper rim can be found in a narrow range (0–(–20) ppm) considering the number of combinations of mutual carbon atoms and carbenes positions. The only exceptions are the vertices B6 and B9 bearing carbene groups in compounds **1^{MIC}**, **1^{iPr}** and **[3a^{Dipp}]$^+$** (–33–(–35) ppm), which is consistent with the spectrum of previously published **1^{Dipp}**.[32]

^{1}H, ^{13}C NMR

The ^{1}H and ^{13}C NMR shifts of the skeletal CH- groups in the spectra of **2a^{Dipp}** ($\delta(^1H)$ = 4.46 ppm; $\delta(^{13}C)$ = 119.0 ppm) are strongly downfield in comparison with **1^{Dipp}** ($\delta(^1H)$ = 0.09 ppm; $\delta(^{13}C)$ = 20.3 ppm)[32], which is consistent with the transfer of carbon atoms. Both types of CH-groups are present in the spectra of **[3a^{Dipp}]$^+$** ($\delta(^1H)$ = 1.17 and 3.88 ppm; $\delta(^{13}C)$ = 44.4 and 107.5 ppm). The ^{11}B NMR spectra have revealed the expected patterns (Table S1), with the most distinct changes in the shifts ($\delta(^{11}B)$ = −33.6 and 10.1 ppm) of the carbene-bearing boron atoms.

The NMR shifts of both carbon and boron skeletal atoms in the spectra of **1^{MIC}** and **2^{MIC}** show slightly changes in comparison with **1^{Dipp}** and **2a^{Dipp}** confirming their expected structure.

The ^{1}H and ^{13}C NMR shifts of the skeletal CH groups of **2a^{iPr}** and **2b^{iPr}** are strongly upfield, resembling **2a^{Dipp}**, whereas the signals of these groups in **2c^{iPr}** have remained in the aliphatic region. This is consistent with the structure, because one of the carbon atoms has not changed its position and the second has increased its coordination number through acetonitrile-molecule addition.

Similar shift of the CH group signals has been observed in the spectra of **[3a^{iPr}]$^+$** and **[3b^{iPr}]$^+$** ($\delta(^{13}C)$ = −0.3–4.9 ppm), which is consistent with the *nido*- → *arachno*- transformation. The CH_2 groups exhibit AX-pattern with the hydrogen atoms oriented inside the six-membered rim being downfield-shifted.

Table S1. Experimental ^{13}C (**bold**) and ^{11}B NMR shifts of the skeletal carbon (**bold**) and boron atoms. Molecular structures are listed below the table and correlation diagram of the ^{11}B NMR shifts in Figure S86.

	1	2	3	4	5	6	7	8	9	10
**1^{Dipp}**	−37.1	−4.8	−52.3	−4.8	**20.3**	−37.1	−8.2	−8.2	−37.1	**20.3**
**1^{iPr}**	−34.0	−1.3	−49.7	−1.3	**---**	−34.0	−6.4	−6.4	−34.0	**---**
**1^{MIC}**	−34.3	−0.8	−48.5	−0.8	**28.3**	−34.3	−6.7	−6.7	−34.3	**28.3**
**$2a^{Dipp}$**	−29.5	−3.9	−29.5	−3.9	−9.3	**119.0**	−6.0	−9.3	**119.0**	−6.0
**$2a^{iPr}$**	−32.1	−4.9	−32.1	−4.9	−7.5	**118.1**	−6.5	−7.5	**118.1**	−6.5
**$2b^{iPr}$**	−29.9	2.8	−29.9	2.8	−7.7	**118.3**	−11.7	−11.7	**118.3**	−7.7
**$2c^{iPr}$**	−30.5	−1.5	−31.9	−5.0	−10.4	**36.8**	−7.5	**46.9**	−19.5	−11.3
**2^{MIC}**	−29.5	−2.3	−29.5	−2.3	−8.1	**111.2**	−6.5	−8.1	**111.2**	−6.5
**$[3a^{Dipp}]^+$**	−29.2	−6.1	−29.2	10.3	−7.8	−33.4	**44.4**	−7.8	**107.5**	−13.7
**$[3a^{iPr}]^+$**	−30.1	−2.4	−48.6	−3.8	−19.7	**−3.0**	−25.1	−6.8	**4.0**	−21.7
**$[3b^{iPr}]^+$**	−29.4	−2.2	−46.0	−2.2	−19.9	**−0.3**	−7.0	−7.0	**4.9**	−16.2
**$[3c^{iPr}]^+$**	−25.4	3.5	−25.9	−0.9	−19.9	**37.8**	−0.9	**45.3**	−19.9	−6.4

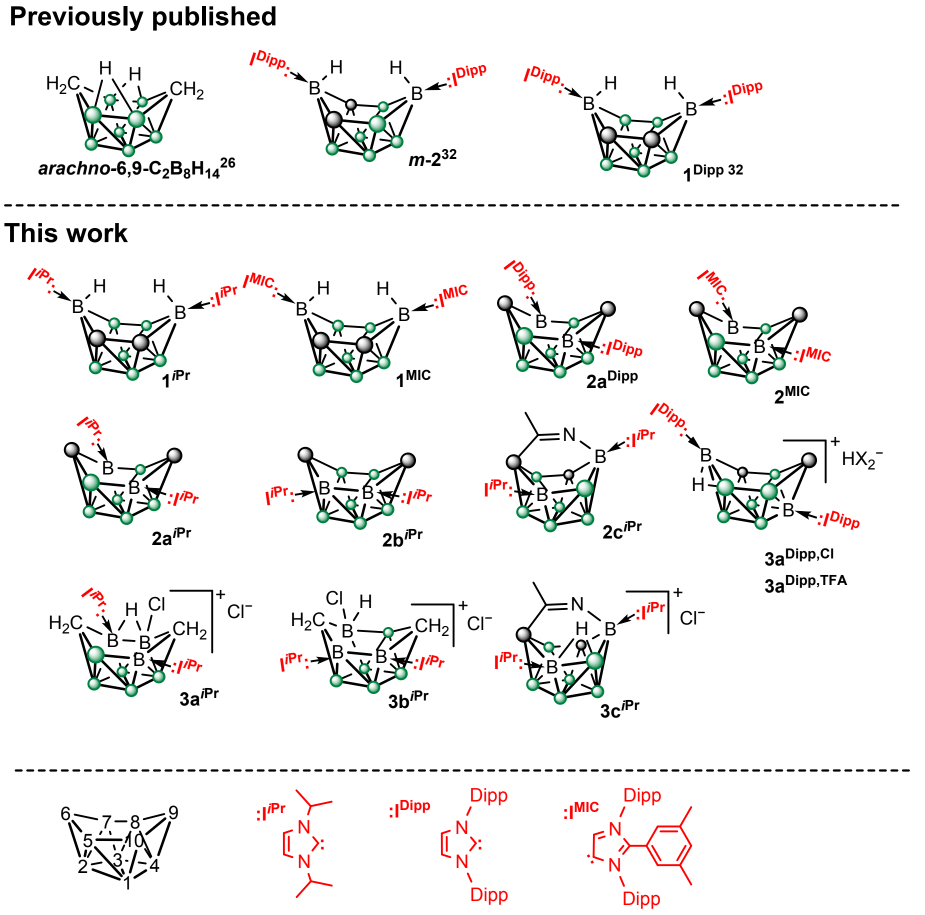

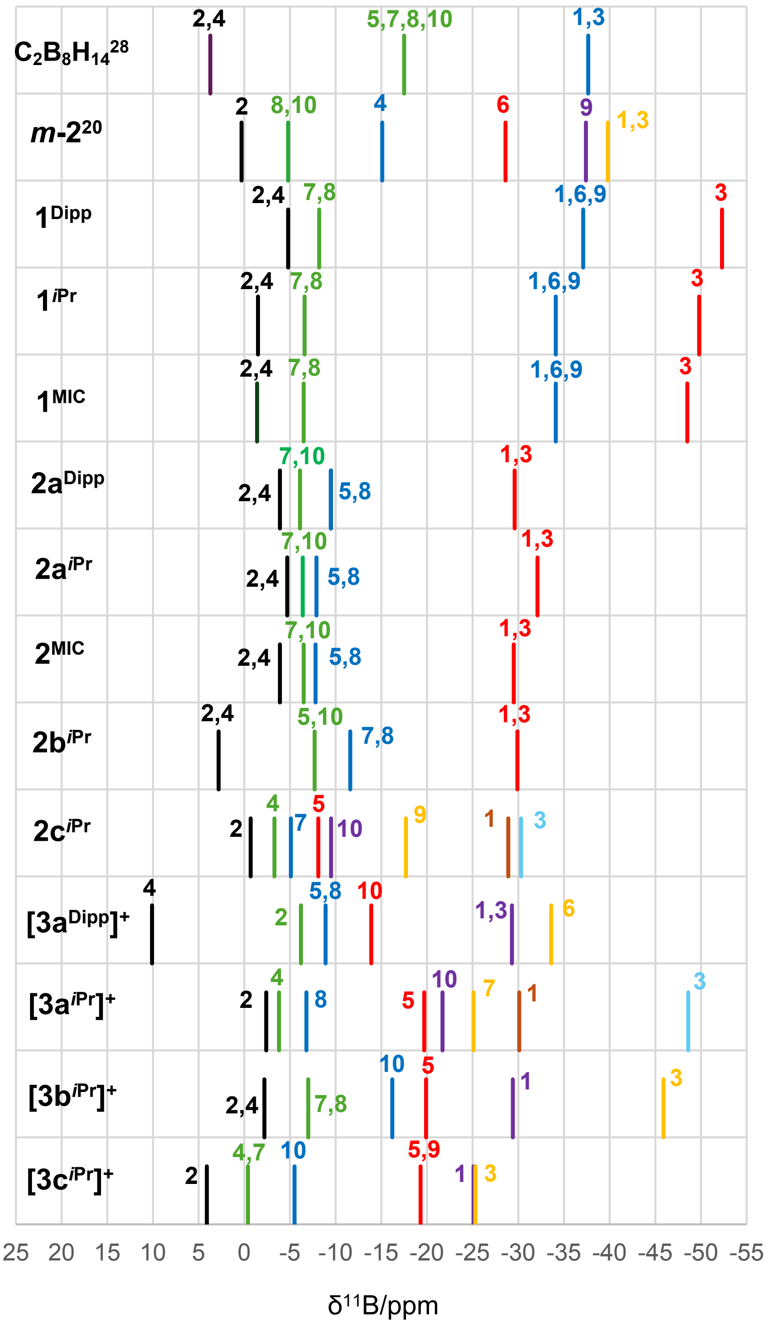

Figure S86. A ^{11}B NMR correlation diagram.

Crystallographic Data

The molecular structures of **[3a^{Dipp}][HCl$_2$]**, **[3a^{Dipp}][H(TFA)$_2$]** and **[3b^{iPr}]Cl** exhibited static disorder of the carborane cages. The structures were solved and refined with lower symmetry (*P*-1) in order to obtain as relevant models as possible. Moreover, in **[3a^{Dipp}][HCl$_2$]** and **[3b^{iPr}]Cl** disorders of HCl_2^- and skeletal Cl atom, respectively, were modelled. Structures of **2a^{iPr}**, **2a^{MIC}** and **3^{MIC}** contain disordered solvent molecules or *i*Pr groups, which were treated by standard methods (spited to two positions: ether, THF or acetonitrile). In **2b^{Dipp}**, twelve *n*-hexane solvent molecules per unit cell were masked in accessible voids of 3074 Å^3 by PLATON/SQUEEZE program.[S1]

The molecular structures of both **1**^{MIC} and **2**^{MIC} have similar geometrical parameters with the exception of the angles B2–B6–C11 (106.48(8)°) and B4–B9–C46 (106.06(8)°) in **1**^{MIC}**,** which are narrower than B2–C6–H6 and B4–C9–H9 in **2**^{MIC}. This difference is clearly a consequence of the presence of both hydrogen atoms H6 and H9, resembling the structure of *arachno*-6,9-$C_2B_8H_{14}$, which has the angles of 105.67°.

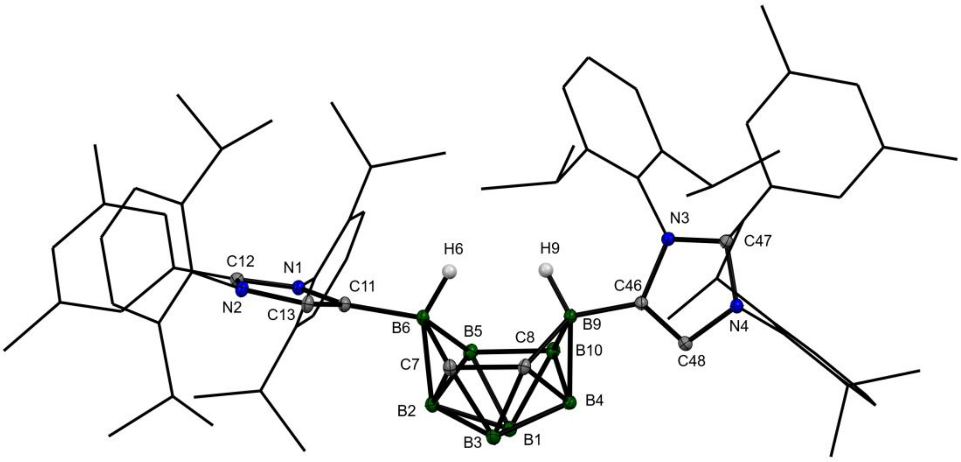

Figure S87. The molecular structure of **1**^{MIC}. ORTEP diagrams, 40% probability level; the 2,6-di*iso*propyl and 3,5-dimethylphenyl groups are shown as wireframes for clarity. Selected interatomic distances [Å] and angles [°]: B5–B6 1.9355(17), B6–C7 1.6300(15), C7–C8 1.5251(14), C8–B9 1.6312(15), B9–B10 1.9547(17), B10–B5 1.6924(17), B6–C11 1.6044(15), B9–C46 1.6000(15), B2–B6–C11 106.48(8)°, B4–B9–C46 106.06(8)°.

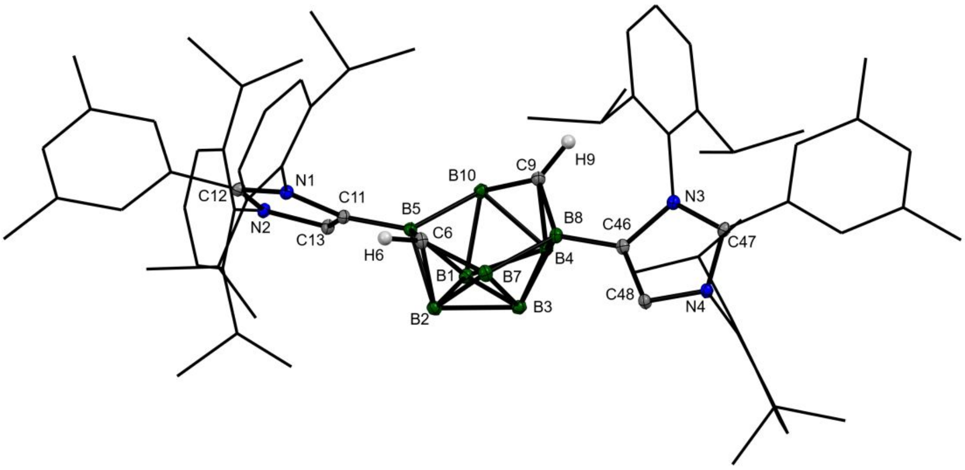

Figure S88. The molecular structure of **2**^{MIC}. ORTEP diagrams, 40% probability level; the 2,6-di*iso*propyl and 3,5-dimethylphenyl groups are shown as wireframes for clarity. Selected interatomic distances [Å]: B5–C6 1.530(2), C6–B7 1.533(2), B7–B8 1.881(2), B8–C9 1.532(2), C9–B10 1.533(2), B10–B5 1.881(2), B5–C11 1.581(2), B8–C46 1.581(2).

Table S2. Crystal data and structure refinement for **1**[MIC].

Crystal data	
Chemical formula	$C_{72}H_{98}B_8N_4·2(C_2H_3N)$
M_r	1188.13
Crystal system, space group	Triclinic, *P-1*
Temperature (K)	150
a, *b*, *c* (Å)	11.9826(3), 14.7660(4), 21.4942(6)
α, β, γ (°)	87.719(1), 83.341(1), 82.416(1)
V (Å^3)	3743.22(17)
Z	2
Radiation type	Mo Kα
μ (mm^{-1})	0.06
Crystal size (mm)	0.39 × 0.19 × 0.18
Data collection	
Diffractometer	Bruker D8 – Venture
Absorption correction	Multi-scan<br>*SADABS2016/2* – Bruker AXS area detector scaling and absorption correction Reference: Krause, L., Herbst-Irmer, R., Sheldrick G.M. & Stalke D., J. Appl. Cryst. 48 (2015) 3–10.
T_{min}, T_{max}	0.712, 0.746
Number of measured, independent and observed [$I > 2σ(I)$] reflections	257828, 15089, 12400
R_{int}	0.078
$(\sin θ/λ)_{max}$ (Å^{-1})	0.628
Refinement	
$R[F^2 > 2σ(F^2)]$, $wR(F^2)$, S	0.041, 0.114, 1.06
Number of reflections	15089
Number of parameters	828
Number of restraints	765
H-atom treatment	H atoms treated by a mixture of independent and constrained refinement
$Δρ_{max}$, $Δρ_{min}$ (e Å^{-3})	0.45, −0.26

Table S3. Crystal data and structure refinement for **2a**$^{\mathbf{Dipp}}$.

Crystal data	
Chemical formula	$C_{56}H_{80}B_8N_4$
M_r	895.72
Crystal system, space group	Monoclinic, *C*2/c
Temperature (K)	150
a, *b*, *c* (Å)	27.4934(4), 10.5724(2), 20.1390(3)
α, β, γ (°)	111.775(1)
V (Å^3)	5436.14(16)
Z	4
Radiation type	Mo Kα
μ (mm^{-1})	0.06
Crystal size (mm)	0.26 × 0.15 × 0.10
Data collection	
Diffractometer	Bruker D8 – Venture
Absorption correction	Multi-scan *SADABS2016/2* – Bruker AXS area detector scaling and absorption correction Reference: Krause, L., Herbst-Irmer, R., Sheldrick G.M. & Stalke D., J. Appl. Cryst. 48 (2015) 3–10.
T_{min}, T_{max}	0.725, 0.746
Number of measured, independent and observed [$I > 2\sigma(I)$] reflections	59015, 6752, 5629
R_{int}	0.048
$(\sin \theta/\lambda)_{max}$ (Å^{-1})	0.667
Refinement	
$R[F^2 > 2\sigma(F^2)]$, $wR(F^2)$, S	0.043, 0.123, 1.03
Number of reflections	6752
Number of parameters	318
Number of restraints	276
H-atom treatment	H atoms treated by a mixture of independent and constrained refinement
$\Delta\rho_{max}$, $\Delta\rho_{min}$ (e Å^{-3})	0.28, −0.26

Table S4. Crystal data and structure refinement for **2b**$^{\mathbf{Dipp}}$.

Crystal data	
Chemical formula	$C_{56}H_{80}B_8N_4 \cdot 1.5(C_6H_{14})$
M_r	1024.97
Crystal system, space group	Monoclinic, $P2_1/n$
Temperature (K)	150
a, *b*, *c* (Å)	17.5882(6), 29.9446(11), 25.1791(11)
α, β, γ (°)	100.849(1)
V (Å^3)	13024.1(9)
Z	8
Radiation type	Mo Kα
μ (mm^{-1})	0.06
Crystal size (mm)	0.48 × 0.26 × 0.24
Data collection	
Diffractometer	Bruker D8 – Venture
Absorption correction	Multi-scan<br>*SADABS2016/2* – Bruker AXS area detector scaling and absorption correction
T_{min}, T_{max}	0.707, 0.744
Number of measured, independent and observed [$I > 2\sigma(I)$] reflections	337424, 22862, 16349
R_{int}	0.116
$(\sin \theta/\lambda)_{max}$ (Å^{-1})	0.595
Refinement	
$R[F^2 > 2\sigma(F^2)]$, $wR(F^2)$, S	0.071, 0.227, 1.10
Number of reflections	22862
Number of parameters	1260
Number of restraints	1246
H-atom treatment	H atoms treated by a mixture of independent and constrained refinement
$\Delta\rho_{max}$, $\Delta\rho_{min}$ (e Å^{-3})	1.13, −0.41

Table S5. Crystal data and structure refinement for **$2a^{iPr}$**.

Crystal data	
Chemical formula	$C_{20}H_{40}B_8N_4 \cdot C_4H_{10}O$
M_r	497.16
Crystal system, space group	Monoclinic, $P2_1/n$
Temperature (K)	150
a, *b*, *c* (Å)	13.7228(5), 14.7720(5), 16.4574(5)
α, β, γ (°)	100.829(1)
V (Å^3)	3276.72(19)
Z	4
Radiation type	Mo Kα
μ (mm^{-1})	0.06
Crystal size (mm)	0.26 × 0.20 × 0.17
Data collection	
Diffractometer	Bruker D8 – Venture
Absorption correction	Multi-scan<br>*SADABS2016/2* – Bruker AXS area detector scaling and absorption correction
T_{min}, T_{max}	0.702, 0.736
Number of measured, independent and observed [$I > 2\sigma(I)$] reflections	78961, 6415, 5019
R_{int}	0.093
$(\sin \theta/\lambda)_{max}$ (Å^{-1})	0.617
Refinement	
$R[F^2 > 2\sigma(F^2)]$, $wR(F^2)$, S	0.079, 0.240, 1.04
Number of reflections	6415
Number of parameters	384
Number of restraints	374
H-atom treatment	H-atom parameters constrained
$\Delta\rho_{max}$, $\Delta\rho_{min}$ (e Å^{-3})	0.48, −0.63

Table S6. Crystal data and structure refinement for **2b^{iPr}**.

Crystal data	
Chemical formula	$C_{20}H_{40}B_8N_4$
M_r	423.04
Crystal system, space group	Monoclinic, $P2_1/n$
Temperature (K)	150
a, *b*, *c* (Å)	9.0780(3), 16.4861(4), 17.1193(5)
α, β, γ (°)	97.004(1)
V (Å^3)	2542.97(13)
Z	4
Radiation type	Mo Kα
μ (mm^{-1})	0.06
Crystal size (mm)	0.47 × 0.43 × 0.22
Data collection	
Diffractometer	Bruker D8 – Venture
Absorption correction	Multi-scan<br>*SADABS2016/2* – Bruker AXS area detector scaling and absorption correction
T_{min}, T_{max}	0.620, 0.746
Number of measured, independent and observed [$I > 2\sigma(I)$] reflections	81504, 6293, 5614
R_{int}	0.052
$(\sin \theta/\lambda)_{max}$ (Å^{-1})	0.667
Refinement	
$R[F^2 > 2\sigma(F^2)]$, $wR(F^2)$, S	0.053, 0.140, 1.05
Number of reflections	6293
Number of parameters	297
Number of restraints	318
H-atom treatment	H-atom parameters constrained
$\Delta\rho_{max}$, $\Delta\rho_{min}$ (e Å^{-3})	0.59, −0.55

Table S7. Crystal data and structure refinement for **2c^{iPr}**.

Crystal data	
Chemical formula	$C_{22}H_{43}B_8N_5 \cdot C_2H_3N$
M_r	505.15
Crystal system, space group	Monoclinic, C_2/c
Temperature (K)	150
a, *b*, *c* (Å)	35.6336(10), 9.8917(3), 18.8253(5)
α, β, γ (°)	111.757(2)
V (Å^3)	6162.8(3)
Z	8
Radiation type	Mo Kα
μ (mm^{-1})	0.06
Crystal size (mm)	0.35 × 0.30 × 0.07
Data collection	
Diffractometer	Bruker D8 – Venture
Absorption correction	Multi-scan<br>*SADABS2016/2* – Bruker AXS area detector scaling and absorption correction
T_{min}, T_{max}	0.716, 0.746
Number of measured, independent and observed [$I > 2\sigma(I)$] reflections	200044, 7647, 6202
R_{int}	0.081
$(\sin \theta/\lambda)_{max}$ (Å^{-1})	0.667
Refinement	
$R[F^2 > 2\sigma(F^2)]$, $wR(F^2)$, S	0.049, 0.149, 1.06
Number of reflections	7647
Number of parameters	356
Number of restraints	369
H-atom treatment	H atoms treated by a mixture of independent and constrained refinement
$\Delta\rho_{max}$, $\Delta\rho_{min}$ (e Å^{-3})	0.60, −0.57

Table S8. Crystal data and structure refinement for **2a^{iPr*}**.

Crystal data	
Chemical formula	$C_{20}H_{40}B_8N_4 \cdot 2(CH_2Cl_2)$
M_r	592.89
Crystal system, space group	Monoclinic, $P2_1/n$
Temperature (K)	150
a, *b*, *c* (Å)	10.4333(2), 13.8064(3), 22.9939(5)
α, β, γ (°)	93.665(1)
V (Å^3)	3305.41(12)
Z	4
Radiation type	Mo Kα
μ (mm^{-1})	0.38
Crystal size (mm)	0.30 × 0.16 × 0.10
Data collection	
Diffractometer	Bruker D8 – Venture
Absorption correction	Multi-scan<br>*SADABS2016/2* – Bruker AXS area detector scaling and absorption correction
T_{min}, T_{max}	0.724, 0.746
Number of measured, independent and observed [$I > 2\sigma(I)$] reflections	211573, 8184, 7173
R_{int}	0.053
$(\sin \theta/\lambda)_{max}$ (Å^{-1})	0.667
Refinement	
$R[F^2 > 2\sigma(F^2)]$, $wR(F^2)$, S	0.043, 0.123, 1.04
Number of reflections	8184
Number of parameters	351
Number of restraints	332
H-atom treatment	H-atom parameters constrained
$\Delta\rho_{max}$, $\Delta\rho_{min}$ (e Å^{-3})	0.66, −0.63

Table S9. Crystal data and structure refinement for **2**$^{\mathbf{MIC}}$.

Crystal data	
Chemical formula	$C_{72}H_{96}B_8N_4 \cdot 2(C_4H_8O)$
M_r	1248.21
Crystal system, space group	Triclinic, *P-1*
Temperature (K)	150
a, *b*, *c* (Å)	10.5269(3), 19.2109(5), 19.2109(4)
α, β, γ (°)	90.267(1), 90.968(1), 103.705(1)
V (Å^3)	3773.74(17)
Z	2
Radiation type	Mo Kα
μ (mm^{-1})	0.06
Crystal size (mm)	0.31 × 0.29 × 0.23
Data collection	
Diffractometer	Bruker D8 – Venture
Absorption correction	Multi-scan *SADABS2016/2* – Bruker AXS area detector scaling and absorption correction Reference: Krause, L., Herbst-Irmer, R., Sheldrick G.M. & Stalke D., J. Appl. Cryst. 48 (2015) 3–10.
T_{min}, T_{max}	0.716, 0.746
Number of measured, independent and observed [$I > 2\sigma(I)$] reflections	189185, 13198, 11307
R_{int}	0.060
$(\sin \theta/\lambda)_{max}$ (Å^{-1})	0.595
Refinement	
$R[F^2 > 2\sigma(F^2)]$, $wR(F^2)$, S	0.052, 0.141, 1.07
Number of reflections	13198
Number of parameters	849
Number of restraints	876
H-atom treatment	H-atom parameters constrained
$\Delta\rho_{max}$, $\Delta\rho_{min}$ (e Å^{-3})	0.54, −0.48

Table S10. Crystal data and structure refinement for **[3a^{Dipp}][HCl$_2$]**.

Crystal data	
Chemical formula	$C_{56}H_{81}B_8N_4 \cdot ClH \cdot Cl$
M_r	968.63
Crystal system, space group	Triclinic, *P1*
Temperature (K)	150
a, *b*, *c* (Å)	10.2441(3), 10.7731(3), 14.9078(4)
α, β, γ (°)	71.564(1), 71.233(1), 74.129(1)
V (Å^3)	1450.56(7)
Z	1
Radiation type	Mo Kα
μ (mm^{-1})	0.15
Crystal size (mm)	0.46 × 0.41 × 0.22
Data collection	
Diffractometer	Bruker D8 – Venture
Absorption correction	Multi-scan *SADABS2016/2* – Bruker AXS area detector scaling and absorption correction Reference: Krause, L., Herbst-Irmer, R., Sheldrick G.M. & Stalke D., J. Appl. Cryst. 48 (2015) 3–10.
T_{min}, T_{max}	0.705, 0.746
Number of measured, independent and observed [$I > 2\sigma(I)$] reflections	99351, 14264, 11653
R_{int}	0.049
$(\sin \theta/\lambda)_{max}$ (Å^{-1})	0.667
Refinement	
$R[F^2 > 2\sigma(F^2)]$, $wR(F^2)$, S	0.097, 0.268, 1.09
Number of reflections	14264
Number of parameters	639
Number of restraints	772
H-atom treatment	H-atom parameters constrained
$\Delta\rho_{max}$, $\Delta\rho_{min}$ (e Å^{-3})	1.66, −1.00
Absolute structure	Refined as an inversion twin.
Absolute structure parameter	0.4(2)

Table S11. Crystal data and structure refinement for **[3a^{Dipp}][H(TFA)$_2$]**.

Crystal data	
Chemical formula	$C_{56}H_{81}B_8N_4 \cdot C_2HF_3O_2 \cdot C_2F_3O_2$
M_r	1123.77
Crystal system, space group	Triclinic, *P1*
Temperature (K)	150
a, *b*, *c* (Å)	9.8828(5), 11.0420(6), 15.2650(9)
α, β, γ (°)	110.592(2), 90.693(2), 94.101(2)
V (Å^3)	1554.14(15)
Z	1
Radiation type	Mo Kα
μ (mm^{-1})	0.08
Crystal size (mm)	0.32 × 0.30 × 0.02
Data collection	
Diffractometer	Bruker D8 – Venture
Absorption correction	Multi-scan<br>*SADABS2016/2* – Bruker AXS area detector scaling and absorption correction Reference: Krause, L., Herbst-Irmer, R., Sheldrick G.M. & Stalke D., J. Appl. Cryst. 48 (2015) 3–10.
T_{min}, T_{max}	0.620, 0.745
Number of measured, independent and observed [$I > 2\sigma(I)$] reflections	50143, 10841, 7116
R_{int}	0.118
$(\sin \theta/\lambda)_{max}$ (Å^{-1})	0.606
Refinement	
$R[F^2 > 2\sigma(F^2)]$, $wR(F^2)$, S	0.064, 0.184, 1.04
Number of reflections	10841
Number of parameters	822
Number of restraints	810
H-atom treatment	H-atoms treated by a mixture of independent and constrained refinement
$\Delta\rho_{max}$, $\Delta\rho_{min}$ (e Å^{-3})	0.38, −0.30
Absolute structure	Flack x determined using 2719 quotients [(I+)-(I-)]/[(I+)+(I-)]<br>(Parsons, Flack and Wagner, Acta Cryst. B69 (2013) 249–259).
Absolute structure parameter	1.3(10)

Table S12. Crystal data and structure refinement for **[3b^{iPr}]Cl**.

Crystal data	
Chemical formula	$C_{20}H_{39}B_8N_4Cl{\cdot}C_2H_3N{\cdot}Cl$
M_r	537.01
Crystal system, space group	Triclinic, *P*1
Temperature (K)	150
a, *b*, *c* (Å)	10.0217(10), 10.0215(10), 10.2106(10)
α, β, γ (°)	63.715(4), 63.200(4), 62.503(4)
V (Å^3)	775.44(14)
Z	1
Radiation type	Mo Kα
μ (mm^{-1})	0.23
Crystal size (mm)	0.21 × 0.21 × 0.09
Data collection	
Diffractometer	Bruker D8 – Venture
Absorption correction	Multi-scan<br>*SADABS2016/2* – Bruker AXS area detector scaling and absorption correction
T_{min}, T_{max}	0.651, 0.746
Numer of measured, independent and observed [$I > 2\sigma(I)$] reflections	24561, 5188, 5110
R_{int}	0.047
$(\sin \theta/\lambda)_{max}$ (Å^{-1})	0.595
Refinement	
$R[F^2 > 2\sigma(F^2)]$, $wR(F^2)$, S	0.108, 0.298, 1.06
Number of reflections	5188
Number of parameters	352
Number of restraints	350
H-atom treatment	H-atom parameters constrained
$\Delta\rho_{max}$, $\Delta\rho_{min}$ (e Å^{-3})	1.00, −0.66
Absolute structure	Refined as an inversion twin
Absolute structure parameter	0.2(2)

Table S13. Crystal data and structure refinement for **[3c^{iPr}][HCl$_2$]**.

Crystal data	
Chemical formula	$C_{22}H_{44}B_8N_5 \cdot C_4H_8O \cdot Cl \cdot ClH$
M_r	609.11
Crystal system, space group	Triclinic, *P-1*
Temperature (K)	150
a, *b*, *c* (Å)	10.8724(2), 12.2137(3), 14.5304(4)
α, β, γ (°)	108.793(1), 106.926(1), 93.217(1)
V (Å^3)	1723.39(7)
Z	2
Radiation type	Mo Kα
μ (mm^{-1})	0.22
Crystal size (mm)	0.41 × 0.28 × 0.23
**Data collection**	
Diffractometer	Bruker D8 – Venture
Absorption correction	Multi-scan<br>*SADABS2016/2* – Bruker AXS area detector scaling and absorption correction
T_{min}, T_{max}	0.690, 0.746
Number of measured, independent and observed [$I > 2\sigma(I)$] reflections	92389, 7473, 6712
R_{int}	0.042
$(\sin \theta/\lambda)_{max}$ (Å^{-1})	0.639
**Refinement**	
$R[F^2 > 2\sigma(F^2)]$, $wR(F^2)$, S	0.043, 0.122, 1.03
Number of reflections	7473
Number of parameters	400
Number of restraints	390
H-atom treatment	H atoms treated by a mixture of independent and constrained refinement
$\Delta\rho_{max}$, $\Delta\rho_{min}$ (e Å^{-3})	0.89, −0.52

Theoretical Investigations

DFT Calculations

The theoretical mechanism of this process was investigated at the B3LYP-D3(BJ)/def2-TZVP level of theory with implicit solvation (PCM) for both **1**^{iPr} and **1**^{Dipp} (Figure S89). In the first stage of the process, two hydrogen atoms (H1 and H2) are eliminated via TS-1. Following this step, the boron atoms B1 and B2 lose one coordination bond, and the subsequent rearrangement is directed toward restoring their five-coordinate environment. In the second stage (TS-2), B3 shifts toward B2, resulting in the formation of a five-membered ring B1–C1–C2–B2–B3 in the upper layer. In this configuration, a "cavity" arises between the upper and lower layers, which is closed upon the interaction of B3…B4…B5 (TS-3). Further displacement of the B5 atom (INT-3) leads to the formation of a new "cavity" between B1…B5…B6, which also closes via TS-4. Subsequently, the entire system returns to the "boat"-type configuration (INT-4). Although INT-4 (*i*Pr) exhibits a negative Gibbs free energy, this intermediate is thermodynamically less stable than the final product **2a**^{iPr}, which is formed through the closure of the C1–B3 bond (TS-5) followed by the cleavage of the C1–C2 bond (TS-6). A similar rearrangement occurs for INT-4 (Dipp), leading to the formation of **2a**^{Dipp}. Regarding the steric influence of the substituents on the reaction energetics, the difference between the corresponding states for *i*Pr and Dipp varies in the range of 2.16–22.77 kcal/mol. Therefore, the reaction involving NHC^{iPr} requires lower energy than in the case of NHC^{Dipp}, which is consistent with the experimental observations: The formation of **2a**^{iPr} proceeds at room temperature, whereas the corresponding reaction forming **2a**^{Dipp} requires elevated temperatures (60–100 °C).

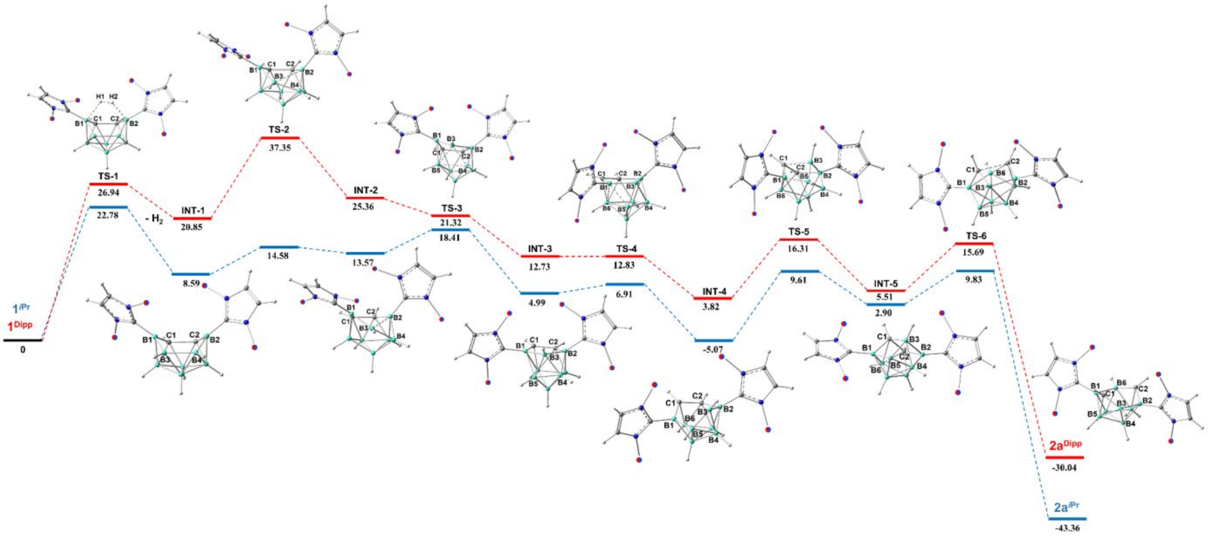

Figure S89. The DFT-estimated Gibbs free energy profile (kcal/mol) for the rearrangement mechanisms of $\mathbf{1}^{\mathbf{\mathit{i}Pr}}$ (blue) and $\mathbf{1}^{\mathbf{Dipp}}$ (red), leading to $\mathbf{2a}^{\mathbf{\mathit{i}Pr}}$ and $\mathbf{2a}^{\mathbf{Dipp}}$, respectively. The red–blue dots correspond to 1,3-(2,6-*i*$Pr_2C_6H_3$)-imidazole-2-ylidene and 1,3-di*iso*propyl-1H-imidazol-3-ium-2- ylidene (also labeled as IR).

Figure S90 illustrates the subsequent reaction pathways of the protonation of $\mathbf{2a}^{\mathbf{Dipp}}$ with HCl, leading to **[3a$^{\mathbf{Dipp}}$][Cl]**, followed by deprotonation, yielding a mixture of $\mathbf{2a}^{\mathbf{Dipp}}$ and $\mathbf{2b}^{\mathbf{Dipp}}$, which is transformed into pure $\mathbf{2a}^{\mathbf{Dipp}}$ at room temperature. The mechanistic details of this transformation are beyond the scope of the present work.

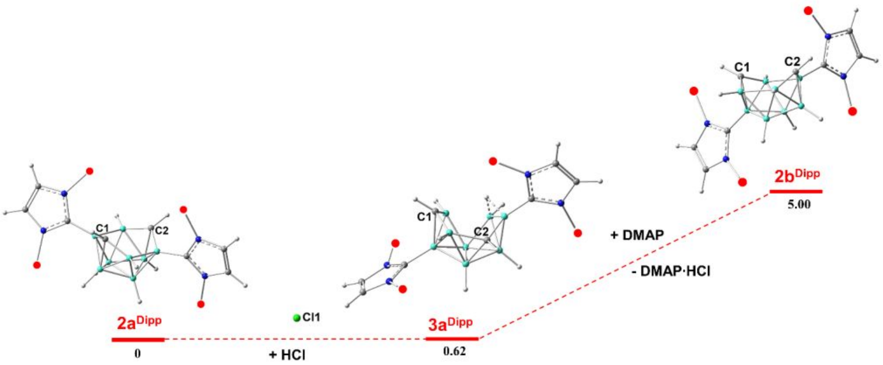

Figure S90. The DFT-estimated Gibbs free energy profile (kcal/mol) for the rearrangement mechanism of $\mathbf{2a}^{\mathbf{Dipp}}$, leading to $\mathbf{2b}^{\mathbf{Dipp}}$. The red dots correspond to Dipp = 2,6-*i*$Pr_2C_6H_3$.

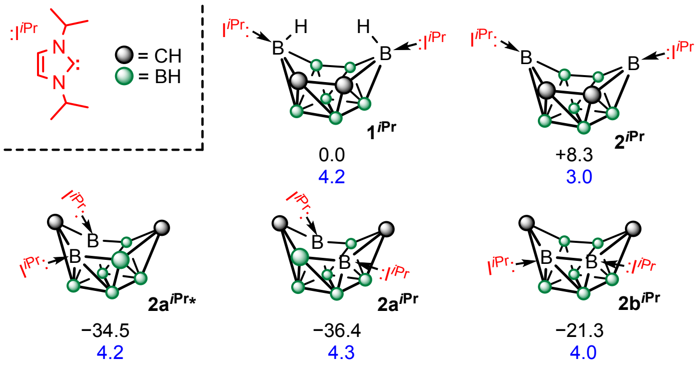

Figure S91. Calculated free Gibbs energies (in black, kcal/mol) and HOMO–LUMO gaps (in blue, eV) at B3LYP/def2-TZVP level of theory.

References

S1. Spek, A. L. PLATON SQUEEZE: a tool for the calculation of the disordered solvent contribution to the calculated structure factors. *Acta Cryst.* **2015**, *C71*(1), 9–18. DOI: 10.1107/S2053229614024929.
